# Sorghum *Dw1*, an agronomically important gene for lodging resistance, encodes a novel protein involved in cell proliferation

**DOI:** 10.1038/srep28366

**Published:** 2016-06-22

**Authors:** Miki Yamaguchi, Haruka Fujimoto, Ko Hirano, Satoko Araki-Nakamura, Kozue Ohmae-Shinohara, Akihiro Fujii, Masako Tsunashima, Xian Jun Song, Yusuke Ito, Rie Nagae, Jianzhong Wu, Hiroshi Mizuno, Jun-ichi Yonemaru, Takashi Matsumoto, Hidemi Kitano, Makoto Matsuoka, Shigemitsu Kasuga, Takashi Sazuka

**Affiliations:** 1 Bioscience and Biotechnology Center, Nagoya University, Furou-cho, Nagoya, Aichi 464-8601, Japan; 2 Plant Innovation Center, Japan Tobacco Inc., Iwata, Shizuoka 438–0802, Japan; 3 National Institute of Agrobiological Sciences, Kannondai, Tsukuba, Ibaraki 305-8602, Japan; 4 Education and Research Center of Alpine Field Science, Faculty of Agriculture, Shinshu University, Minamiminowa, Nagano 399-4598, Japan

## Abstract

Semi-dwarfing genes have contributed to enhanced lodging resistance, resulting in increased crop productivity. In the history of grain sorghum breeding, the spontaneous mutation*, dw1* found in Memphis in 1905, was the first widely used semi-dwarfing gene. Here, we report the identification and characterization of *Dw1*. We performed quantitative trait locus (QTL) analysis and cloning, and revealed that *Dw1* encodes a novel uncharacterized protein. Knockdown or T-DNA insertion lines of orthologous genes in rice and Arabidopsis also showed semi-dwarfism similar to that of a nearly isogenic line (NIL) carrying *dw1* (NIL-*dw1*) of sorghum. A histological analysis of the NIL-*dw1* revealed that the longitudinal parenchymal cell lengths of the internode were almost the same between NIL-*dw1* and wildtype, while the number of cells per internode was significantly reduced in NIL-*dw1.* NIL-*dw1dw3*, carrying both *dw1* and *dw3* (involved in auxin transport), showed a synergistic phenotype. These observations demonstrate that the *dw1* reduced the cell proliferation activity in the internodes, and the synergistic effect of *dw1* and *dw3* contributes to improved lodging resistance and mechanical harvesting.

A C4 crop, sorghum [*Sorghum bicolor* (L.) Moench] is the fifth most important cereal crop and is the dietary staple of more than 500 million people in 98 countries^1^,^2^. This crop is drought tolerant and can be grown in semi-arid conditions, where maize, wheat, and rice cannot be grown because of water scarcity^3^.

Recently, some agronomically important genes of sorghum have been isolated using comparative genome studies between the sorghum and other crops. In the case of flowering time, *PSEUDORESPONSE REGULATOR PROTEIN (SbPRR37)/Ma1*^4^*, PHYTOCHROME B (SbPHYB)/Ma3*^5^*, PHYTOCHROME C (SbPHYC)/Ma5, EARLY HEADING DATE 1 (SbEHD1), FLOWERING LOCUS T (sbFT)/SbCN15, SbCN8, SbCN12*^6^*, CONSTANS (SbCO)*^7^, and *GRAIN NUMBER, PLANT HEIGHT AND HEADING DATE 7 (SbGHD7)/Ma6*^8^, have been identified based on their synteny or homology with the genes of other plant species. In contrast, however, the cloning and analysis of dwarfing genes has not advanced. It may be that their causal genes, except *Dw3*, do not correlate to previously identified genes in other plants (see below). In sorghum breeding, the four major unlinked dwarfing genes, *Dw1-Dw4*, have been combined to reduce plant height to increase lodging resistance and improve mechanized harvesting^9^. Among these dwarfing genes, *Dw3* has been cloned. It is syntenic to maize *Brachytic2*, which is involved in auxin transport^10^. The remaining dwarfing genes have not been cloned.

The origin of the *dw1* mutation is described in detail^11^. In brief, Dwarf Yellow Milo, a spontaneous mutant derived from Standard Milo, was found in a field at Memphis in 1905, and then the seeds were distributed to famers^11^. The mutant and its derivatives were widely accepted from 1906 until the 1920s in the United States^11^,^12^. Now, many cultivars of grain sorghums carry the *dw1* mutation, indicating its importance for lodging resistance and improved mechanized harvesting. Genetic analyses of *dw1* have been performed by several groups^13^,^14^,^15^,^16^,^17^, and it was hypothesized that a gibberellin (GA) catabolizing gene, *GA 2-oxidase* (*Sobic.009G230800*) might correspond to *dw1* because this gene locates to the *dw1* locus^15^,^16^,^17^. More recently, however, we revealed that there were no differences in the sequences or expression levels of this *GA2ox* gene between the two cultivars ‘Tall White Sooner Milo’, carrying *Dw1*, and ‘Dwarf White Milo’, carrying *dw1*^18^. We also revealed that a deficiency in GA induces not only dwarfism of culm, but also culm bending, inevitably leading to abnormal plant architecture, which is unsuitable for breeding^18^. These results indicated that the sorghum dwarfing genes used for its breeding might have no relation to GA deficiency or insensitivity, unlike the rice and wheat dwarfing genes that contributed to the ‘green revolution’, which encode GA synthesis enzymes and signaling proteins^19^,^20^. Thus, the cloning of *Dw1* would aid in the understanding of a novel molecular mechanism for dwarfism that is useful in breeding. Here, we isolated the *Dw1* gene using a QTL analysis and positional cloning, and revealed that *Dw1* encodes a novel protein. The loss-of-function in rice and Arabidopsis homologs also induces semi-dwarfism in these plants. A histological analysis of a nearly isogenic line carrying *dw1* (NIL-*dw1*) revealed that the longitudinal parenchymal cell lengths of the internode were almost the same between NIL-*dw1* and wildtype, while the number of cells per internode was significantly reduced in NIL-*dw1.* Furthermore, NIL-*dw1dw3*, carrying both *dw1* and *dw3* (involved in auxin transport), showed a synergistic phenotype. Based on these observations, we discuss the interaction between *dw1* and *dw3* from a view point of crop breeding and cell proliferation.

## Results

### QTL analysis

**Figure 1:**
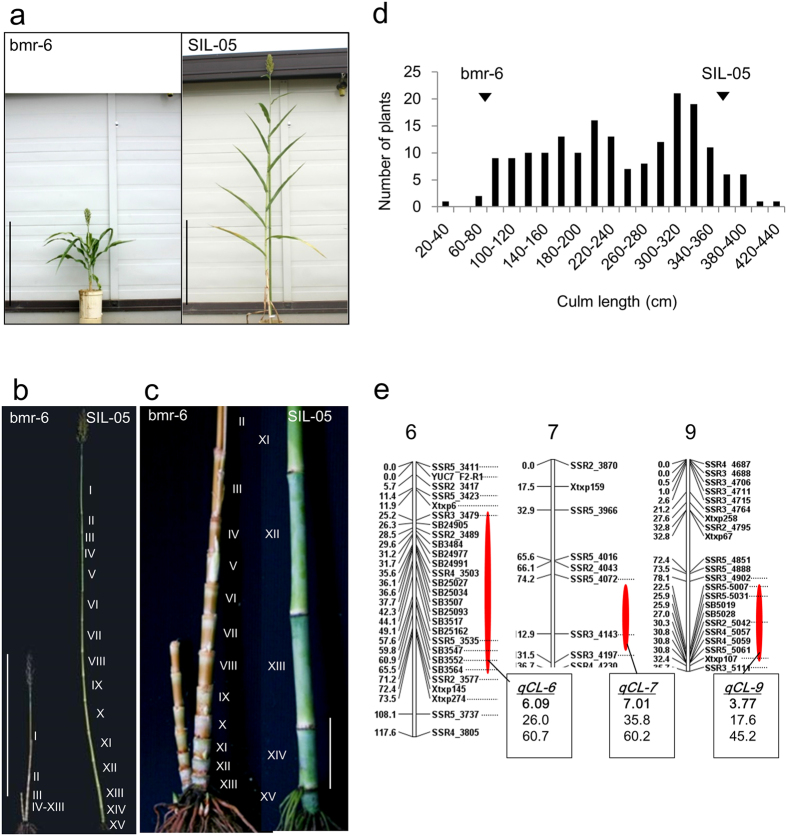
QTL analysis of CL. (**a**) Plant stature of two cultivars used in this study; (Left) Three-dwarf cultivar carrying three dwarfing genes, bmr-6, and (Right) zero-dwarf cultivar carrying no dwarfing genes, SIL-05. Bar = 1 m. (**b**) An elongation pattern of internodes. Bar = 1 m. (**c**) The enlarged views of the basal regions of (**b**). Bar = 10 cm. (**d**) Distribution frequency of CL among 185 F_2_ plants from a cross between bmr-6 and SIL-05. Phenotypic values for bmr-6 and SIL-05 are indicated by arrowheads. (**e**) QTL analysis of CL using 96 F_2_ plants. Only chromosomes 6, 7, and 9, which had log-likelihood value (LOD) score > 3, were presented. The positions and names of the DNA markers used for the analysis are indicated at the left and right sides, respectively. Red ellipses indicate the positions of the QTLs for CL. Boxes contain, in order, the QTL name (bold and underlined), LOD score (bold), percentage of variance-explained, and additive effect, a positive value indicates that the allele from SIL-05 increases CL.

For the QTL analysis, we used two sorghum cultivars, bmr-6 and SIL-05, showing different culm lengths (CLs). bmr-6, which carries three dwarfing genes, including *dw1,* has a ~80 cm CL at the heading stage, whereas SIL-05, which probably carries no dwarfing gene, has a CL of ~365 cm (Fig. 1a,d). When we compared the elongation pattern of internodes, the elongation defect in bmr-6 occurred at all internodes in comparison with the SIL-05 internodes (Fig. 1b,c; Supplementary Fig. S1). We produced F_2_ plants by crossing bmr-6 and SIL-05. The CL values of 185 F_2_ plants were broadly distributed from 37 to 421 cm (Fig. 1d). We performed a QTL analysis on 96 F_2_ plants using 162 molecular markers and detected three major QTLs, which were located on chromosomes 6 (*qCL-6*), 7 (*qCL-7*), and 9 (*qCL-9*) (Fig. 1e and Table 1). The bmr-6 allele of all three QTLs shortened the CL (Table 1).

The locations of these three *qCL*s correspond to those already reported for *dw1-dw3*; that is, *qCL-6* to *dw2, qCL-7* to *dw3,* and *qCL-9* to *dw1.* We sequenced the *Dw3* gene and confirmed that the bmr-6 allele has the same mutation previously reported, indicating that *qCL-7* depends on the *dw3* mutation (Supplementary Fig. S2a). The situation for *qCL-6* is more complex because the *dw2* mutation is tightly linked to a maturation gene, *ma1*^17^,^21^,^22^, which affects the flowering time and consequently the CL. In fact, a strong QTL of flowering time overlaps this region in the same F_2_ plants (Supplementary Fig. S3), and bmr-6 carries the mutation allele of *ma1*, as previously reported^4^ (Supplementary Fig. S2b), indicating that *qCL-6* might be affected by *Ma1*. Therefore, we focused on the isolation of *Dw1* in this study.

### Positional cloning

**Figure 2:**
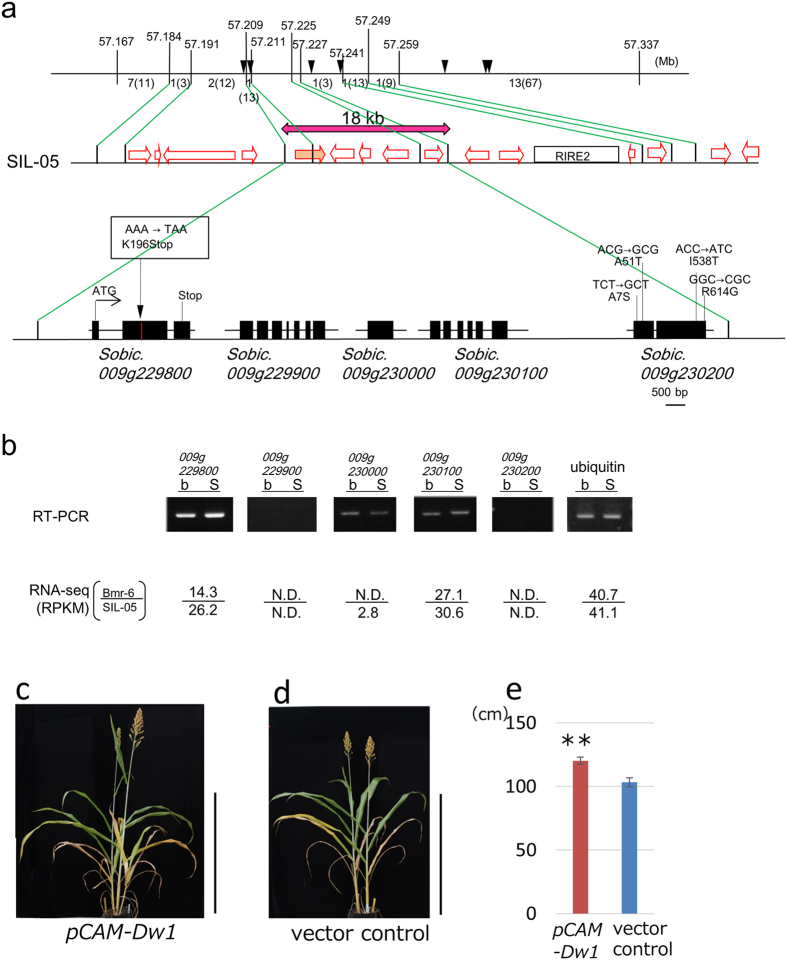
Positional cloning and Phenotypic complementation of the corresponding gene of *qCL-9*. (**a**) High-resolution physical map of *qCL-9*. On the upper horizontal line, vertical lines indicate the positions of DNA markers with their physical positions (Mb) in Phytozome ver.9.1 (*Sorghum bicolor* ver.1.4). Numbers of recombinants are shown between markers. The number of progeny examined in the next generation is presented in parentheses. Arrowheads indicate the physical positions of the DNA markers used in Table 2. The middle horizontal line is a schematic representation of the gene arrangement near the candidate region, which was predicted by the BAC sequence of SIL-05. Open arrows indicate the positions of predicted genes. The mapping analysis narrowed the candidate region to the 18 kb shown by a red double-headed arrow. RIRE2 indicates a gypsy-type retrotransposon. The bottom horizontal line represents an enlarged map of the 18 kb region. There are five predicted genes, whose exons are represented as black boxes. A premature stop codon detected in the bmr-6 genome is indicated in the box. (**b**) The expression level of each gene determined by semi-quantitative RT-PCR. The reads per kilo-base per million reads (RPKM) scores are also shown. N.D. means the sequence was not detected. (**c**) Introduction of DNA fragment encompassing the *Dw1* gene (SIL-05 allele) (*pCAM-Dw1*) into NIL-*dw1* plant Bar = 1 m. (**d**) Empty vector introduced NIL-*dw1* plant was used as a control (vector control). Bar = 1 m. (**e**) The culm lengths of the transgenic plants with *Dw1* gene (left) and empty vector (right). Error bars represent the standard deviation. Double asterisk indicates a significant difference at 1% (P <0.01) as determined by *t*-test.

For the positional cloning of *Dw1*, we generated heterozygous inbred families (HIFs), which are kinds of recombinant inbred lines (F_5_ and F_6_), carrying a segregating locus of *Dw1*. In this experiment, we used two HIFs showing clear differences in CL, dependent on *Dw1* segregation, for further mapping. The genetic analysis, which used ~10,000 plants in two years, narrowed down a candidate region to 18 kb between markers located at 57.209 Mb and 57.227 Mb (Fig. 2a). To compare the genomic sequences of bmr-6 and SIL-05, we constructed bacterial artificial chromosome (BAC) libraries derived from the two cultivars and screened the clones containing the candidate region. Both sequences predicted five genes in the region (Fig. 2a). In *Sobic.009g229800*, there was one single nucleotide polymorphism (SNP) inducing a premature stop codon in bmr-6 (Fig. 2a). In *Sobic.009g230200,* there were four amino acid differences, while the predicted amino acid sequences of the three other genes, *Sobic.009g229900*, *Sobic.009g230000*, and *Sobic.009g230100,* were identical.

We compared the expression levels of these genes using semi-quantitative RT-PCR and RNA-seq analyses on RNAs extracted from elongating internodes (Fig. 2b). The RT-PCR analyses revealed that the expression of *Sobic.009g229800* in bmr-6 was slightly lower than that in SIL-05, while the expression of the other genes was almost same in these cultivars, with no detection of *Sobic.009g229900* or *Sobic.009g230200*. The results of RNA-seq supported the above RT-PCR observations. There were similar expression levels of *Sobic.009g230100*, no, or very low levels, of *Sobic.009g229900*, *Sobic.009g230000*, and *Sobic.009g230200*, and ~50% expression of *Sobic.009g229800* in bmr-6 in comparison with SIL-05.

To confirm that *Sobic.009g229800* corresponds to *qCL-9*, we carried out a complementation experiment using cv. Tx430, which carries the allele of bmr-6 at *qCL-9*. A 4.5 kb DNA fragment containing *Sobic.009g229800* of SIL-05 was introduced into Tx430 by Agrobacterium-mediated transformation. The transformants showed the rescued phenotype, whereas the plants transformed with the empty vector showed dwarfism (Fig. 2c–e). These results strongly suggest that *Sobic.009g229800* is the causal gene for *qCL-9*.

Next, we examined sequences of the candidate 18 kb region in ‘Tall White Sooner Milo’, carrying *Dw1*, and ‘Dwarf White Milo’, carrying *dw1*, because the genotypes of *Dw1* in both cultivars were described in detail previously^12^,^21^. Surprisingly, there were only two SNPs in the region between the two cultivars (Supplementary Fig. S4). One was the causal SNP (the loss-of-function allele) of *qCL-9* in *Sobic.009g229800*, which also existed in ‘Dwarf White Milo’, however, ‘Tall White Sooner Milo’ and ‘Standard Milo’ (mother cultivar of Dwarf Milo) contained the SIL-05 allele (the WT allele). Another SNP was found in an intergenic region. There was no SNP in *Sobic.009g230200*. This result strongly suggests that *Sobic.009g229800* is the causal gene for not only *qCL-9* but also *Dw1*.

### Molecular analysis and biological activity of DW1

Next, we analyzed the characteristics of *Sobic.009g229800/Dw1*. Although its predicted amino acid sequence does not contain any significant characterized domains or sequences, a phylogenetic analysis revealed that there are homologs among *Zea mays*, *Oryza sativa, Brachypodium distachyon*, *Arabidopsis thaliana*, *Selaginella moellendorffii*, and *Physcomitrella patens* (Supplementary Fig. S5), indicating that DW1 is widely conserved among land plants. The amino acid alignment demonstrates that there are several conserved domains in these proteins (Supplementary Fig. S6), although none of them have been characterized. In the sorghum genome, there are two homologous genes, *Sobic.004G349900* and *Sobic.002G138500*, which the phylogenetic analysis categorized into a different sub-group of *Sobic.009g229800* (Supplementary Fig. S5), suggesting that there are at least two sub-groups of this protein family. As *Sobic.009g229800* is the sole member of Sub-group 1 in sorghum, its mutation may induce the dwarf phenotype even if its homologs in sub-group 2 still function.

**Figure 3:**
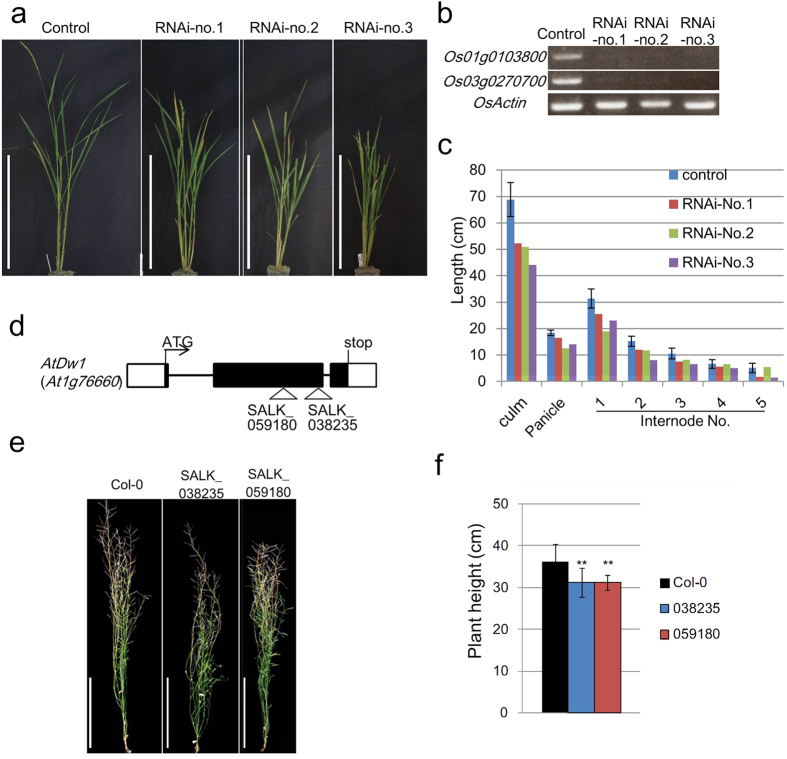
Semi-dwarf phenotypes of *Os01g0103800* and *Os03g0270700* rice RNAi lines, and of *At1g76660* Arabidopsis T-DNA insertion lines. (**a**) Plant stature of *Os01g0103800* and *Os03g0270700* RNAi lines. From left: vector control, RNAi-no.1, RNAi-no.2, and RNAi-no.3. Bar = 50 cm. (**b**) Expression levels of *Os01g0103800*, *Os03g0270700*, and actin (control) in the RNAi and vector control plants. (**c**) Length of culm, panicle, and each internode of the RNAi and vector control plants. (**d**) Positions of T-DNA insertions in *At1g76660* (*AtDw1*), forming two lines (038235 and 059180). Protein coding regions and UTRs are represented by black and white boxes, respectively. Introns are indicated by black bars. ‘ATG’ and ‘Stop’ indicate the initiation and stop codon sites. (**e**) Phenotypes of the T-DNA insertion lines: left, 038235 and right, 059180. Scale bar = 10 cm. (**f**) Plant heights of the T-DNA insertion lines. Error bars represent the standard deviation. Double asterisks indicate significant differences at 1% (P < 0.01) as determined by *t*-test.

We examined the biological functions of the putative orthologs to sorghum *Dw1*, Rice transgenic lines carrying an RNAi construct that targeted the overlapping sequences of *Os01g0103800* and *Os03g0270700* showed semi-dwarf phenotypes with reduced internodes (Fig. 3a–c). Furthermore, the two T-DNA insertion lines of *At1g76660* showed reduced plant heights in comparison with the background plants (Fig. 3d–f). These results confirm the above hypothesis that defects in the *Dw1* orthologs of rice and Arabidopsis cause semi-dwarfism in these plants.

### Importance of *dw1* and selection in sorghum breeding

Next, we studied how widely the *dw1* mutation was used in the breeding of sorghum. There are many SNPs between SIL-05 and bmr-6 within the candidate region of *qCL-9*. We randomly selected seven SNPs, including the causal SNP of *dw1* from 57.208 Mb to 57.273 Mb (Fig. 2a). When we examined the genotypes of these SNPs in ‘Tall White Sooner Milo’ (*Dw1*) and ‘Dwarf White Milo’ (*dw1*) (Table 2), the SNPs of ‘Dwarf White Milo’ were identical to those of bmr-6, suggesting that the causal SNP of *dw1* might be used in the selection process of ‘Dwarf White Milo’. In fact, the 18 kb sequence of the candidate region from ‘Dwarf White Milo’ was completely the same in bmr-6 and in BTx623 (Supplementary Fig. S4).

### Interactions between *Dw1* and *Dw3*

**Figure 4:**
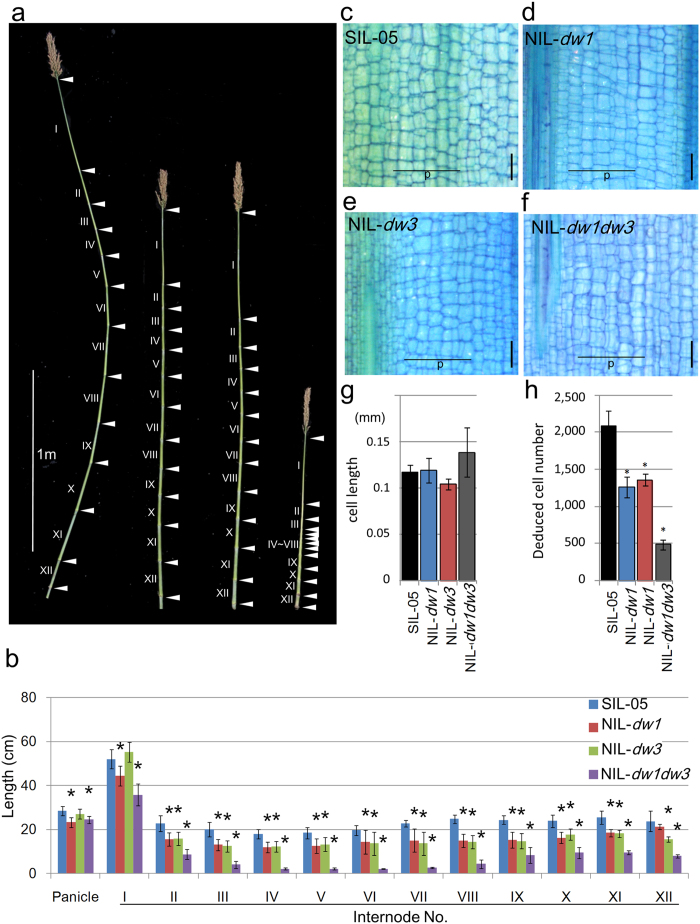
Genetic interactions between *Dw1* and *Dw3*. (**a**) The internode elongation patterns of the NILs and their background lines. From left to right: SIL-05, NIL-*dw1*, NIL-*dw3*, and NIL-*dw1dw3*. Leaves were removed to observe nodes and internodes. Roman numbers indicate the position of the internode (counted from the top). Arrowheads indicate nodes. (**b**) The average lengths of each internode calculated using seven plants. From left to right: SIL-05, NIL-*dw1*, NIL-*dw3*, and NIL-*dw1dw3*. (**c–f**) Longitudinal sections of the middle of internode IX from NILs and SIL-05. The parenchyma cells were examined. Vertical bars = 200 μm. (**g,h**) Length and deduced number of parenchyma cells of internode IX calculated from five cell files of parenchyma cells (shown as “p” in panels c–f). Error bars represent the standard deviation. Single and double asterisks indicate significant differences at 5% (P < 0.05) and 1% (P < 0.01) levels of significance, respectively, as determined by *t*-test.

To compare the effects of *dw1* and/or *dw3* on dwarfism, we produced two NILs, *dw3* alone (NIL-*dw3*) and the double mutant (NIL-*dw1dw3*) in addition to NIL*-dw1* (see above), by the introgression of these mutations into SIL-05 (Supplementary Fig. S7). Interestingly, both NIL-*dw1* and NIL-*dw3* showed a similar elongation pattern, except at the upper (I) and lower (XII) internodes, where *dw3* did not affect the upper internodes but had a greater effect than *dw1* at the lower internodes (Fig. 4a, b). More importantly, *dw1* and *dw3* synergistically function to reduce the internode length, especially in the middle internodes IV–VII. We also performed a histological analysis on these internodes. The longitudinal cell length of the parenchyma was almost the same among the four NILs, while the number of cell per internode was significantly reduced in NIL-*dw1* and NIL-*dw3*, and especially in NIL-*dw1dw3* (Fig. 4c–h). These observations suggest that the mutations of *dw1* and *dw3* decreased the cell proliferation rates, whereas the double mutation had a synergistic effect on the cell proliferation rate.

## Discussion

Semi-dwarfism is tightly associated with lodging resistance and mechanized harvesting, which are important factors in determining crop productivity. To date, some important genes involved in growth processes have been identified to elucidate their mechanisms and applications for molecular breeding. For example, mutations of *semi-dwarf1 (sd1)* in rice^19^,^23^ and *Reduced height (Rht)* in wheat^20^, which encode a GA biosynthesis enzyme and a dominant suppressor protein of GA signal transduction, respectively, have been widely used to improve lodging resistance in these crops, resulting in the success of the “green revolution”. It has also been reported that the semi-dwarfism of barley, *sdw1/denso*, widely introgressed into cultivars grown in Europe, probably results from a defect in an ortholog of rice *SD1*^24^,^25^,^26^. That GA-related mutations have been used for improving lodging resistance in rice, wheat, and barley, demonstrates the importance of decreasing the GA level, or sensitivity to GA, in reducing plant heights. However, in the case of sorghum, such strategies may not be applicable because they induce culm bending, which inevitably causes abnormal plant architecture^18^.

In this study, we isolated a causal gene for the semi-dwarfism of sorghum, which has been widely introgressed into many cultivars, especially those of grain sorghum, using a QTL analysis and positional cloning. We concluded that *Dw1* corresponds to *Sobic.009g229800* due to the following: First, within the candidate region, there is only one gene, *Sobic.009g229800,* which is expressed in elongating internodes and that encodes different amino acid sequences between the parental cultivars, due to a premature stop codon in the bmr-6 gene (Fig. 2a,b). Second, although there are four amino acid differences in *Sobic.009g230200* between the parental cultivars, its expression was hardly detected in elongating internodes by RT-PCR or RNA-seq (Fig. 2a,b). Third, ‘Dwarf White Milo’ (*dw1*) also contains a premature stop codon identical to bmr-6 in *Sobic.009g229800*, whereas ‘Tall White Sooner Milo’ (*Dw1*) does not, while the genome sequences of *Sobic.009g230200* are identical in the two cultivars. Fourth, knockdown or T-DNA insertion lines of orthologous genes in rice and Arabidopsis showed semi-dwarfism similar to that of the NIL-*dw1* sorghum (Supplementary Fig. S7; Fig. 3). Fifth, the complementation experiment showed that transformation of *Dw1* gene rescued dwarfing phenotype of the cultivar Tx430 having *dw1*.

How did the *dw1* mutation appear and spread? We also examined the genotypes of these SNPs in 3- and 4-dwarf (meaning that they carry three or four dwarf genes) cultivars, and confirmed that these cultivars contained the identical genome segment to ‘Dwarf White Milo’ carrying the causal SNP of *dw1* (Table 2), whereas, non-dwarf Kafir, Hegari, Broomcorn, and some other non-dwarf cultivars contained the same genome segment as SIL-05 (Table 2). These results coincide with the classic description that *dw1* mutation occurred only once in Memphis in 1905, which resulted in the appearance of Dwarf White Milo^12^. Subsequently, *dw1* on the genomic segment of Dwarf White Milo should have spread into various cultivars. It should be noted that the 4-dwarf Kafir also contains it in the Kafir genomic background. This demonstrates that the *dw1* mutation has been widely used not only for Milo, but also for Kafir cultivars. These results suggest this single allele has been important, precious, and valuable for the lodging resistance and mechanical harvesting during the history of sorghum breeding.

A synergistic interaction was observed between DW1 and DW3, which is involved in auxin transport. The pyramiding of dwarf genes should be useful in reducing sorghum to a suitable height, because sorghum is originally a tall plant that requires a significant height reduction without inducing detrimental side effects. In this situation, it might have been important to combine a few semi-dwarfing genes, which have no effects on any other traits but have synergistic or additive effects on height. The comparative study of the internode elongation pattern among NIL-*dw1,* NIL-*dw3,* and NIL-*dw1dw3* plants revealed that the effects of *dw1* and *dw3* are synergetic especially in the middle internodes IV–VII. (Fig. 4). Considering that many modern sorghum dwarf cultivars contain mutations in both *Dw1* and *Dw3*, the two genes have been selected in modern breeding as one of the ideal combinations. In this context, although DW1 and DW3 are interacting during the process of cell proliferation in the internode, they might function in two different molecular mechanisms or pathways. The finding of this novel factor, DW1 might be a good bridge to study not only breeding but also the interaction of auxin and dw1 -mediated mechanisms for cell proliferation in the internode.

## Methods

### Plant materials and growth conditions

The F_2_ plants of sorghum were grown under natural field conditions at the Faculty of Agriculture Education and Research Center of Alpine Field Science of Shinshu University (Ina, Japan) in 2008. Seeds were directly sown, and established one plant per hill with an 8 cm inter-hill spacing and 75 cm inter-row spacing. HIF plants were grown at the Togo Field Science and Education Center of Nagoya University (Aichi, Japan), and in the experimental field in Okinawa (Ohgimi, Japan) in 2010 and 2011. Seeds were sown in a nursery bed in a greenhouse, and 4-week-old seedlings were transplanted to the field with 15 cm spacing between each plant in two rows (30 cm in spacing) per hill (100 cm in width). The furrow was 80 cm in width. Genomic DNA was isolated from leaves of ~6-week-old field-grown plants using a cetyltrimethyl ammonium bromide extraction protocol^27^ with minor modifications. The SALK T-DNA insertion lines (SALK_038235 and SALK_059180) of Arabidopsis were obtained from the Arabidopsis Biological Resource Center (Columbus, OH, USA). Arabidopsis plants for mutants and WT were cultivated in a temperature-controlled chamber at 22 °C under continuous light (for plant height analysis).

### DNA simple-sequence repeat (SSR) markers

The SSR markers for sorghum, as reported by Brown *et al*.^28^, Bhattramakki *et al*.^29^, Kong *et al*.^30^, and Yonemaru *et al*.^31^ were screened, and SSR markers showing polymorphisms between the two parents were chosen. One SSR marker, SbYUC7, was additionally developed on chromosome 6. Thus, 162 SSR markers were used in this analysis.

### QTL analysis

A F_2_ population consisting of 185 individual plants derived from a cross between bmr-6 (a 3-dwarf short cultivar)^32^ and SIL-05 (a Japanese tall cultivar) was used for the distribution analysis of CL, and 96 plants were selected for the QTL analysis. The CL was measured from the ground to panicle node, and the flowering date was set as the number of days from sowing to flowering on the main panicle.

Linkage analyses and QTL identifications were performed using the MAPMAKER/QTL program^33^,^34^ based on genotypic data, while recombination frequencies were converted to genetic distances in centiMorgans (cM) using the Kosambi function^35^. The linkage map in Fig. 1 was created using Q-gene^36^. Primers used in this study are listed in Supplementary Table S1.

### Positional cloning of *Dw1*

HIFs were used in the positional cloning. For the generation of HIFs, one plant of each genotype, bmr-6 homozygous, SIL-05 homozygous, and bmr-6 heterozygous on *qCL-6*, *qCL-7*, and *qCL-9*, respectively, was selected from the F_3_ population, because the progeny plants were clearly segregated. The F_4_ and F_5_ were generated using the Single Seed Descent method, with a DNA marker selected from the same genotype as the parent. Thus, the progenies (F_5_ and F_6_), which are RILs containing a heterozygous fragment of *qCL-9*, were used to generate HIFs.

### RNA extraction, semi-quantitative RT-PCR analyses and RNA-seq

For semi-quantitative RT-PCR analysis, total RNAs were extracted from elongating internodes of sorghum at the vegetative stage (6 weeks after sowing) from SIL-05 and bmr-6 using TRIzol reagent (Invitrogen), or from leaves of transgenic rice using an RNeasy plant mini kit (Qiagen). The first-strand cDNA was synthesized using an Omniscript RT kit (Qiagen). The PCR parameters for the detection of *Sobic.009g229800, Sobic.009g229900, Sobic.009g230000, Sobic.009g231000,* and *Sobic.009g232000*. genes were 94 °C for 5 min followed by 35 cycles of 94 °C for 30 sec, 56 °C for 30 sec, and 72 °C for 30 sec. Sorghum *ubiquitin* (*Sobic.001G311100*; *SbUbi*) was used as the internal reference for all analyses. The parameters for *SbUbi, Os01g0103800*, *Os03g0270700*, and *OsActin* (*Os03g0718100*) genes were 27, 30, 30 and 25 cycles, respectively. The primers used for PCR are shown in Table S1. The extracted RNAs from the elongating internodes were subjected to RNA-seq experiments as described previously^37^. Primer sequences are listed in Supplementary Table S1.

### Phylogenetic tree analysis

DW1 protein homologs were screened using a BLAST search of Phytozome v.9.1 (http://www.phytozome.net/). The phylogenetic tree analysis and amino acid alignment were carried out using CLUSTALW^38^ under default settings and boxshade ver. 3.21 (http://www.ch.embnet.org/software/BOX_doc.html). Maximum likelihood tree based on the JTT model^39^ was obtained using Mega software. Bootstrap values were obtained by 1,000 bootstrap replicates.

### *Agrobacterium*-mediated rice transformation

To construct RNAi transgenic plants for simultaneous knockdowns of two genes, *Os01g0103800* and *Os03g0270700*, a common sequence in the two genes was amplified using PCR and fused using fusion PCR in both the sense and antisense directions (primers are listed in Supplementary Table S1). The RNAi construction is based on a previously described method^40^. The constructs were introduced into *A. tumefaciens* strain EHA105 and used to infect callus of rice cv. Taichung 65 as described by Ozawa^41^. Transformed cells and plants were selected using hygromycin resistance, and regenerants were grown to maturity in pots in a greenhouse. Transgenic plants of the T0 (for CL), and T_1_ or T_2_ (for seed length) generations were used for the analysis.

### *Agrobacterium*-mediated sorghum transformation

A binary vector with *bar* gene driven by maize *ubiquitin* promoter (*ubipro*), pBV*ubibar-Dw1* was constructed as follows. A digested 4.5 kb genomic DNA fragment containing *Sobic.009g229800* of SIL-05 was ligated in BamHI/PstI site of pCAMBIA1380 (Cambia) (pBV-*Dw1*). Next, a PCR amplified a DNA fragment of *bar* gene (without promoter) with *rbc* terminator sequence (*ter*) was swapped for *LUC* gene (without a *bar* promoter region) of pUC-LUC vector^42^ using in-fusion HD kit (Clontech). Finally, a DNA fragment of *ubipro*-*bar*-*ter* was amplified by PCR using the constructed DNA as a template, and swapped for the hygromycin resistance gene of pBV-*Dw1* by in-fusion HD kit. *Agrobacterium*-mediated transformation of sorghum cv. Tx430 was performed according to Wu *et al*.^43^ with a slight modification. Immature embryos of sorghum were inoculated with *A. tumefaciens* strain EHA105 harboring pBV*ubibar-Dw1*. Following a 2 days co-cultivation, the immature embryos were transferred to a resting medium and cultured at 25 °C in the dark for 12 days. For selection transformed cells, the immature embryos were subcultured on a selection medium containing phophinothricin (PPT) at 25 °C in the dark for 4 weeks. The selection pressure was gradually increased from 5 to 15 mg l^−1^ PPT. Obtained PPT-resistant calluses were cultured on a regeneration medium at 25 °C under continuous illumination (ca. 35 μmol m^−2^s^−1^) for 4 weeks and regenerated plantlets were cultured on a root induction medium for further 4 weeks. Both media contained 5 mg l^−1^ PPT. Well-rooted plants were transplanted to soil in pots and grown to maturity in a greenhouse. For DNA analysis, total genomic DNA was extracted from leaves using the E.Z.N.A. SP Plant DNA Kit (Omega Biotek).

### Microscopic analysis

Culm longitudinal slices (110-μm in thickness) of the NILs were prepared using a vibrating microslicer (D.S.K DTK-3000), followed by toluidine blue staining. Photographs were taken using an Olympus stereoscopic microscope connected to a CCD camera (Olympus DP20). To quantify the length and number of the cells in parenchyma, we measured them in five cell files within a 2.1-mm long region of the ninth internode and then estimated the total number of cells in the internode.

## Additional Information

**How to cite this article**: Yamaguchi, M. *et al*. Sorghum *Dw1*, an agronomically important gene for lodging resistance, encodes a novel protein involved in cell proliferation. *Sci. Rep.* **6**, 28366; doi: 10.1038/srep28366 (2016).

## Supplementary Material

Supplementary Information

## Figures and Tables

**Table 1:** QTLs for CL identified in the F_2_ population of a crossing between bmr-6 and SIL-05.

QTL	Chr.	Nearest markers	LOD^a^	PVE^b^	a^c^	DOM^d^	d/a	DPE^e^
*qCL-6*	6	SB3484	6.09	26.0	60.7	37.9	0.62	SIL-05
*qCL-7*	7	SSR3_4143	7.01	35.8	60.2	69.7	1.16	SIL-05
*qCL-9*	9	SSR2_5041	3.77	17.6	45.2	29.7	0.66	SIL-05

^a^LOD threshold = 3.0.

^b^Percentage of Variance-explained; Percent of total phenotypic variation explained by the QTLs.

^c^Additive effects are associated with the SIL-05 allele.

^d^Dominance effect in the Het.

^e^Direction of phenotypic effect is the parent whose additive value of a marker allele increased the trait value.

**Table 2:** The graphical view of haplotypes around *Dw1* among cultivars.

	genotype^b^	Position (Mb)^a^						
		57.208	57.211^c^	57.229	57.240	57.266	57.2721	57.2729
Parental lines^d^			(*dw1*)					
SIL-05		*S*	*S*	*S*	*S*	*S*	*S*	*S*
bmr-6		**b**	**b**	**b**	**b**	**b**	**b**	**b**
			↓*dw1* emergence					
Original milo (1-dwarf cultivars)^e^								
Tall white Sooner Milo (SA1170)	*Dw1*	**b**	*S*	**b**	**b**	**b**	**b**	**b**
Standard Milo		**b**	*S*	**b**	**b**	**b**	**b**	**b**
			↓used for breeding					
2-dwarf cultivars^f^								
Dwarf White Milo	*dw1*	**b**	**b**	**b**	**b**	**b**	**b**	**b**
3-dwarf cultivars^g^								
D.D. Yellow Sooner Milo	*dw1*	**b**	**b**	**b**	**b**	**b**	**b**	**b**
Martin 3dw	*dw1*	**b**	**b**	**b**	**b**	**b**	**b**	**b**
Plainsman 3dw	*dw1*	**b**	**b**	**b**	**b**	**b**	**b**	**b**
38 M		**b**	**b**	**b**	**b**	**b**	**b**	**b**
44 M		**b**	**b**	**b**	**b**	**b**	**b**	**b**
58 M		**b**	**b**	**b**	**b**	**b**	**b**	**b**
60 M		**b**	**b**	**b**	**b**	**b**	**b**	**b**
80 M		**b**	**b**	**b**	**b**	**b**	**b**	**b**
90 M		**b**	**b**	**b**	**b**	**b**	**b**	**b**
100 M		**b**	**b**	**b**	**b**	**b**	**b**	**b**
SM60		**b**	**b**	**b**	**b**	**b**	**b**	**b**
SM80		**b**	**b**	**b**	**b**	**b**	**b**	**b**
SM90		**b**	**b**	**b**	**b**	**b**	**b**	**b**
SM100		**b**	**b**	**b**	**b**	**b**	**b**	**b**
ATx 623	*dw1*	**b**	**b**	**b**	**b**	**b**	**b**	**b**
BTx 623	*dw1*	**b**	**b**	**b**	**b**	**b**	**b**	**b**
4-dwarf cultivars^g^								
3121A 4dw Martin	*dw1*	**b**	**b**	**b**	**b**	**b**	**b**	**b**
3127A 4dw Kafir	*dw1*	**b**	**b**	**b**	**b**	**b**	**b**	**b**
4dw 380B		**b**	**b**	**b**	**b**	**b**	**b**	**b**
Other cultivars^h^								
Texas Blackhull Kafir		*S*	*S*	*S*	*S*	*S*	*S*	*S*
Pink Kafir		*S*	*S*	*S*	*S*	*S*	*S*	*S*
Hegari		*S*	*S*	*S*	*S*	*S*	*S*	*S*
Early Hegari		*S*	*S*	*S*	*S*	*S*	*S*	*S*
Black Spanish Standard Broom Corn		*S*	*S*	*S*	*S*	*S*	*S*	*S*
Japanese Dwarf Standard Broom Corn		*S*	*S*	*S*	*S*	*S*	*S*	*S*
Sumac		*S*	*S*	*S*	*S*	*S*	*S*	*S*
Bonita		*S*	*S*	*S*	*S*	*S*	*S*	*S*

^a^The physical positions of seven SNPs, which were also presented as arrowheads on the top line in Fig. 2a.

^b^The genotype of *Dw1* reported by Quinby *et al*.^21^.

^c^The SNP in *Dw1* that forms the premature stop codon in bmr-6 (b highlighted with bold font), while SIL-05 contains its WT allele (S highlighted with italics).

^d^SNPs of SIL-05 and bmr-05 presented as S and b, respectively (highlighted with italic and bold font, respectively).

^e^Original milo such as ‘Tall White Sooner Milo’ (SA1170) carrying *Dw1*, and Standard milo contain the same haplotype of ‘Dwarf White Milo’ with one exception, the SNP in *Dw1.*

^f^The 2-dwarf type ‘Dwarf White Milo’ carrying *dw1* contains the same haplotype as bmr-6.

^g^All 3- or 4-dwarf cultivars contain the same haplotype as bmr-6 or ‘Dwarf White Milo’.

^h^Other cultivars, including Kafir and Hegari, contain the same haplotype as SIL-05.
