# Supplementary material for: Sorghum Dw1, an agronomically important gene for lodging resistance, encodes a novel protein involved in cell proliferation
Source: Sci Rep. 2016 Jun 22;6:28366. doi: 10.1038/srep28366 (PMC4916599; doi:10.1038/srep28366)
Supplement: Supplementary Information [file srep28366-s1.pdf]

Title: **Sorghum *Dw1*, an agronomically important gene for lodging resistance, encodes a novel protein involved in cell proliferation**

Authors: Miki Yamaguchi[1], Haruka Fujimoto[1], Ko Hirano[1], Satoko Araki-Nakamura[1], Kozue Ohmae-Shinohara[1], Akihiro Fujii[1], Masako Tsunashima[2], Xian Jun Song[1†], Yusuke Ito[1], Rie Nagae[1], Jianzhong Wu[3], Hiroshi Mizuno[3], Jun-ichi Yonemaru[3], Takashi Matsumoto[3], Hidemi Kitano[1], Makoto Matsuoka[1], Shigemitsu Kasuga[4], Takashi Sazuka[1*]

Affiliations:

[1]Bioscience and Biotechnology Center, Nagoya University, Furou-cho, Nagoya, Aichi 464-8601, Japan

[2]Plant Innovation Center, Japan Tobacco Inc., Iwata, Shizuoka 438–0802, Japan

[3]National Institute of Agrobiological Sciences, Kannondai, Tsukuba, Ibaraki 305-8602, Japan

[4]Education and Research Center of Alpine Field Science, Faculty of Agriculture, Shinshu University, Minamiminowa, Nagano 399-4598, Japan

[†]Present address: Key Laboratory of Plant Molecular Physiology, Institute of Botany, the Chinese Academy of Sciences, Beijing 100093, China.

*Correspondence to:
Takashi Sazuka
Bioscience and Biotechnology Center, Nagoya University
Chikusa, Nagoya 464-8601, Japan
Tel: +81-52-789-5217
Fax: +81-52-789-5226
E-mail: sazuka@agr.nagoya-u.ac.jp

Supplementary figures: S1 to S7
Supplementary tables: S1

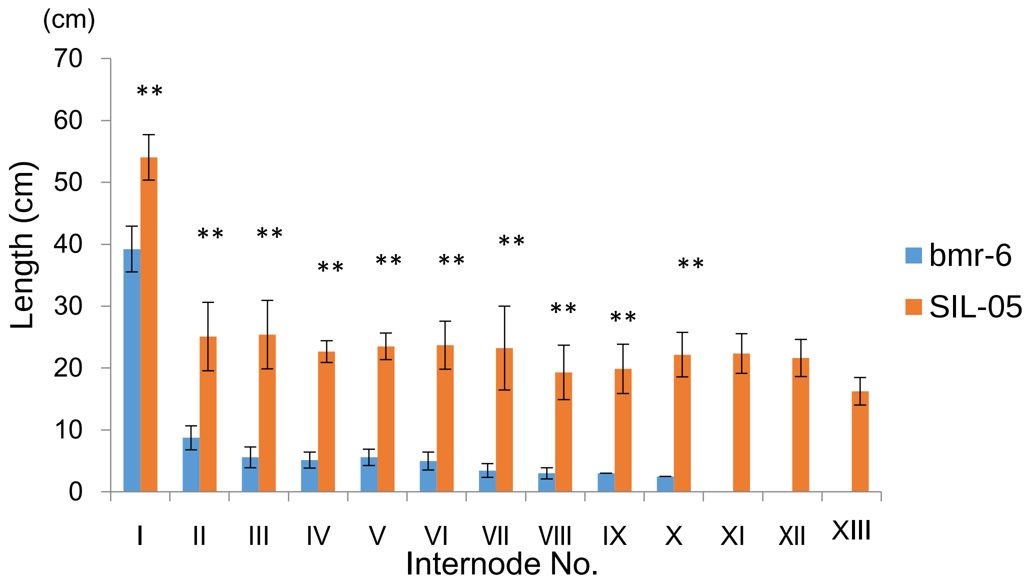

Supplementary Figure S1 | Length of each internode of bmr-6 (blue) and SIL-05 (orange). Two asterisks indicates $P < 0.01$.

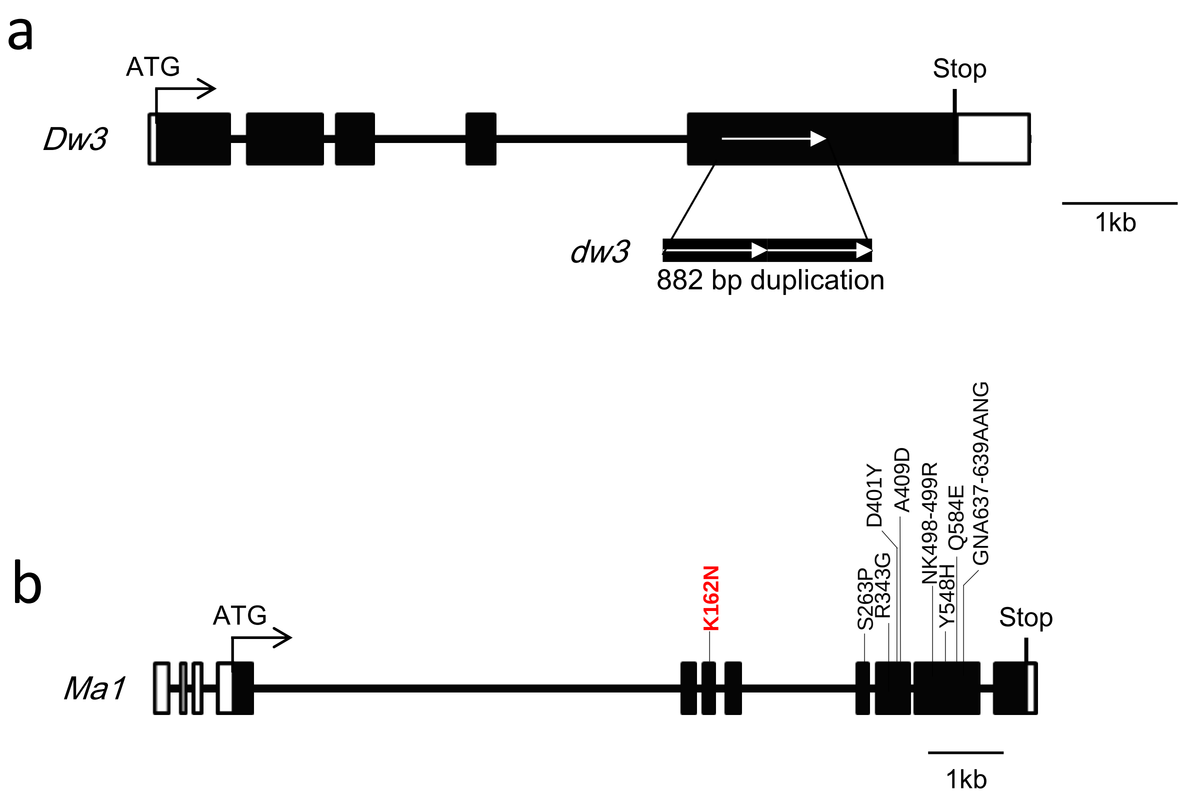

Supplementary Figure S2 | bmr-6 contains the loss-of-function alleles of *Dw3* and *Ma1*. The gene structures of *Dw3* (a) and *Ma1* (b), and their mutations are shown. Protein coding regions and UTRs are represented by black and white boxes, respectively. Introns are indicated by black bars. 'ATG' and 'Stop' indicate the translation initiation and stop codons, respectively. (a) bmr-6 contains a duplication of 882 bp in *Dw3*. (b) Positions of nine amino acid exchanges are shown, and the mutation K162N, which was reported (see text) is indicated in bold red.

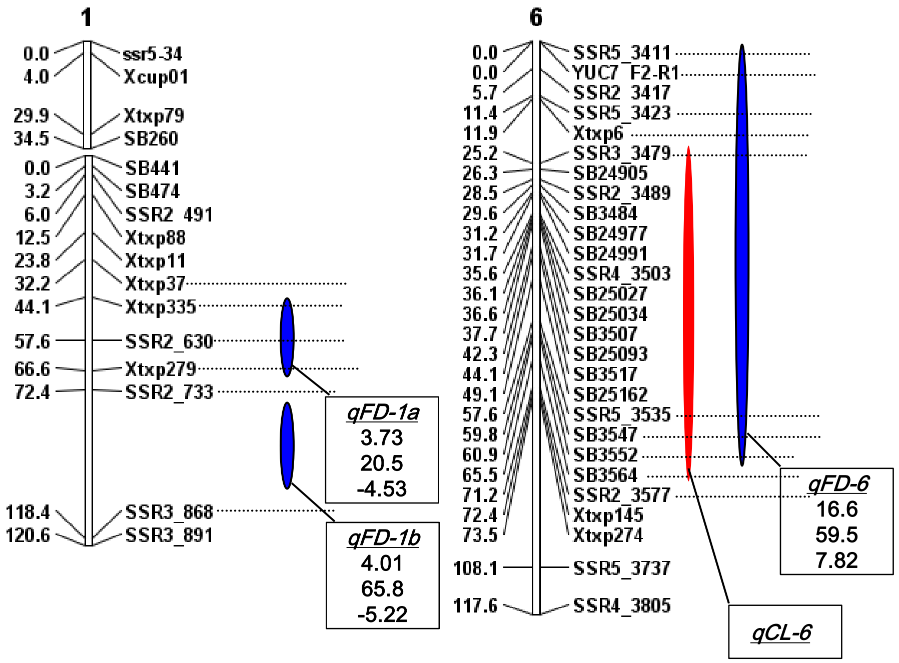

Supplementary Figure S3 | QTL analysis for FD and a comparison of QTL map positions for FD and CL. Blue and red ellipses indicate the positions of QTLs for FD and CL, respectively. Only chromosomes with LOD scores > 3.0 are shown. Information in the box is same as in Fig. 1e. The SIL-05 allele delayed the FD in *qFD-6*, and the bmr-6 allele accelerated the FD in *qFD-1a* and *qFD-1b*.

```
BTx623                        1:CATACGTGTTTTACTTTTACACTATTGGTGGTGGCTTGACTCAACTGCTCAGTGCTCAGG 60
Dwarf white milo              1:CATACGTGTTTTACTTTTACACTATTGGTGGTGGCTTGACTCAACTGCTCAGTGCTCAGG 60
Tall white sooner milo        1:CATACGTGTTTTACTTTTACACTATTGGTGGTGGCTTGACTCAACTGCTCAGTGCTCAGG 60
bmr-6                         1:CATACGTGTTTTACTTTTACACTATTGGTGGTGGCTTGACTCAACTGCTCAGTGCTCAGG 60
                                ************************************************************

BTx623                       61:CACCTTGCTCGCCCGCGCGCAGCACGGCAGCACGCAAGCGACGGCGCGACGCTCCACCGC 120
Dwarf white milo             61:CACCTTGCTCGCCCGCGCGCAGCACGGCAGCACGCAAGCGACGGCGCGACGCTCCACCGC 120
Tall white sooner milo       61:CACCTTGCTCGCCCGCGCGCAGCACGGCAGCACGCAAGCGACGGCGCGACGCTCCACCGC 120
bmr-6                        61:CACCTTGCTCGCCCGCGCGCAGCACGGCAGCACGCAAGCGACGGCGCGACGCTCCACCGC 120
                                ************************************************************

BTx623                      121:TCTCCGCTCGATCCCTCTCTTCTCCCATTTCGCAGGCGACGGCGCGACGCTCCACCGCAC 180
Dwarf white milo            121:TCTCCGCTCGATCCCTCTCTTCTCCCATTTCGCAGGCGACGGCGCGACGCTCCACCGCAC 180
Tall white sooner milo      121:TCTCCGCTCGATCCCTCTCTTCTCCCATTTCGCAGGCGACGGCGCGACGCTCCACCGCAC 180
bmr-6                       121:TCTCCGCTCGATCCCTCTCTTCTCCCATTTCGCAGGCGACGGCGCGACGCTCCACCGCAC 180
                                ************************************************************

BTx623                      181:CGCGAGGCAAGGTCCCTCTTCGTTGAGCTCGCTGCTCGGGGTTCGGGGCGGGTTTGAGTA 240
Dwarf white milo            181:CGCGAGGCAAGGTCCCTCTTCGTTGAGCTCGCTGCTCGGGGTTCGGGGCGGGTTTGAGTA 240
Tall white sooner milo      181:CGCGAGGCAAGGTCCCTCTTCGTTGAGCTCGCTGCTCGGGGTTCGGGGCGGGTTTGAGTA 240
bmr-6                       181:CGCGAGGCAAGGTCCCTCTTCGTTGAGCTCGCTGCTCGGGGTTCGGGGCGGGTTTGAGTA 240
                                ************************************************************

BTx623                      241:TCTGATTTCGGGTGCGGGTACGCGGATGCTCCACCCGCCCCCGACCCGCTCCGTTGCCAT 300
Dwarf white milo            241:TCTGATTTCGGGTGCGGGTACGCGGATGCTCCACCCGCCCCCGACCCGCTCCGTTGCCAT 300
Tall white sooner milo      241:TCTGATTTCGGGTGCGGGTACGCGGATGCTCCACCCGCCCCCGACCCGCTCCGTTGCCAT 300
bmr-6                       241:TCTGATTTCGGGTGCGGGTACGCGGATGCTCCACCCGCCCCCGACCCGCTCCGTTGCCAT 300
                                ************************************************************

BTx623                      301:CCCGTGCCGTGCTCACCCCCCTACCTTTTCTTCTCCGGTTCACCACCCCCTCGTGCGCTC 360
Dwarf white milo            301:CCCGTGCCGTGCTCACCCCCCTACCTTTTCTTCTCCGGTTCACCACCCCCTCGTGCGCTC 360
Tall white sooner milo      301:CCCGTGCCGTGCTCACCCCCCTACCTTTTCTTCTCCGGTTCACCACCCCCTCGTGCGCTC 360
bmr-6                       301:CCCGTGCCGTGCTCACCCCCCTACCTTTTCTTCTCCGGTTCACCACCCCCTCGTGCGCTC 360
                                ************************************************************

BTx623                      361:TCCTCACTCTCACTCAGCTCTCTCTTTCTCTCTCGTGGGAGACGACTGGGCGACCGCCGG 420
Dwarf white milo            361:TCCTCACTCTCACTCAGCTCTCTCTTTCTCTCTCGTGGGAGACGACTGGGCGACCGCCGG 420
Tall white sooner milo      361:TCCTCACTCTCACTCAGCTCTCTCTTTCTCTCTCGTGGGAGACGACTGGGCGACCGCCGG 420
bmr-6                       361:TCCTCACTCTCACTCAGCTCTCTCTTTCTCTCTCGTGGGAGACGACTGGGCGACCGCCGG 420
                                ************************************************************

BTx623                      421:CGCCGACCGTGCTCAGGCCTTCGTCGAGACCATCGCCGGCGAAGAGCACCCACCAGGTAT 480
Dwarf white milo            421:CGCCGACCGTGCTCAGGCCTTCGTCGAGACCATCGCCGGCGAAGAGCACCCACCAGGTAT 480
Tall white sooner milo      421:CGCCGACCGTGCTCAGGCCTTCGTCGAGACCATCGCCGGCGAAGAGCACCCACCAGGTAT 480
bmr-6                       421:CGCCGACCGTGCTCAGGCCTTCGTCGAGACCATCGCCGGCGAAGAGCACCCACCAGGTAT 480
                                ************************************************************

BTx623                      481:GCGCACTTCTTGTCTTCCCTTCCAGATGCGCGAAACCGAGCACGCAAACCCTAACTTAAG 540
Dwarf white milo            481:GCGCACTTCTTGTCTTCCCTTCCAGATGCGCGAAACCGAGCACGCAAACCCTAACTTAAG 540
Tall white sooner milo      481:GCGCACTTCTTGTCTTCCCTTCCAGATGCGCGAAACCGAGCACGCAAACCCTAACTTAAG 540
bmr-6                       481:GCGCACTTCTTGTCTTCCCTTCCAGATGCGCGAAACCGAGCACGCAAACCCTAACTTAAG 540
                                ************************************************************

BTx623                      541:CTATTTGGCTATTTGCAGCTGGATCTGATCTAATTACAGGTGCATACGCGCATTATGCCT 600
Dwarf white milo            541:CTATTTGGCTATTTGCAGCTGGATCTGATCTAATTACAGGTGCATACGCGCATTATGCCT 600
Tall white sooner milo      541:CTATTTGGCTATTTGCAGCTGGATCTGATCTAATTACAGGTGCATACGCGCATTATGCCT 600
bmr-6                       541:CTATTTGGCTATTTGCAGCTGGATCTGATCTAATTACAGGTGCATACGCGCATTATGCCT 600
                                ************************************************************

BTx623                      601:ACTAATTTCGATTCCTTGCAAAAATTGTCGGCTGTATTTGTGTACACCCATGCCATTTAC 660
Dwarf white milo            601:ACTAATTTCGATTCCTTGCAAAAATTGTCGGCTGTATTTGTGTACACCCATGCCATTTAC 660
Tall white sooner milo      601:ACTAATTTCGATTCCTTGCAAAAATTGTCGGCTGTATTTGTGTACACCCATGCCATTTAC 660
bmr-6                       601:ACTAATTTCGATTCCTTGCAAAAATTGTCGGCTGTATTTGTGTACACCCATGCCATTTAC 660
                                ************************************************************

BTx623                      661:TGGGTCCGCCTCTGATCCCGAGCAACCGTGTGTGTTTACAGTCAGAGATGTCTTCAGTTG 720
Dwarf white milo            661:TGGGTCCGCCTCTGATCCCGAGCAACCGTGTGTGTTTACAGTCAGAGATGTCTTCAGTTG 720
Tall white sooner milo      661:TGGGTCCGCCTCTGATCCCGAGCAACCGTGTGTGTTTACAGTCAGAGATGTCTTCAGTTG 720
bmr-6                       661:TGGGTCCGCCTCTGATCCCGAGCAACCGTGTGTGTTTACAGTCAGAGATGTCTTCAGTTG 720
                                ************************************************************
```

Figure S4 (page1)

```
BTx623                  721:GGAGCAGCCCTGGCACCAGGGCGGCGAATGGTGCCGCTGCCATCAGTGCTGCCGCGACGG 780
Dwarf white milo        721:GGAGCAGCCCTGGCACCAGGGCGGCGAATGGTGCCGCTGCCATCAGTGCTGCCGCGACGG 780
Tall white sooner milo  721:GGAGCAGCCCTGGCACCAGGGCGGCGAATGGTGCCGCTGCCATCAGTGCTGCCGCGACGG 780
bmr-6                   721:GGAGCAGCCCTGGCACCAGGGCGGCGAATGGTGCCGCTGCCATCAGTGCTGCCGCGACGG 780
                            ************************************************************

BTx623                  781:CAGCAGGCTCAGCCGACGCCAGATTCCACTCCCAACTACTACAGCAGGACAGGGTACATA 840
Dwarf white milo        781:CAGCAGGCTCAGCCGACGCCAGATTCCACTCCCAACTACTACAGCAGGACAGGGTACATA 840
Tall white sooner milo  781:CAGCAGGCTCAGCCGACGCCAGATTCCACTCCCAACTACTACAGCAGGACAGGGTACATA 840
bmr-6                   781:CAGCAGGCTCAGCCGACGCCAGATTCCACTCCCAACTACTACAGCAGGACAGGGTACATA 840
                            ************************************************************

BTx623                  841:CACTATATACCACTTGTTGTTCCATGGGCGGATGCAATCGGGCTTGGGCTTTAGCTGCAG 900
Dwarf white milo        841:CACTATATACCACTTGTTGTTCCATGGGCGGATGCAATCGGGCTTGGGCTTTAGCTGCAG 900
Tall white sooner milo  841:CACTATATACCACTTGTTGTTCCATGGGCGGATGCAATCGGGCTTGGGCTTTAGCTGCAG 900
bmr-6                   841:CACTATATACCACTTGTTGTTCCATGGGCGGATGCAATCGGGCTTGGGCTTTAGCTGCAG 900
                            ************************************************************

BTx623                  901:CTTTTGCCTTGCATGCTTTAGATATGTGGACGACAGGATCAGTACCGGAATTTTTAGTCG 960
Dwarf white milo        901:CTTTTGCCTTGCATGCTTTAGATATGTGGACGACAGGATCAGTACCGGAATTTTTAGTCG 960
Tall white sooner milo  901:CTTTTGCCTTGCATGCTTTAGATATGTGGACGACAGGATCAGTACCGGAATTTTTAGTCG 960
bmr-6                   901:CTTTTGCCTTGCATGCTTTAGATATGTGGACGACAGGATCAGTACCGGAATTTTTAGTCG 960
                            ************************************************************

BTx623                  961:GCCTTGACGACCTAGATTGGCAATTTGGGCCAGCCTTTGCTTACTCAGTACGAATTGTAC 1020
Dwarf white milo        961:GCCTTGACGACCTAGATTGGCAATTTGGGCCAGCCTTTGCTTACTCAGTACGAATTGTAC 1020
Tall white sooner milo  961:GCCTTGACGACCTAGATTGGCAATTTGGGCCAGCCTTTGCTTACTCAGTACGAATTGTAC 1020
bmr-6                   961:GCCTTGACGACCTAGATTGGCAATTTGGGCCAGCCTTTGCTTACTCAGTACGAATTGTAC 1020
                            ************************************************************

BTx623                 1021:TGCCCTGCAGTGGTTATGACTTGAAACTGGGCATCTTTACCAGCAATAATTTGGCTTCAG 1080
Dwarf white milo       1021:TGCCCTGCAGTGGTTATGACTTGAAACTGGGCATCTTTACCAGCAATAATTTGGCTTCAG 1080
Tall white sooner milo 1021:TGCCCTGCAGTGGTTATGACTTGAAACTGGGCATCTTTACCAGCAATAATTTGGCTTCAG 1080
bmr-6                  1021:TGCCCTGCAGTGGTTATGACTTGAAACTGGGCATCTTTACCAGCAATAATTTGGCTTCAG 1080
                            ************************************************************

BTx623                 1081:TACTATATATATGCTTGGTTGCTTCTGTCAGATAATTTATTTAGTTGGATTTTGCTTTTG 1140
Dwarf white milo       1081:TACTATATATATGCTTGGTTGCTTCTGTCAGATAATTTATTTAGTTGGATTTTGCTTTTG 1140
Tall white sooner milo 1081:TACTATATATATGCTTGGTTGCTTCTGTCAGATAATTTATTTAGTTGGATTTTGCTTTTG 1140
bmr-6                  1081:TACTATATATATGCTTGGTTGCTTCTGTCAGATAATTTATTTAGTTGGATTTTGCTTTTG 1140
                            ************************************************************

BTx623                 1141:CTATGTTGTAATGCACACGTTTTGCACTCCTTATTTTGTTTTCAGTTCCAGAGGCTTTTG 1200
Dwarf white milo       1141:CTATGTTGTAATGCACACGTTTTGCACTCCTTATTTTGTTTTCAGTTCCAGAGGCTTTTG 1200
Tall white sooner milo 1141:CTATGTTGTAATGCACACGTTTTGCACTCCTTATTTTGTTTTCAGTTCCAGAGGCTTTTG 1200
bmr-6                  1141:CTATGTTGTAATGCACACGTTTTGCACTCCTTATTTTGTTTTCAGTTCCAGAGGCTTTTG 1200
                            ************************************************************

BTx623                 1201:TGATATCATTCCTCCAGAGAATGTATATACTAATAAGTTCTTGATTTTACCCCTCGATAC 1260
Dwarf white milo       1201:TGATATCATTCCTCCAGAGAATGTATATACTAATAAGTTCTTGATTTTACCCCTCGATAC 1260
Tall white sooner milo 1201:TGATATCATTCCTCCAGAGAATGTATATACTAATAAGTTCTTGATTTTACCCCTCGATAC 1260
bmr-6                  1201:TGATATCATTCCTCCAGAGAATGTATATACTAATAAGTTCTTGATTTTACCCCTCGATAC 1260
                            ************************************************************

BTx623                 1261:ACTCCCACCCATTTGATGTTGAAGTAGGGGCAAAAAGCTTTTACACCTGGGATAATCCTT 1320
Dwarf white milo       1261:ACTCCCACCCATTTGATGTTGAAGTAGGGGCAAAAAGCTTTTACACCTGGGATAATCCTT 1320
Tall white sooner milo 1261:ACTCCCACCCATTTGATGTTGAAGTAGGGGCAAAAAGCTTTTACACCTGGGATAATCCTT 1320
bmr-6                  1261:ACTCCCACCCATTTGATGTTGAAGTAGGGGCAAAAAGCTTTTACACCTGGGATAATCCTT 1320
                            ************************************************************

BTx623                 1321:GCTTTATGATAAAAATCTTTTTCCATGTGAATACTTTTGGATTTTTGTGAAACACTAACA 1380
Dwarf white milo       1321:GCTTTATGATAAAAATCTTTTTCCATGTGAATACTTTTGGATTTTTGTGAAACACTAACA 1380
Tall white sooner milo 1321:GCTTTATGATAAAAATCTTTTTCCATGTGAATACTTTTGGATTTTTGTGAAACACTAACA 1380
bmr-6                  1321:GCTTTATGATAAAAATCTTTTTCCATGTGAATACTTTTGGATTTTTGTGAAACACTAACA 1380
                            ************************************************************

BTx623                 1381:TTTCGTATGTTCTATTGTATGATTCCTTTGATGCTTATTCTTATGCATTTTTTCTTCCCA 1440
Dwarf white milo       1381:TTTCGTATGTTCTATTGTATGATTCCTTTGATGCTTATTCTTATGCATTTTTTCTTCCCA 1440
Tall white sooner milo 1381:TTTCGTATGTTCTATTGTATGATTCCTTTGATGCTTATTCTTATGCATTTTTTCTTCCCA 1440
bmr-6                  1381:TTTCGTATGTTCTATTGTATGATTCCTTTGATGCTTATTCTTATGCATTTTTTCTTCCCA 1440
                            ************************************************************
```

Figure S4 (page2)

```
BTx623                  1441:CAACACAATGATAATAAAAATGTTTTTTTGTATACAAAGTATAGATTACTGCTCCTTTGA 1500
Dwarf white milo        1441:CAACACAATGATAATAAAAATGTTTTTTTGTATACAAAGTATAGATTACTGCTCCTTTGA 1500
Tall white sooner milo  1441:CAACACAATGATAATAAAAATGTTTTTTTGTATACAAAGTATAGATTACTGCTCCTTTGA 1500
bmr-6                   1441:CAACACAATGATAATAAAAATGTTTTTTTGTATACAAAGTATAGATTACTGCTCCTTTGA 1500
                             ************************************************************

BTx623                  1501:AATTTCGTTGTCATGTGTTTTCTTTTATTTCTGTGGTGACAGCTAAGTTATTTGCTTGGA 1560
Dwarf white milo        1501:AATTTCGTTGTCATGTGTTTTCTTTTATTTCTGTGGTGACAGCTAAGTTATTTGCTTGGA 1560
Tall white sooner milo  1501:AATTTCGTTGTCATGTGTTTTCTTTTATTTCTGTGGTGACAGCTAAGTTATTTGCTTGGA 1560
bmr-6                   1501:AATTTCGTTGTCATGTGTTTTCTTTTATTTCTGTGGTGACAGCTAAGTTATTTGCTTGGA 1560
                             ************************************************************

BTx623                  1561:CTAACACACGCTTCTCTACTCTAAACAGCAAAGTAGATGGGCTGGCTGCTTTTCAGGTTT 1620
Dwarf white milo        1561:CTAACACACGCTTCTCTACTCTAAACAGCAAAGTAGATGGGCTGGCTGCTTTTCAGGTTT 1620
Tall white sooner milo  1561:CTAACACACGCTTCTCTACTCTAAACAGCAAAGTAGATGGGCTGGCTGCTTTTCAGGTTT 1620
bmr-6                   1561:CTAACACACGCTTCTCTACTCTAAACAGCAAAGTAGATGGGCTGGCTGCTTTTCAGGTTT 1620
                             ************************************************************

BTx623                  1621:ATCATGTTTCGGATCGCAGAAGGGCGGGAAAAGAATTGTTCCTGCAGCACGTACTTCTGA 1680
Dwarf white milo        1621:ATCATGTTTCGGATCGCAGAAGGGCGGGAAAAGAATTGTTCCTGCAGCACGTACTTCTGA 1680
Tall white sooner milo  1621:ATCATGTTTCGGATCGCAGAAGGGCGGGAAAAGAATTGTTCCTGCAGCACGTACTTCTGA 1680
bmr-6                   1621:ATCATGTTTCGGATCGCAGAAGGGCGGGAAAAGAATTGTTCCTGCAGCACGTACTTCTGA 1680
                             ************************************************************

BTx623                  1681:TGGGAATGGATCAAATGCTCGTGGAAATGGTCAATCTGGTGCCAATTCAAACCAAAATAT 1740
Dwarf white milo        1681:TGGGAATGGATCAAATGCTCGTGGAAATGGTCAATCTGGTGCCAATTCAAACCAAAATAT 1740
Tall white sooner milo  1681:TGGGAATGGATCAAATGCTCGTGGAAATGGTCAATCTGGTGCCAATTCAAACCAAAATAT 1740
bmr-6                   1681:TGGGAATGGATCAAATGCTCGTGGAAATGGTCAATCTGGTGCCAATTCAAACCAAAATAT 1740
                             ************************************************************

BTx623                  1741:GCCTATGAATTTATCTCTTCTGGCTCCACCATCATCACCAGCATCCTTCTCAAACTCTGC 1800
Dwarf white milo        1741:GCCTATGAATTTATCTCTTCTGGCTCCACCATCATCACCAGCATCCTTCTCAAACTCTGC 1800
Tall white sooner milo  1741:GCCTATGAATTTATCTCTTCTGGCTCCACCATCATCACCAGCATCCTTCTCAAACTCTGC 1800
bmr-6                   1741:GCCTATGAATTTATCTCTTCTGGCTCCACCATCATCACCAGCATCCTTCTCAAACTCTGC 1800
                             ************************************************************

BTx623                  1801:CCTTCCTTCGACTGCTCAATCACCGAATTGCTTTCTGTCTGTATCTGCAAACTCTCCTGG 1860
Dwarf white milo        1801:CCTTCCTTCGACTGCTCAATCACCGAATTGCTTTCTGTCTGTATCTGCAAACTCTCCTGG 1860
Tall white sooner milo  1801:CCTTCCTTCGACTGCTCAATCACCGAATTGCTTTCTGTCTGTATCTGCAAACTCTCCTGG 1860
bmr-6                   1801:CCTTCCTTCGACTGCTCAATCACCGAATTGCTTTCTGTCTGTATCTGCAAACTCTCCTGG 1860
                             ************************************************************

BTx623                  1861:CGGTCCAACGTCTAATATGTTCGCTGTTGGGCCATATGCTAATGAACCTCAACTTGTCTC 1920
Dwarf white milo        1861:CGGTCCAACGTCTAATATGTTCGCTGTTGGGCCATATGCTAATGAACCTCAACTTGTCTC 1920
Tall white sooner milo  1861:CGGTCCAACGTCTAATATGTTCGCTGTTGGGCCATATGCTAATGAACCTCAACTTGTCTC 1920
bmr-6                   1861:CGGTCCAACGTCTAATATGTTCGCTGTTGGGCCATATGCTAATGAACCTCAACTTGTCTC 1920
                             ************************************************************

BTx623                  1921:GCCACCTGTCTTCTCAACTTACACAACTGAGCCATCCACAGCACCATTGACCCCACCACC 1980
Dwarf white milo        1921:GCCACCTGTCTTCTCAACTTACACAACTGAGCCATCCACAGCACCATTGACCCCACCACC 1980
Tall white sooner milo  1921:GCCACCTGTCTTCTCAACTTACACAACTGAGCCATCCACAGCACCATTGACCCCACCACC 1980
bmr-6                   1921:GCCACCTGTCTTCTCAACTTACACAACTGAGCCATCCACAGCACCATTGACCCCACCACC 1980
                             ************************************************************

BTx623                  1981:TGAACTAGCTCATGCAACAACGCCTTCTTCTCCAGATGTTCCATATGCTCGATTTCTTTC 2040
Dwarf white milo        1981:TGAACTAGCTCATGCAACAACGCCTTCTTCTCCAGATGTTCCATATGCTCGATTTCTTTC 2040
Tall white sooner milo  1981:TGAACTAGCTCATGCAACAACGCCTTCTTCTCCAGATGTTCCATATGCTCGATTTCTTTC 2040
bmr-6                   1981:TGAACTAGCTCATGCAACAACGCCTTCTTCTCCAGATGTTCCATATGCTCGATTTCTTTC 2040
                             ************************************************************
                                              ▼
BTx623                  2041:TTCTTCTATGGATATCTAAACTGCCAGCAAGGAGCATAACATGCCTTTCTTATCAACAGC 2100
Dwarf white milo        2041:TTCTTCTATGGATATCTAAACTGCCAGCAAGGAGCATAACATGCCTTTCTTATCAACAGC 2100
Tall white sooner milo  2041:TTCTTCTATGGATATCAAAACTGCCAGCAAGGAGCATAACATGCCTTTCTTATCAACAGC 2100
bmr-6                   2041:TTCTTCTATGGATATCTAAACTGCCAGCAAGGAGCATAACATGCCTTTCTTATCAACAGC 2100
                             ****************.*******************************************

BTx623                  2101:CTATTCTGGTGGTTCAGGACTCCAGGCATCCTACCCACTTTACCCTGAAAGCCCTTGTAG 2160
Dwarf white milo        2101:CTATTCTGGTGGTTCAGGACTCCAGGCATCCTACCCACTTTACCCTGAAAGCCCTTGTAG 2160
Tall white sooner milo  2101:CTATTCTGGTGGTTCAGGACTCCAGGCATCCTACCCACTTTACCCTGAAAGCCCTTGTAG 2160
bmr-6                   2101:CTATTCTGGTGGTTCAGGACTCCAGGCATCCTACCCACTTTACCCTGAAAGCCCTTGTAG 2160
                             ************************************************************
```

Figure S4 (page3)

```
BTx623                  2161:CAGCCTCATCTCACCAGCTTCAGTAACTCCGAGGACTGGTCTATCCTCACCTATACCTGA 2220
Dwarf white milo        2161:CAGCCTCATCTCACCAGCTTCAGTAACTCCGAGGACTGGTCTATCCTCACCTATACCTGA 2220
Tall white sooner milo  2161:CAGCCTCATCTCACCAGCTTCAGTAACTCCGAGGACTGGTCTATCCTCACCTATACCTGA 2220
bmr-6                   2161:CAGCCTCATCTCACCAGCTTCAGTAACTCCGAGGACTGGTCTATCCTCACCTATACCTGA 2220
                             ************************************************************

BTx623                  2221:GCAAGAGGTTCCTCCTGCCCATTGGAAGACCTCTAGGTCCGCCTGCGACACGCCATACTT 2280
Dwarf white milo        2221:GCAAGAGGTTCCTCCTGCCCATTGGAAGACCTCTAGGTCCGCCTGCGACACGCCATACTT 2280
Tall white sooner milo  2221:GCAAGAGGTTCCTCCTGCCCATTGGAAGACCTCTAGGTCCGCCTGCGACACGCCATACTT 2280
bmr-6                   2221:GCAAGAGGTTCCTCCTGCCCATTGGAAGACCTCTAGGTCCGCCTGCGACACGCCATACTT 2280
                             ************************************************************

BTx623                  2281:CAGGGCTTCACCAATTCCTGAGCAAGAGACCACTGCACAATGGAAGACCTCTAGATCTGC 2340
Dwarf white milo        2281:CAGGGCTTCACCAATTCCTGAGCAAGAGACCACTGCACAATGGAAGACCTCTAGATCTGC 2340
Tall white sooner milo  2281:CAGGGCTTCACCAATTCCTGAGCAAGAGACCACTGCACAATGGAAGACCTCTAGATCTGC 2340
bmr-6                   2281:CAGGGCTTCACCAATTCCTGAGCAAGAGACCACTGCACAATGGAAGACCTCTAGATCTGC 2340
                             ************************************************************

BTx623                  2341:TTGTGACACACCGTATGCCAGGAACTCACCAACCAATATTTTTGGTCTGGATTCATCTAC 2400
Dwarf white milo        2341:TTGTGACACACCGTATGCCAGGAACTCACCAACCAATATTTTTGGTCTGGATTCATCTAC 2400
Tall white sooner milo  2341:TTGTGACACACCGTATGCCAGGAACTCACCAACCAATATTTTTGGTCTGGATTCATCTAC 2400
bmr-6                   2341:TTGTGACACACCGTATGCCAGGAACTCACCAACCAATATTTTTGGTCTGGATTCATCTAC 2400
                             ************************************************************

BTx623                  2401:CCCGAGAAACTATATGCTAGATAGCAATTTCTTTCGGCCAGCGGCATCTGCTCAATTCTA 2460
Dwarf white milo        2401:CCCGAGAAACTATATGCTAGATAGCAATTTCTTTCGGCCAGCGGCATCTGCTCAATTCTA 2460
Tall white sooner milo  2401:CCCGAGAAACTATATGCTAGATAGCAATTTCTTTCGGCCAGCGGCATCTGCTCAATTCTA 2460
bmr-6                   2401:CCCGAGAAACTATATGCTAGATAGCAATTTCTTTCGGCCAGCGGCATCTGCTCAATTCTA 2460
                             ************************************************************

BTx623                  2461:CCTGGACCAGGCTCAGCAGACGTTTCCACATAATGGCGGGAGGGTTAGTGTATCCAGGGA 2520
Dwarf white milo        2461:CCTGGACCAGGCTCAGCAGACGTTTCCACATAATGGCGGGAGGGTTAGTGTATCCAGGGA 2520
Tall white sooner milo  2461:CCTGGACCAGGCTCAGCAGACGTTTCCACATAATGGCGGGAGGGTTAGTGTATCCAGGGA 2520
bmr-6                   2461:CCTGGACCAGGCTCAGCAGACGTTTCCACATAATGGCGGGAGGGTTAGTGTATCCAGGGA 2520
                             ************************************************************

BTx623                  2521:GAAGCAAGATGCCGATGAAATCGAAGCTTACAGAGCTTCATTTGGGTTTAGTGCTGATGA 2580
Dwarf white milo        2521:GAAGCAAGATGCCGATGAAATCGAAGCTTACAGAGCTTCATTTGGGTTTAGTGCTGATGA 2580
Tall white sooner milo  2521:GAAGCAAGATGCCGATGAAATCGAAGCTTACAGAGCTTCATTTGGGTTTAGTGCTGATGA 2580
bmr-6                   2521:GAAGCAAGATGCCGATGAAATCGAAGCTTACAGAGCTTCATTTGGGTTTAGTGCTGATGA 2580
                             ************************************************************

BTx623                  2581:AATTGTCCAATCTCAATCTTATGTGGGGATACCAGATGCGGTTGATGAGTCATTCAGTAT 2640
Dwarf white milo        2581:AATTGTCCAATCTCAATCTTATGTGGGGATACCAGATGCGGTTGATGAGTCATTCAGTAT 2640
Tall white sooner milo  2581:AATTGTCCAATCTCAATCTTATGTGGGGATACCAGATGCGGTTGATGAGTCATTCAGTAT 2640
bmr-6                   2581:AATTGTCCAATCTCAATCTTATGTGGGGATACCAGATGCGGTTGATGAGTCATTCAGTAT 2640
                             ************************************************************

BTx623                  2641:ATCACCATTTGGAAACAATGCTCCTGCTACTGAGATATGCCCATTTAGTGATCTGCCTAA 2700
Dwarf white milo        2641:ATCACCATTTGGAAACAATGCTCCTGCTACTGAGATATGCCCATTTAGTGATCTGCCTAA 2700
Tall white sooner milo  2641:ATCACCATTTGGAAACAATGCTCCTGCTACTGAGATATGCCCATTTAGTGATCTGCCTAA 2700
bmr-6                   2641:ATCACCATTTGGAAACAATGCTCCTGCTACTGAGATATGCCCATTTAGTGATCTGCCTAA 2700
                             ************************************************************

BTx623                  2701:TGAGGTTCAGAAGGTGGATAAATCATGTGCCTACGCTAAAGATGGCACAAGTCCAAAGAA 2760
Dwarf white milo        2701:TGAGGTTCAGAAGGTGGATAAATCATGTGCCTACGCTAAAGATGGCACAAGTCCAAAGAA 2760
Tall white sooner milo  2701:TGAGGTTCAGAAGGTGGATAAATCATGTGCCTACGCTAAAGATGGCACAAGTCCAAAGAA 2760
bmr-6                   2701:TGAGGTTCAGAAGGTGGATAAATCATGTGCCTACGCTAAAGATGGCACAAGTCCAAAGAA 2760
                             ************************************************************

BTx623                  2761:ATCAGCAAACCAACTCTCCATTGATTCTCCAAATAAAGTTCTGCGCTTGGACGTGTTCAA 2820
Dwarf white milo        2761:ATCAGCAAACCAACTCTCCATTGATTCTCCAAATAAAGTTCTGCGCTTGGACGTGTTCAA 2820
Tall white sooner milo  2761:ATCAGCAAACCAACTCTCCATTGATTCTCCAAATAAAGTTCTGCGCTTGGACGTGTTCAA 2820
bmr-6                   2761:ATCAGCAAACCAACTCTCCATTGATTCTCCAAATAAAGTTCTGCGCTTGGACGTGTTCAA 2820
                             ************************************************************

BTx623                  2821:AGGTAACATTGGATTTTTCTTGGTACTGTGTGAAATAATAATCTGCTGAATTGAAATACA 2880
Dwarf white milo        2821:AGGTAACATTGGATTTTTCTTGGTACTGTGTGAAATAATAATCTGCTGAATTGAAATACA 2880
Tall white sooner milo  2821:AGGTAACATTGGATTTTTCTTGGTACTGTGTGAAATAATAATCTGCTGAATTGAAATACA 2880
bmr-6                   2821:AGGTAACATTGGATTTTTCTTGGTACTGTGTGAAATAATAATCTGCTGAATTGAAATACA 2880
                             ************************************************************
```

Figure S4 (page4)

```
BTx623                  2881:TATTGTAGGCTGATTTGTACACCCAGATTTTAATAGTAACAAATTTGTTATGGCCCTACT 2940
Dwarf white milo        2881:TATTGTAGGCTGATTTGTACACCCAGATTTTAATAGTAACAAATTTGTTATGGCCCTACT 2940
Tall white sooner milo  2881:TATTGTAGGCTGATTTGTACACCCAGATTTTAATAGTAACAAATTTGTTATGGCCCTACT 2940
bmr-6                   2881:TATTGTAGGCTGATTTGTACACCCAGATTTTAATAGTAACAAATTTGTTATGGCCCTACT 2940
                             ************************************************************

BTx623                  2941:ATTGTTGTGTTTCTGTTTCCACATGCTTATTGTGTTTCTGTTCTCAAATGGCAGGAACAA 3000
Dwarf white milo        2941:ATTGTTGTGTTTCTGTTTCCACATGCTTATTGTGTTTCTGTTCTCAAATGGCAGGAACAA 3000
Tall white sooner milo  2941:ATTGTTGTGTTTCTGTTTCCACATGCTTATTGTGTTTCTGTTCTCAAATGGCAGGAACAA 3000
bmr-6                   2941:ATTGTTGTGTTTCTGTTTCCACATGCTTATTGTGTTTCTGTTCTCAAATGGCAGGAACAA 3000
                             ************************************************************

BTx623                  3001:AAGGAGGTCATCAGTCTGAGGACGAGGGTATTGTGAAAGATGGTCATCCTTTCAGAAGGA 3060
Dwarf white milo        3001:AAGGAGGTCATCAGTCTGAGGACGAGGGTATTGTGAAAGATGGTCATCCTTTCAGAAGGA 3060
Tall white sooner milo  3001:AAGGAGGTCATCAGTCTGAGGACGAGGGTATTGTGAAAGATGGTCATCCTTTCAGAAGGA 3060
bmr-6                   3001:AAGGAGGTCATCAGTCTGAGGACGAGGGTATTGTGAAAGATGGTCATCCTTTCAGAAGGA 3060
                             ************************************************************

BTx623                  3061:CAACAGATGAAATATCTCTAAAACCCATAGAAGTAAGGAAGAAATCTCTTCCTGGCCATT 3120
Dwarf white milo        3061:CAACAGATGAAATATCTCTAAAACCCATAGAAGTAAGGAAGAAATCTCTTCCTGGCCATT 3120
Tall white sooner milo  3061:CAACAGATGAAATATCTCTAAAACCCATAGAAGTAAGGAAGAAATCTCTTCCTGGCCATT 3120
bmr-6                   3061:CAACAGATGAAATATCTCTAAAACCCATAGAAGTAAGGAAGAAATCTCTTCCTGGCCATT 3120
                             ************************************************************

BTx623                  3121:CTTGCTCAGATGCAGAAATCGAGTACAGAAGGACAAGGAGTTTGAGGGATGCCAATGGTG 3180
Dwarf white milo        3121:CTTGCTCAGATGCAGAAATCGAGTACAGAAGGACAAGGAGTTTGAGGGATGCCAATGGTG 3180
Tall white sooner milo  3121:CTTGCTCAGATGCAGAAATCGAGTACAGAAGGACAAGGAGTTTGAGGGATGCCAATGGTG 3180
bmr-6                   3121:CTTGCTCAGATGCAGAAATCGAGTACAGAAGGACAAGGAGTTTGAGGGATGCCAATGGTG 3180
                             ************************************************************

BTx623                  3181:TTTTATCGCGGCGGAGTGCATTGGCAAGACAATTGCATTAATAAGTCACTAGTATGCAGG 3240
Dwarf white milo        3181:TTTTATCGCGGCGGAGTGCATTGGCAAGACAATTGCATTAATAAGTCACTAGTATGCAGG 3240
Tall white sooner milo  3181:TTTTATCGCGGCGGAGTGCATTGGCAAGACAATTGCATTAATAAGTCACTAGTATGCAGG 3240
bmr-6                   3181:TTTTATCGCGGCGGAGTGCATTGGCAAGACAATTGCATTAATAAGTCACTAGTATGCAGG 3240
                             ************************************************************

BTx623                  3241:GTAATTTCATAGTTCATGTAGCCTAATGTGCATTGAATTCGAGCTGTCATTAGAGTGATC 3300
Dwarf white milo        3241:GTAATTTCATAGTTCATGTAGCCTAATGTGCATTGAATTCGAGCTGTCATTAGAGTGATC 3300
Tall white sooner milo  3241:GTAATTTCATAGTTCATGTAGCCTAATGTGCATTGAATTCGAGCTGTCATTAGAGTGATC 3300
bmr-6                   3241:GTAATTTCATAGTTCATGTAGCCTAATGTGCATTGAATTCGAGCTGTCATTAGAGTGATC 3300
                             ************************************************************

BTx623                  3301:TGTAGCCGAAGAGTAGCCTTTATCGTATATGAGGATTATATTACCAGTTTGTGTTCTAGA 3360
Dwarf white milo        3301:TGTAGCCGAAGAGTAGCCTTTATCGTATATGAGGATTATATTACCAGTTTGTGTTCTAGA 3360
Tall white sooner milo  3301:TGTAGCCGAAGAGTAGCCTTTATCGTATATGAGGATTATATTACCAGTTTGTGTTCTAGA 3360
bmr-6                   3301:TGTAGCCGAAGAGTAGCCTTTATCGTATATGAGGATTATATTACCAGTTTGTGTTCTAGA 3360
                             ************************************************************

BTx623                  3361:TATGTGATGATGAGCTTGTACTCTGTTTGAACTCCAAGATCCTCTTTGTAGACACTCCAA 3420
Dwarf white milo        3361:TATGTGATGATGAGCTTGTACTCTGTTTGAACTCCAAGATCCTCTTTGTAGACACTCCAA 3420
Tall white sooner milo  3361:TATGTGATGATGAGCTTGTACTCTGTTTGAACTCCAAGATCCTCTTTGTAGACACTCCAA 3420
bmr-6                   3361:TATGTGATGATGAGCTTGTACTCTGTTTGAACTCCAAGATCCTCTTTGTAGACACTCCAA 3420
                             ************************************************************

BTx623                  3421:TCGTTCGCAGGAGCAGTGAAATGAAATATCGTTGGTACATTATTACAGGAGTTGTGTTTT 3480
Dwarf white milo        3421:TCGTTCGCAGGAGCAGTGAAATGAAATATCGTTGGTACATTATTACAGGAGTTGTGTTTT 3480
Tall white sooner milo  3421:TCGTTCGCAGGAGCAGTGAAATGAAATATCGTTGGTACATTATTACAGGAGTTGTGTTTT 3480
bmr-6                   3421:TCGTTCGCAGGAGCAGTGAAATGAAATATCGTTGGTACATTATTACAGGAGTTGTGTTTT 3480
                             ************************************************************

BTx623                  3481:GGTCTTACTGATGATGTAGGCTGTGTTTGAATTTTGCAGCCTGCTGATTGGGAATCTTTG 3540
Dwarf white milo        3481:GGTCTTACTGATGATGTAGGCTGTGTTTGAATTTTGCAGCCTGCTGATTGGGAATCTTTG 3540
Tall white sooner milo  3481:GGTCTTACTGATGATGTAGGCTGTGTTTGAATTTTGCAGCCTGCTGATTGGGAATCTTTG 3540
bmr-6                   3481:GGTCTTACTGATGATGTAGGCTGTGTTTGAATTTTGCAGCCTGCTGATTGGGAATCTTTG 3540
                             ************************************************************

BTx623                  3541:TTCTTTTTATTTGCATGTCAGCAAGACAGCAACTTGTGTAACACAATCAATCTGACGTCT 3600
Dwarf white milo        3541:TTCTTTTTATTTGCATGTCAGCAAGACAGCAACTTGTGTAACACAATCAATCTGACGTCT 3600
Tall white sooner milo  3541:TTCTTTTTATTTGCATGTCAGCAAGACAGCAACTTGTGTAACACAATCAATCTGACGTCT 3600
bmr-6                   3541:TTCTTTTTATTTGCATGTCAGCAAGACAGCAACTTGTGTAACACAATCAATCTGACGTCT 3600
                             ************************************************************
```

Figure S4 (page5)

```
BTx623                  3601:AGGTTCTTATATATATACGCAAAGAAAGCAACAGCTGGTCGACGTCAAGAATATTTTGAA 3660
Dwarf white milo        3601:AGGTTCTTATATATATACGCAAAGAAAGCAACAGCTGGTCGACGTCAAGAATATTTTGAA 3660
Tall white sooner milo  3601:AGGTTCTTATATATATACGCAAAGAAAGCAACAGCTGGTCGACGTCAAGAATATTTTGAA 3660
bmr-6                   3601:AGGTTCTTATATATATACGCAAAGAAAGCAACAGCTGGTCGACGTCAAGAATATTTTGAA 3660
                             ************************************************************

BTx623                  3661:CTGTTTTTCACTTGTCGATGATTATTATTTATTCTGCTACATAAAAAAAACTTTTGACAC 3720
Dwarf white milo        3661:CTGTTTTTCACTTGTCGATGATTATTATTTATTCTGCTACATAAAAAAAACTTTTGACAC 3720
Tall white sooner milo  3661:CTGTTTTTCACTTGTCGATGATTATTATTTATTCTGCTACATAAAAAAAACTTTTGACAC 3720
bmr-6                   3661:CTGTTTTTCACTTGTCGATGATTATTATTTATTCTGCTACATAAAAAAAACTTTTGACAC 3720
                             ************************************************************

BTx623                  3721:ATCTGCTGCTCCTCTGACGTAACGGAGAGACGTCACCTTTGCCGTTCAAACTAATCGGCG 3780
Dwarf white milo        3721:ATCTGCTGCTCCTCTGACGTAACGGAGAGACGTCACCTTTGCCGTTCAAACTAATCGGCG 3780
Tall white sooner milo  3721:ATCTGCTGCTCCTCTGACGTAACGGAGAGACGTCACCTTTGCCGTTCAAACTAATCGGCG 3780
bmr-6                   3721:ATCTGCTGCTCCTCTGACGTAACGGAGAGACGTCACCTTTGCCGTTCAAACTAATCGGCG 3780
                             ************************************************************

BTx623                  3781:CCAACGCTCCTCCCGGCATGAGTATATTTGTTTTAGGTCTTGTTTAGTTCCAGAAAATTT 3840
Dwarf white milo        3781:CCAACGCTCCTCCCGGCATGAGTATATTTGTTTTAGGTCTTGTTTAGTTCCAGAAAATTT 3840
Tall white sooner milo  3781:CCAACGCTCCTCCCGGCATGAGTATATTTGTTTTAGGTCTTGTTTAGTTCCAGAAAATTT 3840
bmr-6                   3781:CCAACGCTCCTCCCGGCATGAGTATATTTGTTTTAGGTCTTGTTTAGTTCCAGAAAATTT 3840
                             ************************************************************

BTx623                  3841:TGCAAATTGTTTCGGATTTCCCGTCACATCGAATCTTACGGTACATACATGAAACATTAA 3900
Dwarf white milo        3841:TGCAAATTGTTTCGGATTTCCCGTCACATCGAATCTTACGGTACATACATGAAACATTAA 3900
Tall white sooner milo  3841:TGCAAATTGTTTCGGATTTCCCGTCACATCGAATCTTACGGTACATACATGAAACATTAA 3900
bmr-6                   3841:TGCAAATTGTTTCGGATTTCCCGTCACATCGAATCTTACGGTACATACATGAAACATTAA 3900
                             ************************************************************

BTx623                  3901:ATATAGACAAAAAAATAACTAATTACACAGTTTAGCAGTAATTTACGAGACGAATATTTG 3960
Dwarf white milo        3901:ATATAGACAAAAAAATAACTAATTACACAGTTTAGCAGTAATTTACGAGACGAATATTTG 3960
Tall white sooner milo  3901:ATATAGACAAAAAAATAACTAATTACACAGTTTAGCAGTAATTTACGAGACGAATATTTG 3960
bmr-6                   3901:ATATAGACAAAAAAATAACTAATTACACAGTTTAGCAGTAATTTACGAGACGAATATTTG 3960
                             ************************************************************

BTx623                  3961:TCAAATACAAACGAAAGTGCTACAGTGTGCGTTTTGACAATTTTTTTGAAACTAAACAAG 4020
Dwarf white milo        3961:TCAAATACAAACGAAAGTGCTACAGTGTGCGTTTTGACAATTTTTTTGAAACTAAACAAG 4020
Tall white sooner milo  3961:TCAAATACAAACGAAAGTGCTACAGTGTGCGTTTTGACAATTTTTTTGAAACTAAACAAG 4020
bmr-6                   3961:TCAAATACAAACGAAAGTGCTACAGTGTGCGTTTTGACAATTTTTTTGAAACTAAACAAG 4020
                             ************************************************************

BTx623                  4021:GCCTTAGTCTTCTATGCAATTTTGTGGAAGGCGCTCAAGAATAGCATTTTCAGCAAGGGT 4080
Dwarf white milo        4021:GCCTTAGTCTTCTATGCAATTTTGTGGAAGGCGCTCAAGAATAGCATTTTCAGCAAGGGT 4080
Tall white sooner milo  4021:GCCTTAGTCTTCTATGCAATTTTGTGGAAGGCGCTCAAGAATAGCATTTTCAGCAAGGGT 4080
bmr-6                   4021:GCCTTAGTCTTCTATGCAATTTTGTGGAAGGCGCTCAAGAATAGCATTTTCAGCAAGGGT 4080
                             ************************************************************

BTx623                  4081:AGCATTAGAAGAGGCTGTCATGAATAATGAAGATACAAGTATAAAAACAAAGTGAATATG 4140
Dwarf white milo        4081:AGCATTAGAAGAGGCTGTCATGAATAATGAAGATACAAGTATAAAAACAAAGTGAATATG 4140
Tall white sooner milo  4081:AGCATTAGAAGAGGCTGTCATGAATAATGAAGATACAAGTATAAAAACAAAGTGAATATG 4140
bmr-6                   4081:AGCATTAGAAGAGGCTGTCATGAATAATGAAGATACAAGTATAAAAACAAAGTGAATATG 4140
                             ************************************************************

BTx623                  4141:AAAGCCAGGGCTTGGTTTCGGTGATAATGCTTACGCTTTAAGAATTTTTCGCGTTTATTA 4200
Dwarf white milo        4141:AAAGCCAGGGCTTGGTTTCGGTGATAATGCTTACGCTTTAAGAATTTTTCGCGTTTATTA 4200
Tall white sooner milo  4141:AAAGCCAGGGCTTGGTTTCGGTGATAATGCTTACGCTTTAAGAATTTTTCGCGTTTATTA 4200
bmr-6                   4141:AAAGCCAGGGCTTGGTTTCGGTGATAATGCTTACGCTTTAAGAATTTTTCGCGTTTATTA 4200
                             ************************************************************

BTx623                  4201:GTCGATTAGATGTTACATTATCGTTAGGCAAACGTTTGCTGTGGTTTTGGTGTCTTTTTA 4260
Dwarf white milo        4201:GTCGATTAGATGTTACATTATCGTTAGGCAAACGTTTGCTGTGGTTTTGGTGTCTTTTTA 4260
Tall white sooner milo  4201:GTCGATTAGATGTTACATTATCGTTAGGCAAACGTTTGCTGTGGTTTTGGTGTCTTTTTA 4260
bmr-6                   4201:GTCGATTAGATGTTACATTATCGTTAGGCAAACGTTTGCTGTGGTTTTGGTGTCTTTTTA 4260
                             ************************************************************

BTx623                  4261:ATTGCATGCTGCTGCTTATGTTAAGTAGTAGTATCCATCCTCTGTCATCCTGTTACAAAT 4320
Dwarf white milo        4261:ATTGCATGCTGCTGCTTATGTTAAGTAGTAGTATCCATCCTCTGTCATCCTGTTACAAAT 4320
Tall white sooner milo  4261:ATTGCATGCTGCTGCTTATGTTAAGTAGTAGTATCCATCCTCTGTCATCCTGTTACAAAT 4320
bmr-6                   4261:ATTGCATGCTGCTGCTTATGTTAAGTAGTAGTATCCATCCTCTGTCATCCTGTTACAAAT 4320
                             ************************************************************
```

Figure S4 (page6)

```
BTx623                 4321:GATCTGTTATTGTTGTTTTGTAATGGGCCTGTTTGGTTCCCTTCATTAAAACTTAGCTAC 4380
Dwarf white milo       4321:GATCTGTTATTGTTGTTTTGTAATGGGCCTGTTTGGTTCCCTTCATTAAAACTTAGCTAC 4380
Tall white sooner milo 4321:GATCTGTTATTGTTGTTTTGTAATGGGCCTGTTTGGTTCCCTTCATTAAAACTTAGCTAC 4380
bmr-6                  4321:GATCTGTTATTGTTGTTTTGTAATGGGCCTGTTTGGTTCCCTTCATTAAAACTTAGCTAC 4380
                            ************************************************************

BTx623                 4381:TAAAACTATTTTAGCTACTCTTAGGTAACTAGTGGGACTAAAGTATTTTATCTTCTTTTT 4440
Dwarf white milo       4381:TAAAACTATTTTAGCTACTCTTAGGTAACTAGTGGGACTAAAGTATTTTATCTTCTTTTT 4440
Tall white sooner milo 4381:TAAAACTATTTTAGCTACTCTTAGGTAACTAGTGGGACTAAAGTATTTTATCTTCTTTTT 4440
bmr-6                  4381:TAAAACTATTTTAGCTACTCTTAGGTAACTAGTGGGACTAAAGTATTTTATCTTCTTTTT 4440
                            ************************************************************

BTx623                 4441:AGTTAGTGTGTTTGAAAGTTTAGCTACTAAAGTGACTAAAGTTTAGCTGACTAAAATTTA 4500
Dwarf white milo       4441:AGTTAGTGTGTTTGAAAGTTTAGCTACTAAAGTGACTAAAGTTTAGCTGACTAAAATTTA 4500
Tall white sooner milo 4441:AGTTAGTGTGTTTGAAAGTTTAGCTACTAAAGTGACTAAAGTTTAGCTGACTAAAATTTA 4500
bmr-6                  4441:AGTTAGTGTGTTTGAAAGTTTAGCTACTAAAGTGACTAAAGTTTAGCTGACTAAAATTTA 4500
                            ************************************************************

BTx623                 4501:GTAAGGTGAACCAAACAGGCCCATTGTAAATAAAGGACTAGAATTTGTAACAATATAGGA 4560
Dwarf white milo       4501:GTAAGGTGAACCAAACAGGCCCATTGTAAATAAAGGACTAGAATTTGTAACAATATAGGA 4560
Tall white sooner milo 4501:GTAAGGTGAACCAAACAGGCCCATTGTAAATAAAGGACTAGAATTTGTAACAATATAGGA 4560
bmr-6                  4501:GTAAGGTGAACCAAACAGGCCCATTGTAAATAAAGGACTAGAATTTGTAACAATATAGGA 4560
                            ************************************************************

BTx623                 4561:AGTCATAAGGAGTACCCCTAGCATTGTCAAGAGAGAGAAGCTCAATTGAACATGGCGTTA 4620
Dwarf white milo       4561:AGTCATAAGGAGTACCCCTAGCATTGTCAAGAGAGAGAAGCTCAATTGAACATGGCGTTA 4620
Tall white sooner milo 4561:AGTCATAAGGAGTACCCCTAGCATTGTCAAGAGAGAGAAGCTCAATTGAACATGGCGTTA 4620
bmr-6                  4561:AGTCATAAGGAGTACCCCTAGCATTGTCAAGAGAGAGAAGCTCAATTGAACATGGCGTTA 4620
                            ************************************************************

BTx623                 4621:CTAGAAAAATACAGTCGTTTCCCCTCCTTATCTTTTGAAACCATAATACAAGTACAAATC 4680
Dwarf white milo       4621:CTAGAAAAATACAGTCGTTTCCCCTCCTTATCTTTTGAAACCATAATACAAGTACAAATC 4680
Tall white sooner milo 4621:CTAGAAAAATACAGTCGTTTCCCCTCCTTATCTTTTGAAACCATAATACAAGTACAAATC 4680
bmr-6                  4621:CTAGAAAAATACAGTCGTTTCCCCTCCTTATCTTTTGAAACCATAATACAAGTACAAATC 4680
                            ************************************************************

BTx623                 4681:CCACAGGCCCGTCACTGAGCGCCATCTCATGATTATGATCTTATAATTACTTCATGTAAA 4740
Dwarf white milo       4681:CCACAGGCCCGTCACTGAGCGCCATCTCATGATTATGATCTTATAATTACTTCATGTAAA 4740
Tall white sooner milo 4681:CCACAGGCCCGTCACTGAGCGCCATCTCATGATTATGATCTTATAATTACTTCATGTAAA 4740
bmr-6                  4681:CCACAGGCCCGTCACTGAGCGCCATCTCATGATTATGATCTTATAATTACTTCATGTAAA 4740
                            ************************************************************

BTx623                 4741:CATGCCAAGACCTCACATTGGCACCCTTAATCCGGAATGTGATGCATAGTCCTTTGGACA 4800
Dwarf white milo       4741:CATGCCAAGACCTCACATTGGCACCCTTAATCCGGAATGTGATGCATAGTCCTTTGGACA 4800
Tall white sooner milo 4741:CATGCCAAGACCTCACATTGGCACCCTTAATCCGGAATGTGATGCATAGTCCTTTGGACA 4800
bmr-6                  4741:CATGCCAAGACCTCACATTGGCACCCTTAATCCGGAATGTGATGCATAGTCCTTTGGACA 4800
                            ************************************************************

BTx623                 4801:TCTGTGCTATGTACAAAGTTTCTTCCGAAGGCTCTGTGGCAGCATGATCCATCTATCCTC 4860
Dwarf white milo       4801:TCTGTGCTATGTACAAAGTTTCTTCCGAAGGCTCTGTGGCAGCATGATCCATCTATCCTC 4860
Tall white sooner milo 4801:TCTGTGCTATGTACAAAGTTTCTTCCGAAGGCTCTGTGGCAGCATGATCCATCTATCCTC 4860
bmr-6                  4801:TCTGTGCTATGTACAAAGTTTCTTCCGAAGGCTCTGTGGCAGCATGATCCATCTATCCTC 4860
                            ************************************************************

BTx623                 4861:ATACCCCTATAGCAGCTAGTGTCTAGCTATTGGGCTTCTTGTTCCACCCAAATTCTCAAG 4920
Dwarf white milo       4861:ATACCCCTATAGCAGCTAGTGTCTAGCTATTGGGCTTCTTGTTCCACCCAAATTCTCAAG 4920
Tall white sooner milo 4861:ATACCCCTATAGCAGCTAGTGTCTAGCTATTGGGCTTCTTGTTCCACCCAAATTCTCAAG 4920
bmr-6                  4861:ATACCCCTATAGCAGCTAGTGTCTAGCTATTGGGCTTCTTGTTCCACCCAAATTCTCAAG 4920
                            ************************************************************

BTx623                 4921:GTATGAAAACCTCTTGGTTGCAACTGATGACATCTTTGTCAGCTTTGGACCGTTACCCTC 4980
Dwarf white milo       4921:GTATGAAAACCTCTTGGTTGCAACTGATGACATCTTTGTCAGCTTTGGACCGTTACCCTC 4980
Tall white sooner milo 4921:GTATGAAAACCTCTTGGTTGCAACTGATGACATCTTTGTCAGCTTTGGACCGTTACCCTC 4980
bmr-6                  4921:GTATGAAAACCTCTTGGTTGCAACTGATGACATCTTTGTCAGCTTTGGACCGTTACCCTC 4980
                            ************************************************************

BTx623                 4981:CTTGCCATATGTTCTGAGGTCTTGGTCCCGTGGGCCCACGTAGTCACATAGCATCGGCGA 5040
Dwarf white milo       4981:CTTGCCATATGTTCTGAGGTCTTGGTCCCGTGGGCCCACGTAGTCACATAGCATCGGCGA 5040
Tall white sooner milo 4981:CTTGCCATATGTTCTGAGGTCTTGGTCCCGTGGGCCCACGTAGTCACATAGCATCGGCGA 5040
bmr-6                  4981:CTTGCCATATGTTCTGAGGTCTTGGTCCCGTGGGCCCACGTAGTCACATAGCATCGGCGA 5040
                            ************************************************************
```

Figure S4 (page7)

```
BTx623                  5041:TACTGGTTCCATTCTCCTCATCTTCTCTACCTCAGCCTTAGCATCCGAGACAACCTTGTC 5100
Dwarf white milo        5041:TACTGGTTCCATTCTCCTCATCTTCTCTACCTCAGCCTTAGCATCCGAGACAACCTTGTC 5100
Tall white sooner milo  5041:TACTGGTTCCATTCTCCTCATCTTCTCTACCTCAGCCTTAGCATCCGAGACAACCTTGTC 5100
bmr-6                   5041:TACTGGTTCCATTCTCCTCATCTTCTCTACCTCAGCCTTAGCATCCGAGACAACCTTGTC 5100
                             ************************************************************

BTx623                  5101:GGTGTCGTCTGAAGTTAAACTGGCGATTATCATTGATTCTGCGTTCTTGGGATCTTGTGT 5160
Dwarf white milo        5101:GGTGTCGTCTGAAGTTAAACTGGCGATTATCATTGATTCTGCGTTCTTGGGATCTTGTGT 5160
Tall white sooner milo  5101:GGTGTCGTCTGAAGTTAAACTGGCGATTATCATTGATTCTGCGTTCTTGGGATCTTGTGT 5160
bmr-6                   5101:GGTGTCGTCTGAAGTTAAACTGGCGATTATCATTGATTCTGCGTTCTTGGGATCTTGTGT 5160
                             ************************************************************

BTx623                  5161:GGCCAAGTCCGCATTGAGAACATCTTCGATTCGAGACATGACTGTGAAAGCTAAGCTCTC 5220
Dwarf white milo        5161:GGCCAAGTCCGCATTGAGAACATCTTCGATTCGAGACATGACTGTGAAAGCTAAGCTCTC 5220
Tall white sooner milo  5161:GGCCAAGTCCGCATTGAGAACATCTTCGATTCGAGACATGACTGTGAAAGCTAAGCTCTC 5220
bmr-6                   5161:GGCCAAGTCCGCATTGAGAACATCTTCGATTCGAGACATGACTGTGAAAGCTAAGCTCTC 5220
                             ************************************************************

BTx623                  5221:CAGAGTCCTGGAGTAACTCTCCAAAATGGCAAGGCCAACATCCTGATGAAGAAAAAGGGT 5280
Dwarf white milo        5221:CAGAGTCCTGGAGTAACTCTCCAAAATGGCAAGGCCAACATCCTGATGAAGAAAAAGGGT 5280
Tall white sooner milo  5221:CAGAGTCCTGGAGTAACTCTCCAAAATGGCAAGGCCAACATCCTGATGAAGAAAAAGGGT 5280
bmr-6                   5221:CAGAGTCCTGGAGTAACTCTCCAAAATGGCAAGGCCAACATCCTGATGAAGAAAAAGGGT 5280
                             ************************************************************

BTx623                  5281:AGAAGGGAGGTATGCAAATTATGCGAGTAAACAAAGCACTGTGGCGTATCTGAAAAGGCA 5340
Dwarf white milo        5281:AGAAGGGAGGTATGCAAATTATGCGAGTAAACAAAGCACTGTGGCGTATCTGAAAAGGCA 5340
Tall white sooner milo  5281:AGAAGGGAGGTATGCAAATTATGCGAGTAAACAAAGCACTGTGGCGTATCTGAAAAGGCA 5340
bmr-6                   5281:AGAAGGGAGGTATGCAAATTATGCGAGTAAACAAAGCACTGTGGCGTATCTGAAAAGGCA 5340
                             ************************************************************

BTx623                  5341:ATGTTAATTCTGAATGCTTTCTGTACCTTGTTGCATTGGATTTTGCTTATGTCTAGGGAC 5400
Dwarf white milo        5341:ATGTTAATTCTGAATGCTTTCTGTACCTTGTTGCATTGGATTTTGCTTATGTCTAGGGAC 5400
Tall white sooner milo  5341:ATGTTAATTCTGAATGCTTTCTGTACCTTGTTGCATTGGATTTTGCTTATGTCTAGGGAC 5400
bmr-6                   5341:ATGTTAATTCTGAATGCTTTCTGTACCTTGTTGCATTGGATTTTGCTTATGTCTAGGGAC 5400
                             ************************************************************

BTx623                  5401:GACTGGGCGATGCCAGGAAATCTGTGCTTGATAAGGAGAAGAACATTCTCTGCCCTCCCT 5460
Dwarf white milo        5401:GACTGGGCGATGCCAGGAAATCTGTGCTTGATAAGGAGAAGAACATTCTCTGCCCTCCCT 5460
Tall white sooner milo  5401:GACTGGGCGATGCCAGGAAATCTGTGCTTGATAAGGAGAAGAACATTCTCTGCCCTCCCT 5460
bmr-6                   5401:GACTGGGCGATGCCAGGAAATCTGTGCTTGATAAGGAGAAGAACATTCTCTGCCCTCCCT 5460
                             ************************************************************

BTx623                  5461:TCAAACATTCCCCTCTTCTCGTGACTGACGTTATGCCCCCACGACAATTTGCTGTCCTTG 5520
Dwarf white milo        5461:TCAAACATTCCCCTCTTCTCGTGACTGACGTTATGCCCCCACGACAATTTGCTGTCCTTG 5520
Tall white sooner milo  5461:TCAAACATTCCCCTCTTCTCGTGACTGACGTTATGCCCCCACGACAATTTGCTGTCCTTG 5520
bmr-6                   5461:TCAAACATTCCCCTCTTCTCGTGACTGACGTTATGCCCCCACGACAATTTGCTGTCCTTG 5520
                             ************************************************************

BTx623                  5521:TGGGTCATTTTCTTCTGCCAGATGACTACTGAGGCTTCGACTCGGTTCTTCAGATCAACA 5580
Dwarf white milo        5521:TGGGTCATTTTCTTCTGCCAGATGACTACTGAGGCTTCGACTCGGTTCTTCAGATCAACA 5580
Tall white sooner milo  5521:TGGGTCATTTTCTTCTGCCAGATGACTACTGAGGCTTCGACTCGGTTCTTCAGATCAACA 5580
bmr-6                   5521:TGGGTCATTTTCTTCTGCCAGATGACTACTGAGGCTTCGACTCGGTTCTTCAGATCAACA 5580
                             ************************************************************

BTx623                  5581:ATGTTGTGTTCATCCGACAAGTCTACAGAGCGTAGAAGTTCTTCAGGGTCAAAATAATCG 5640
Dwarf white milo        5581:ATGTTGTGTTCATCCGACAAGTCTACAGAGCGTAGAAGTTCTTCAGGGTCAAAATAATCG 5640
Tall white sooner milo  5581:ATGTTGTGTTCATCCGACAAGTCTACAGAGCGTAGAAGTTCTTCAGGGTCAAAATAATCG 5640
bmr-6                   5581:ATGTTGTGTTCATCCGACAAGTCTACAGAGCGTAGAAGTTCTTCAGGGTCAAAATAATCG 5640
                             ************************************************************

BTx623                  5641:TCGGTTATAAGTTTGTACATTGAATCTCCAAGGGTGGACTTCCCATTCTAGCAGACATAT 5700
Dwarf white milo        5641:TCGGTTATAAGTTTGTACATTGAATCTCCAAGGGTGGACTTCCCATTCTAGCAGACATAT 5700
Tall white sooner milo  5641:TCGGTTATAAGTTTGTACATTGAATCTCCAAGGGTGGACTTCCCATTCTAGCAGACATAT 5700
bmr-6                   5641:TCGGTTATAAGTTTGTACATTGAATCTCCAAGGGTGGACTTCCCATTCTAGCAGACATAT 5700
                             ************************************************************

BTx623                  5701:AACAAGAGAAGTTATAGAGTTATATTGTTAGAGCTGTTGTAAACAGAATATGGCAAGGCG 5760
Dwarf white milo        5701:AACAAGAGAAGTTATAGAGTTATATTGTTAGAGCTGTTGTAAACAGAATATGGCAAGGCG 5760
Tall white sooner milo  5701:AACAAGAGAAGTTATAGAGTTATATTGTTAGAGCTGTTGTAAACAGAATATGGCAAGGCG 5760
bmr-6                   5701:AACAAGAGAAGTTATAGAGTTATATTGTTAGAGCTGTTGTAAACAGAATATGGCAAGGCG 5760
                             ************************************************************
```

Figure S4 (page8)

```
BTx623                  5761:GTTAGCAGACTGGTCTTATGGATCACCTTTGGTAGGGACTCCGTGTATGATTCAGGAACC 5820
Dwarf white milo        5761:GTTAGCAGACTGGTCTTATGGATCACCTTTGGTAGGGACTCCGTGTATGATTCAGGAACC 5820
Tall white sooner milo  5761:GTTAGCAGACTGGTCTTATGGATCACCTTTGGTAGGGACTCCGTGTATGATTCAGGAACC 5820
bmr-6                   5761:GTTAGCAGACTGGTCTTATGGATCACCTTTGGTAGGGACTCCGTGTATGATTCAGGAACC 5820
                             ************************************************************

BTx623                  5821:TCCATTTCCATGAGAATGTTAGCATTGATAGCCATTGCTGCCTTCAGCACCTGTGCAACA 5880
Dwarf white milo        5821:TCCATTTCCATGAGAATGTTAGCATTGATAGCCATTGCTGCCTTCAGCACCTGTGCAACA 5880
Tall white sooner milo  5821:TCCATTTCCATGAGAATGTTAGCATTGATAGCCATTGCTGCCTTCAGCACCTGTGCAACA 5880
bmr-6                   5821:TCCATTTCCATGAGAATGTTAGCATTGATAGCCATTGCTGCCTTCAGCACCTGTGCAACA 5880
                             ************************************************************

BTx623                  5881:AGTTCCTTCTGGTTCTGAAGCCATTTCCTGTATGCATCTGAGAGCCCATTAGGAGGAACC 5940
Dwarf white milo        5881:AGTTCCTTCTGGTTCTGAAGCCATTTCCTGTATGCATCTGAGAGCCCATTAGGAGGAACC 5940
Tall white sooner milo  5881:AGTTCCTTCTGGTTCTGAAGCCATTTCCTGTATGCATCTGAGAGCCCATTAGGAGGAACC 5940
bmr-6                   5881:AGTTCCTTCTGGTTCTGAAGCCATTTCCTGTATGCATCTGAGAGCCCATTAGGAGGAACC 5940
                             ************************************************************

BTx623                  5941:CTGACAGTTGGGAGCCACCATTTGTCGTCCTGCCTCGGCATGTTTCCCTTCCCCGATTCA 6000
Dwarf white milo        5941:CTGACAGTTGGGAGCCACCATTTGTCGTCCTGCCTCGGCATGTTTCCCTTCCCCGATTCA 6000
Tall white sooner milo  5941:CTGACAGTTGGGAGCCACCATTTGTCGTCCTGCCTCGGCATGTTTCCCTTCCCCGATTCA 6000
bmr-6                   5941:CTGACAGTTGGGAGCCACCATTTGTCGTCCTGCCTCGGCATGTTTCCCTTCCCCGATTCA 6000
                             ************************************************************

BTx623                  6001:TCTGAATCTTTCTTCACATACCAAAACTCCTGCTTACCCTCGAAACTGTCAAGGTATTCC 6060
Dwarf white milo        6001:TCTGAATCTTTCTTCACATACCAAAACTCCTGCTTACCCTCGAAACTGTCAAGGTATTCC 6060
Tall white sooner milo  6001:TCTGAATCTTTCTTCACATACCAAAACTCCTGCTTACCCTCGAAACTGTCAAGGTATTCC 6060
bmr-6                   6001:TCTGAATCTTTCTTCACATACCAAAACTCCTGCTTACCCTCGAAACTGTCAAGGTATTCC 6060
                             ************************************************************

BTx623                  6061:TATTCCACAGGAGACATATGCAATGCCACAAGTTAGAATTTTAGATATCCATCCCTCTGA 6120
Dwarf white milo        6061:TATTCCACAGGAGACATATGCAATGCCACAAGTTAGAATTTTAGATATCCATCCCTCTGA 6120
Tall white sooner milo  6061:TATTCCACAGGAGACATATGCAATGCCACAAGTTAGAATTTTAGATATCCATCCCTCTGA 6120
bmr-6                   6061:TATTCCACAGGAGACATATGCAATGCCACAAGTTAGAATTTTAGATATCCATCCCTCTGA 6120
                             ************************************************************

BTx623                  6121:AGTCTGAACTGTTCAGTTCAGTAAATTATCAAGATAGAGCATGCCCGTGCTTACAAGGAG 6180
Dwarf white milo        6121:AGTCTGAACTGTTCAGTTCAGTAAATTATCAAGATAGAGCATGCCCGTGCTTACAAGGAG 6180
Tall white sooner milo  6121:AGTCTGAACTGTTCAGTTCAGTAAATTATCAAGATAGAGCATGCCCGTGCTTACAAGGAG 6180
bmr-6                   6121:AGTCTGAACTGTTCAGTTCAGTAAATTATCAAGATAGAGCATGCCCGTGCTTACAAGGAG 6180
                             ************************************************************

BTx623                  6181:CATTGCATCGAGCTTGCGCAACGCAGGGATGTTCATTTGTAGATCCTGACGCTGCTGAGT 6240
Dwarf white milo        6181:CATTGCATCGAGCTTGCGCAACGCAGGGATGTTCATTTGTAGATCCTGACGCTGCTGAGT 6240
Tall white sooner milo  6181:CATTGCATCGAGCTTGCGCAACGCAGGGATGTTCATTTGTAGATCCTGACGCTGCTGAGT 6240
bmr-6                   6181:CATTGCATCGAGCTTGCGCAACGCAGGGATGTTCATTTGTAGATCCTGACGCTGCTGAGT 6240
                             ************************************************************

BTx623                  6241:TATCATTATCTGTTTGACATGAAAACGCGTCAATTTAAGTTGGTTAACGCAGTGGTGTTT 6300
Dwarf white milo        6241:TATCATTATCTGTTTGACATGAAAACGCGTCAATTTAAGTTGGTTAACGCAGTGGTGTTT 6300
Tall white sooner milo  6241:TATCATTATCTGTTTGACATGAAAACGCGTCAATTTAAGTTGGTTAACGCAGTGGTGTTT 6300
bmr-6                   6241:TATCATTATCTGTTTGACATGAAAACGCGTCAATTTAAGTTGGTTAACGCAGTGGTGTTT 6300
                             ************************************************************

BTx623                  6301:AACGCTGTATTGTTGTATAACGAATTGGAAATGAAGCTGAATACTTGAATAGTACAGCAA 6360
Dwarf white milo        6301:AACGCTGTATTGTTGTATAACGAATTGGAAATGAAGCTGAATACTTGAATAGTACAGCAA 6360
Tall white sooner milo  6301:AACGCTGTATTGTTGTATAACGAATTGGAAATGAAGCTGAATACTTGAATAGTACAGCAA 6360
bmr-6                   6301:AACGCTGTATTGTTGTATAACGAATTGGAAATGAAGCTGAATACTTGAATAGTACAGCAA 6360
                             ************************************************************

BTx623                  6361:CAGCAAGTACTGAAATAGGGGGATTTTTTTTACCTCCATGCAGGTCCCATTCTCGGCGAC 6420
Dwarf white milo        6361:CAGCAAGTACTGAAATAGGGGGATTTTTTTTACCTCCATGCAGGTCCCATTCTCGGCGAC 6420
Tall white sooner milo  6361:CAGCAAGTACTGAAATAGGGGGATTTTTTTTACCTCCATGCAGGTCCCATTCTCGGCGAC 6420
bmr-6                   6361:CAGCAAGTACTGAAATAGGGGGATTTTTTTTACCTCCATGCAGGTCCCATTCTCGGCGAC 6420
                             ************************************************************

BTx623                  6421:CTGCCGCGAAGGAACAAATTCAACAATGTGATCGGAGACGGACAAAAGCCAGTCGATCTC 6480
Dwarf white milo        6421:CTGCCGCGAAGGAACAAATTCAACAATGTGATCGGAGACGGACAAAAGCCAGTCGATCTC 6480
Tall white sooner milo  6421:CTGCCGCGAAGGAACAAATTCAACAATGTGATCGGAGACGGACAAAAGCCAGTCGATCTC 6480
bmr-6                   6421:CTGCCGCGAAGGAACAAATTCAACAATGTGATCGGAGACGGACAAAAGCCAGTCGATCTC 6480
                             ************************************************************
```

Figure S4 (page9)

```
BTx623                  6481:CTTCGTCCACCGCGATTTCTGGTCGGCGGCCATGGGCTGCAGACGACGCTGCTCACCAAA 6540
Dwarf white milo        6481:CTTCGTCCACCGCGATTTCTGGTCGGCGGCCATGGGCTGCAGACGACGCTGCTCACCAAA 6540
Tall white sooner milo  6481:CTTCGTCCACCGCGATTTCTGGTCGGCGGCCATGGGCTGCAGACGACGCTGCTCACCAAA 6540
bmr-6                   6481:CTTCGTCCACCGCGATTTCTGGTCGGCGGCCATGGGCTGCAGACGACGCTGCTCACCAAA 6540
                             ************************************************************

BTx623                  6541:CACAGAAGCTGAAAAGATGGCATCGCGAGAAAGAACGGGCAGTTGATCAGGGTTTCCACT 6600
Dwarf white milo        6541:CACAGAAGCTGAAAAGATGGCATCGCGAGAAAGAACGGGCAGTTGATCAGGGTTTCCACT 6600
Tall white sooner milo  6541:CACAGAAGCTGAAAAGATGGCATCGCGAGAAAGAACGGGCAGTTGATCAGGGTTTCCACT 6600
bmr-6                   6541:CACAGAAGCTGAAAAGATGGCATCGCGAGAAAGAACGGGCAGTTGATCAGGGTTTCCACT 6600
                             ************************************************************

BTx623                  6601:AGAAATTGGGCAAAAGCTCAAATTGTGGAATTGCAGAAGGAAAAAAATGATGTTTAGAAA 6660
Dwarf white milo        6601:AGAAATTGGGCAAAAGCTCAAATTGTGGAATTGCAGAAGGAAAAAAATGATGTTTAGAAA 6660
Tall white sooner milo  6601:AGAAATTGGGCAAAAGCTCAAATTGTGGAATTGCAGAAGGAAAAAAATGATGTTTAGAAA 6660
bmr-6                   6601:AGAAATTGGGCAAAAGCTCAAATTGTGGAATTGCAGAAGGAAAAAAATGATGTTTAGAAA 6660
                             ************************************************************

BTx623                  6661:AATAAGGAAAGAGAGGATTCTTGGGTGGGGAATAAAAAAAGAAAGGGGGATTACCTGCAA 6720
Dwarf white milo        6661:AATAAGGAAAGAGAGGATTCTTGGGTGGGGAATAAAAAAAGAAAGGGGGATTACCTGCAA 6720
Tall white sooner milo  6661:AATAAGGAAAGAGAGGATTCTTGGGTGGGGAATAAAAAAAGAAAGGGGGATTACCTGCAA 6720
bmr-6                   6661:AATAAGGAAAGAGAGGATTCTTGGGTGGGGAATAAAAAAAGAAAGGGGGATTACCTGCAA 6720
                             ************************************************************

BTx623                  6721:GATTAGTGATGGCGTTGGACAGGGCAAGAGCAGAGGTCACCCCTTTGCCGGTGCCGGACA 6780
Dwarf white milo        6721:GATTAGTGATGGCGTTGGACAGGGCAAGAGCAGAGGTCACCCCTTTGCCGGTGCCGGACA 6780
Tall white sooner milo  6721:GATTAGTGATGGCGTTGGACAGGGCAAGAGCAGAGGTCACCCCTTTGCCGGTGCCGGACA 6780
bmr-6                   6721:GATTAGTGATGGCGTTGGACAGGGCAAGAGCAGAGGTCACCCCTTTGCCGGTGCCGGACA 6780
                             ************************************************************

BTx623                  6781:TGTCCTCCCCCAGCAGCAGCTTGGAGAACTTCTCCCTCACGATCTCCGCATCTGCAACCC 6840
Dwarf white milo        6781:TGTCCTCCCCCAGCAGCAGCTTGGAGAACTTCTCCCTCACGATCTCCGCATCTGCAACCC 6840
Tall white sooner milo  6781:TGTCCTCCCCCAGCAGCAGCTTGGAGAACTTCTCCCTCACGATCTCCGCATCTGCAACCC 6840
bmr-6                   6781:TGTCCTCCCCCAGCAGCAGCTTGGAGAACTTCTCCCTCACGATCTCCGCATCTGCAACCC 6840
                             ************************************************************

BTx623                  6841:ACAAACGCGAATCATTTCTCCAACAAGATTGCAATTTGCAGCAGCATAACGAGCGTTGGT 6900
Dwarf white milo        6841:ACAAACGCGAATCATTTCTCCAACAAGATTGCAATTTGCAGCAGCATAACGAGCGTTGGT 6900
Tall white sooner milo  6841:ACAAACGCGAATCATTTCTCCAACAAGATTGCAATTTGCAGCAGCATAACGAGCGTTGGT 6900
bmr-6                   6841:ACAAACGCGAATCATTTCTCCAACAAGATTGCAATTTGCAGCAGCATAACGAGCGTTGGT 6900
                             ************************************************************

BTx623                  6901:TGATGGTCCAAGAACAACCGTCACCTGAGGGCGGGCCCTCGTGCCGGACCGGCTTGGGCG 6960
Dwarf white milo        6901:TGATGGTCCAAGAACAACCGTCACCTGAGGGCGGGCCCTCGTGCCGGACCGGCTTGGGCG 6960
Tall white sooner milo  6901:TGATGGTCCAAGAACAACCGTCACCTGAGGGCGGGCCCTCGTGCCGGACCGGCTTGGGCG 6960
bmr-6                   6901:TGATGGTCCAAGAACAACCGTCACCTGAGGGCGGGCCCTCGTGCCGGACCGGCTTGGGCG 6960
                             ************************************************************

BTx623                  6961:GCACCGCGAGCGAGGACGAGCTACCGGCTGCCCCCTTATCCGAAGGGCCACCGGCCAGCG 7020
Dwarf white milo        6961:GCACCGCGAGCGAGGACGAGCTACCGGCTGCCCCCTTATCCGAAGGGCCACCGGCCAGCG 7020
Tall white sooner milo  6961:GCACCGCGAGCGAGGACGAGCTACCGGCTGCCCCCTTATCCGAAGGGCCACCGGCCAGCG 7020
bmr-6                   6961:GCACCGCGAGCGAGGACGAGCTACCGGCTGCCCCCTTATCCGAAGGGCCACCGGCCAGCG 7020
                             ************************************************************

BTx623                  7021:GCGGCGGAAGGTCAACGGACAGCCGGCTCCCCTTGGACCCGCAGCTCCTCGACAGCGCCC 7080
Dwarf white milo        7021:GCGGCGGAAGGTCAACGGACAGCCGGCTCCCCTTGGACCCGCAGCTCCTCGACAGCGCCC 7080
Tall white sooner milo  7021:GCGGCGGAAGGTCAACGGACAGCCGGCTCCCCTTGGACCCGCAGCTCCTCGACAGCGCCC 7080
bmr-6                   7021:GCGGCGGAAGGTCAACGGACAGCCGGCTCCCCTTGGACCCGCAGCTCCTCGACAGCGCCC 7080
                             ************************************************************

BTx623                  7081:GCCTGGTGCAAGGCTGCCCCGGCGCCGGCGCCTGCGACTGCGCCTCCATCGAGGAGGCGT 7140
Dwarf white milo        7081:GCCTGGTGCAAGGCTGCCCCGGCGCCGGCGCCTGCGACTGCGCCTCCATCGAGGAGGCGT 7140
Tall white sooner milo  7081:GCCTGGTGCAAGGCTGCCCCGGCGCCGGCGCCTGCGACTGCGCCTCCATCGAGGAGGCGT 7140
bmr-6                   7081:GCCTGGTGCAAGGCTGCCCCGGCGCCGGCGCCTGCGACTGCGCCTCCATCGAGGAGGCGT 7140
                             ************************************************************

BTx623                  7141:CCGTGCCGTCTGAGGCCAGCGAGAGCGGCGACGACGACGACGCCGACCTCCGGCGCCTGA 7200
Dwarf white milo        7141:CCGTGCCGTCTGAGGCCAGCGAGAGCGGCGACGACGACGACGCCGACCTCCGGCGCCTGA 7200
Tall white sooner milo  7141:CCGTGCCGTCTGAGGCCAGCGAGAGCGGCGACGACGACGACGCCGACCTCCGGCGCCTGA 7200
bmr-6                   7141:CCGTGCCGTCTGAGGCCAGCGAGAGCGGCGACGACGACGACGCCGACCTCCGGCGCCTGA 7200
                             ************************************************************
```

Figure S4 (page10)

```
BTx623                  7201:ACGAGAACCTTTCGAGGCCGTGGCCGATCTTCTGGAACGGCCGGGCCATCACGAGCGCAT 7260
Dwarf white milo        7201:ACGAGAACCTTTCGAGGCCGTGGCCGATCTTCTGGAACGGCCGGGCCATCACGAGCGCAT 7260
Tall white sooner milo  7201:ACGAGAACCTTTCGAGGCCGTGGCCGATCTTCTGGAACGGCCGGGCCATCACGAGCGCAT 7260
bmr-6                   7201:ACGAGAACCTTTCGAGGCCGTGGCCGATCTTCTGGAACGGCCGGGCCATCACGAGCGCAT 7260
                             ************************************************************

BTx623                  7261:GGCTGGCACGCGCGCGCCCGCTACGGCATCCGCAATGTCAACGTGGCGCCTTTTCTTTCT 7320
Dwarf white milo        7261:GGCTGGCACGCGCGCGCCCGCTACGGCATCCGCAATGTCAACGTGGCGCCTTTTCTTTCT 7320
Tall white sooner milo  7261:GGCTGGCACGCGCGCGCCCGCTACGGCATCCGCAATGTCAACGTGGCGCCTTTTCTTTCT 7320
bmr-6                   7261:GGCTGGCACGCGCGCGCCCGCTACGGCATCCGCAATGTCAACGTGGCGCCTTTTCTTTCT 7320
                             ************************************************************

BTx623                  7321:TGGCAGAGAGAGAGGGAGGGAGGCGGTGGGCGCGCGGTGGCTGGCTAGATTTCGGGGACG 7380
Dwarf white milo        7321:TGGCAGAGAGAGAGGGAGGGAGGCGGTGGGCGCGCGGTGGCTGGCTAGATTTCGGGGACG 7380
Tall white sooner milo  7321:TGGCAGAGAGAGAGGGAGGGAGGCGGTGGGCGCGCGGTGGCTGGCTAGATTTCGGGGACG 7380
bmr-6                   7321:TGGCAGAGAGAGAGGGAGGGAGGCGGTGGGCGCGCGGTGGCTGGCTAGATTTCGGGGACG 7380
                             ************************************************************

BTx623                  7381:CTGTGTATGGGAGGTGAGGAGAGGGAGGGGCGGAGGGAGGGAAGCACAAGCTCGGGGCGG 7440
Dwarf white milo        7381:CTGTGTATGGGAGGTGAGGAGAGGGAGGGGCGGAGGGAGGGAAGCACAAGCTCGGGGCGG 7440
Tall white sooner milo  7381:CTGTGTATGGGAGGTGAGGAGAGGGAGGGGCGGAGGGAGGGAAGCACAAGCTCGGGGCGG 7440
bmr-6                   7381:CTGTGTATGGGAGGTGAGGAGAGGGAGGGGCGGAGGGAGGGAAGCACAAGCTCGGGGCGG 7440
                             ************************************************************

BTx623                  7441:GCGTGCGATCTGCTGGAAGGCTGCGGTTGCTGTTCGTGTTCGTGGGTGGCAGGCGGAGCA 7500
Dwarf white milo        7441:GCGTGCGATCTGCTGGAAGGCTGCGGTTGCTGTTCGTGTTCGTGGGTGGCAGGCGGAGCA 7500
Tall white sooner milo  7441:GCGTGCGATCTGCTGGAAGGCTGCGGTTGCTGTTCGTGTTCGTGGGTGGCAGGCGGAGCA 7500
bmr-6                   7441:GCGTGCGATCTGCTGGAAGGCTGCGGTTGCTGTTCGTGTTCGTGGGTGGCAGGCGGAGCA 7500
                             ************************************************************

BTx623                  7501:GAATGTGGTTGGTTTCTCGGCTTTGCTTTGCCCCACGCCAAGCTTGGAAAACGGCAGCGT 7560
Dwarf white milo        7501:GAATGTGGTTGGTTTCTCGGCTTTGCTTTGCCCCACGCCAAGCTTGGAAAACGGCAGCGT 7560
Tall white sooner milo  7501:GAATGTGGTTGGTTTCTCGGCTTTGCTTTGCCCCACGCCAAGCTTGGAAAACGGCAGCGT 7560
bmr-6                   7501:GAATGTGGTTGGTTTCTCGGCTTTGCTTTGCCCCACGCCAAGCTTGGAAAACGGCAGCGT 7560
                             ************************************************************

BTx623                  7561:TCCCGGCATGCAGCGCCAGGCTTTTTTATCGTGCGCGGCCGCCGGACTCCGTGCGGTCCG 7620
Dwarf white milo        7561:TCCCGGCATGCAGCGCCAGGCTTTTTTATCGTGCGCGGCCGCCGGACTCCGTGCGGTCCG 7620
Tall white sooner milo  7561:TCCCGGCATGCAGCGCCAGGCTTTTTTATCGTGCGCGGCCGCCGGACTCCGTGCGGTCCG 7620
bmr-6                   7561:TCCCGGCATGCAGCGCCAGGCTTTTTTATCGTGCGCGGCCGCCGGACTCCGTGCGGTCCG 7620
                             ************************************************************

BTx623                  7621:GCTGGCTGGCCGCCAAATATAAAGAGCACTGAGCACGGACGCCTTGATAGCACAGCACGG 7680
Dwarf white milo        7621:GCTGGCTGGCCGCCAAATATAAAGAGCACTGAGCACGGACGCCTTGATAGCACAGCACGG 7680
Tall white sooner milo  7621:GCTGGCTGGCCGCCAAATATAAAGAGCACTGAGCACGGACGCCTTGATAGCACAGCACGG 7680
bmr-6                   7621:GCTGGCTGGCCGCCAAATATAAAGAGCACTGAGCACGGACGCCTTGATAGCACAGCACGG 7680
                             ************************************************************

BTx623                  7681:CGCCACGGCCTGCGAAATCTTCCGCTGCTCGTGCGATAATGGCAGGAACCGTGCCGTGCA 7740
Dwarf white milo        7681:CGCCACGGCCTGCGAAATCTTCCGCTGCTCGTGCGATAATGGCAGGAACCGTGCCGTGCA 7740
Tall white sooner milo  7681:CGCCACGGCCTGCGAAATCTTCCGCTGCTCGTGCGATAATGGCAGGAACCGTGCCGTGCA 7740
bmr-6                   7681:CGCCACGGCCTGCGAAATCTTCCGCTGCTCGTGCGATAATGGCAGGAACCGTGCCGTGCA 7740
                             ************************************************************

BTx623                  7741:TGGTGGCGTTGGCCTCTCGTCCTGCTTGCGCTTGGCCCAGCTCTTAATCGGTGGATGAGT 7800
Dwarf white milo        7741:TGGTGGCGTTGGCCTCTCGTCCTGCTTGCGCTTGGCCCAGCTCTTAATCGGTGGATGAGT 7800
Tall white sooner milo  7741:TGGTGGCGTTGGCCTCTCGTCCTGCTTGCGCTTGGCCCAGCTCTTAATCGGTGGATGAGT 7800
bmr-6                   7741:TGGTGGCGTTGGCCTCTCGTCCTGCTTGCGCTTGGCCCAGCTCTTAATCGGTGGATGAGT 7800
                             ************************************************************

BTx623                  7801:ACAAATTCCTCATTGCCGTTACTTTTGCCTTTTGAAGAACGTTAGTTGAAACCATAATAT 7860
Dwarf white milo        7801:ACAAATTCCTCATTGCCGTTACTTTTGCCTTTTGAAGAACGTTAGTTGAAACCATAATAT 7860
Tall white sooner milo  7801:ACAAATTCCTCATTGCCGTTACTTTTGCCTTTTGAAGAACGTTAGTTGAAACCATAATAT 7860
bmr-6                   7801:ACAAATTCCTCATTGCCGTTACTTTTGCCTTTTGAAGAACGTTAGTTGAAACCATAATAT 7860
                             ************************************************************

BTx623                  7861:AGCAGTAAAAAGAAGCCAAATTCTGTAAGGTCTTGATGGAGTGGATTGAAATGATACTCT 7920
Dwarf white milo        7861:AGCAGTAAAAAGAAGCCAAATTCTGTAAGGTCTTGATGGAGTGGATTGAAATGATACTCT 7920
Tall white sooner milo  7861:AGCAGTAAAAAGAAGCCAAATTCTGTAAGGTCTTGATGGAGTGGATTGAAATGATACTCT 7920
bmr-6                   7861:AGCAGTAAAAAGAAGCCAAATTCTGTAAGGTCTTGATGGAGTGGATTGAAATGATACTCT 7920
                             ************************************************************
```

```
BTx623                  7921:AATCTACTTTAACATATATATAAATTAAGATAGATATATACGTATCCAAACAAGACAGTT 7980
Dwarf white milo        7921:AATCTACTTTAACATATATATAAATTAAGATAGATATATACGTATCCAAACAAGACAGTT 7980
Tall white sooner milo  7921:AATCTACTTTAACATATATATAAATTAAGATAGATATATACGTATCCAAACAAGACAGTT 7980
bmr-6                   7921:AATCTACTTTAACATATATATAAATTAAGATAGATATATACGTATCCAAACAAGACAGTT 7980
                             ************************************************************

BTx623                  7981:TCCTCAACTAGGAGCTGGTAAATTTCAAGGTCGTAAGGAGAGCTCAAATTAGTTTGGTTG 8040
Dwarf white milo        7981:TCCTCAACTAGGAGCTGGTAAATTTCAAGGTCGTAAGGAGAGCTCAAATTAGTTTGGTTG 8040
Tall white sooner milo  7981:TCCTCAACTAGGAGCTGGTAAATTTCAAGGTCGTAAGGAGAGCTCAAATTAGTTTGGTTG 8040
bmr-6                   7981:TCCTCAACTAGGAGCTGGTAAATTTCAAGGTCGTAAGGAGAGCTCAAATTAGTTTGGTTG 8040
                             ************************************************************

BTx623                  8041:GTTGCCGCAAAAACTCTGAATCTCTGCCTCGCCACCTTAATTTTTTTTAAGTTGCTCCTA 8100
Dwarf white milo        8041:GTTGCCGCAAAAACTCTGAATCTCTGCCTCGCCACCTTAATTTTTTTTAAGTTGCTCCTA 8100
Tall white sooner milo  8041:GTTGCCGCAAAAACTCTGAATCTCTGCCTCGCCACCTTAATTTTTTTTAAGTTGCTCCTA 8100
bmr-6                   8041:GTTGCCGCAAAAACTCTGAATCTCTGCCTCGCCACCTTAATTTTTTTTAAGTTGCTCCTA 8100
                             ************************************************************

BTx623                  8101:CCGTTCCAAAATAACTAAAGTTTGAGGTTTATTCTAAAATCAAACAATTTACCTTTTGTC 8160
Dwarf white milo        8101:CCGTTCCAAAATAACTAAAGTTTGAGGTTTATTCTAAAATCAAACAATTTACCTTTTGTC 8160
Tall white sooner milo  8101:CCGTTCCAAAATAACTAAAGTTTGAGGTTTATTCTAAAATCAAACAATTTACCTTTTGTC 8160
bmr-6                   8101:CCGTTCCAAAATAACTAAAGTTTGAGGTTTATTCTAAAATCAAACAATTTACCTTTTGTC 8160
                             ************************************************************

BTx623                  8161:TGCAAAAAAACCAATTTACCTTCGATCAAAATTATAAAGAACACCATCATTTATGGGACT 8220
Dwarf white milo        8161:TGCAAAAAAACCAATTTACCTTCGATCAAAATTATAAAGAACACCATCATTTATGGGACT 8220
Tall white sooner milo  8161:TGCAAAAAAACCAATTTACCTTCGATCAAAATTATAAAGAACACCATCATTTATGGGACT 8220
bmr-6                   8161:TGCAAAAAAACCAATTTACCTTCGATCAAAATTATAAAGAACACCATCATTTATGGGACT 8220
                             ************************************************************

BTx623                  8221:AAATAGGTGCTGAAGTTTGGTTTATGCTGCTGCTTATGCTAATTTGTGTGAGAAGCATCA 8280
Dwarf white milo        8221:AAATAGGTGCTGAAGTTTGGTTTATGCTGCTGCTTATGCTAATTTGTGTGAGAAGCATCA 8280
Tall white sooner milo  8221:AAATAGGTGCTGAAGTTTGGTTTATGCTGCTGCTTATGCTAATTTGTGTGAGAAGCATCA 8280
bmr-6                   8221:AAATAGGTGCTGAAGTTTGGTTTATGCTGCTGCTTATGCTAATTTGTGTGAGAAGCATCA 8280
                             ************************************************************

BTx623                  8281:AGAGACGGTGGTTTTGTGCGCAGCACATTCCACTCGTTAATTTAGAATCAAACAATCCAC 8340
Dwarf white milo        8281:AGAGACGGTGGTTTTGTGCGCAGCACATTCCACTCGTTAATTTAGAATCAAACAATCCAC 8340
Tall white sooner milo  8281:AGAGACGGTGGTTTTGTGCGCAGCACATTCCACTCGTTAATTTAGAATCAAACAATCCAC 8340
bmr-6                   8281:AGAGACGGTGGTTTTGTGCGCAGCACATTCCACTCGTTAATTTAGAATCAAACAATCCAC 8340
                             ************************************************************

BTx623                  8341:TCGTCAATCTCGTGCATTTTATTCATGTTCTCGTGCCCCTGTCACGGCCCAGACAGGTAC 8400
Dwarf white milo        8341:TCGTCAATCTCGTGCATTTTATTCATGTTCTCGTGCCCCTGTCACGGCCCAGACAGGTAC 8400
Tall white sooner milo  8341:TCGTCAATCTCGTGCATTTTATTCATGTTCTCGTGCCCCTGTCACGGCCCAGACAGGTAC 8400
bmr-6                   8341:TCGTCAATCTCGTGCATTTTATTCATGTTCTCGTGCCCCTGTCACGGCCCAGACAGGTAC 8400
                             ************************************************************

BTx623                  8401:AGCCCGGCAAAATCATGGGACGAGCCAAAAAAAAACTGGGCTAGGCCAGGCAGTGCAGGA 8460
Dwarf white milo        8401:AGCCCGGCAAAATCATGGGACGAGCCAAAAAAAAACTGGGCTAGGCCAGGCAGTGCAGGA 8460
Tall white sooner milo  8401:AGCCCGGCAAAATCATGGGACGAGCCAAAAAAAAACTGGGCTAGGCCAGGCAGTGCAGGA 8460
bmr-6                   8401:AGCCCGGCAAAATCATGGGACGAGCCAAAAAAAAACTGGGCTAGGCCAGGCAGTGCAGGA 8460
                             ************************************************************

BTx623                  8461:GAACTCGAAAAAACTGGGCCAGACATGTCATCCGTCCCGGCCCGACCCGTGCTTGAGCCC 8520
Dwarf white milo        8461:GAACTCGAAAAAACTGGGCCAGACATGTCATCCGTCCCGGCCCGACCCGTGCTTGAGCCC 8520
Tall white sooner milo  8461:GAACTCGAAAAAACTGGGCCAGACATGTCATCCGTCCCGGCCCGACCCGTGCTTGAGCCC 8520
bmr-6                   8461:GAACTCGAAAAAACTGGGCCAGACATGTCATCCGTCCCGGCCCGACCCGTGCTTGAGCCC 8520
                             ************************************************************

BTx623                  8521:AAACTCCCGGAACCCGATCTCCAGGTCCGCGGCGGCGACGAGCAGCTCGGCCGCCTGCGC 8580
Dwarf white milo        8521:AAACTCCCGGAACCCGATCTCCAGGTCCGCGGCGGCGACGAGCAGCTCGGCCGCCTGCGC 8580
Tall white sooner milo  8521:AAACTCCCGGAACCCGATCTCCAGGTCCGCGGCGGCGACGAGCAGCTCGGCCGCCTGCGC 8580
bmr-6                   8521:AAACTCCCGGAACCCGATCTCCAGGTCCGCGGCGGCGACGAGCAGCTCGGCCGCCTGCGC 8580
                             ************************************************************

BTx623                  8581:GGGCTCCAGTATCTCCACCGCCCGCTTCACGGTGCGCAGCCGCAGCGCGTCCGCCCTGTC 8640
Dwarf white milo        8581:GGGCTCCAGTATCTCCACCGCCCGCTTCACGGTGCGCAGCCGCAGCGCGTCCGCCCTGTC 8640
Tall white sooner milo  8581:GGGCTCCAGTATCTCCACCGCCCGCTTCACGGTGCGCAGCCGCAGCGCGTCCGCCCTGTC 8640
bmr-6                   8581:GGGCTCCAGTATCTCCACCGCCCGCTTCACGGTGCGCAGCCGCAGCGCGTCCGCCCTGTC 8640
                             ************************************************************
```

```
BTx623                 8641:CAGCACGGCCCTGGCCGTGCGCACGAGGCCGGCCACGTCGAGAAGCCCGGACCCGTCCGC 8700
Dwarf white milo       8641:CAGCACGGCCCTGGCCGTGCGCACGAGGCCGGCCACGTCGAGAAGCCCGGACCCGTCCGC 8700
Tall white sooner milo 8641:CAGCACGGCCCTGGCCGTGCGCACGAGGCCGGCCACGTCGAGAAGCCCGGACCCGTCCGC 8700
bmr-6                  8641:CAGCACGGCCCTGGCCGTGCGCACGAGGCCGGCCACGTCGAGAAGCCCGGACCCGTCCGC 8700
                            ************************************************************

BTx623                 8701:CGACGGAGCGGCCACGACGCCGTGGCCCTCCTGCACGAGCGCCATCTCCCGCGCGAGCGC 8760
Dwarf white milo       8701:CGACGGAGCGGCCACGACGCCGTGGCCCTCCTGCACGAGCGCCATCTCCCGCGCGAGCGC 8760
Tall white sooner milo 8701:CGACGGAGCGGCCACGACGCCGTGGCCCTCCTGCACGAGCGCCATCTCCCGCGCGAGCGC 8760
bmr-6                  8701:CGACGGAGCGGCCACGACGCCGTGGCCCTCCTGCACGAGCGCCATCTCCCGCGCGAGCGC 8760
                            ************************************************************

BTx623                 8761:GTCCTCCTCGGCCACCGTGCGGCGCTGCAGGTCGTCGACCTGCGCCAGCTGCGCCGGGGT 8820
Dwarf white milo       8761:GTCCTCCTCGGCCACCGTGCGGCGCTGCAGGTCGTCGACCTGCGCCAGCTGCGCCGGGGT 8820
Tall white sooner milo 8761:GTCCTCCTCGGCCACCGTGCGGCGCTGCAGGTCGTCGACCTGCGCCAGCTGCGCCGGGGT 8820
bmr-6                  8761:GTCCTCCTCGGCCACCGTGCGGCGCTGCAGGTCGTCGACCTGCGCCAGCTGCGCCGGGGT 8820
                            ************************************************************

BTx623                 8821:GAGGTCGCCCAGGTTCCCCGACCGCACGCCGAGGAGGAGGTCCGGCAGCTGCGCCTCGAG 8880
Dwarf white milo       8821:GAGGTCGCCCAGGTTCCCCGACCGCACGCCGAGGAGGAGGTCCGGCAGCTGCGCCTCGAG 8880
Tall white sooner milo 8821:GAGGTCGCCCAGGTTCCCCGACCGCACGCCGAGGAGGAGGTCCGGCAGCTGCGCCTCGAG 8880
bmr-6                  8821:GAGGTCGCCCAGGTTCCCCGACCGCACGCCGAGGAGGAGGTCCGGCAGCTGCGCCTCGAG 8880
                            ************************************************************

BTx623                 8881:GCGGCGCCCGGACTCGGTGTAGAGCAGGTGGACCAGCGTGGTGGGCCGCCACCCGGCGAG 8940
Dwarf white milo       8881:GCGGCGCCCGGACTCGGTGTAGAGCAGGTGGACCAGCGTGGTGGGCCGCCACCCGGCGAG 8940
Tall white sooner milo 8881:GCGGCGCCCGGACTCGGTGTAGAGCAGGTGGACCAGCGTGGTGGGCCGCCACCCGGCGAG 8940
bmr-6                  8881:GCGGCGCCCGGACTCGGTGTAGAGCAGGTGGACCAGCGTGGTGGGCCGCCACCCGGCGAG 8940
                            ************************************************************

BTx623                 8941:CCAGTACGCGGCGCCGCGCTCCGCGGGGGTGGCCCACGGCGCCGACAGCGTCCACACCGG 9000
Dwarf white milo       8941:CCAGTACGCGGCGCCGCGCTCCGCGGGGGTGGCCCACGGCGCCGACAGCGTCCACACCGG 9000
Tall white sooner milo 8941:CCAGTACGCGGCGCCGCGCTCCGCGGGGGTGGCCCACGGCGCCGACAGCGTCCACACCGG 9000
bmr-6                  8941:CCAGTACGCGGCGCCGCGCTCCGCGGGGGTGGCCCACGGCGCCGACAGCGTCCACACCGG 9000
                            ************************************************************

BTx623                 9001:GTCCAGCCCCGCGCGCGCCGCGCAGTAGGATTCCAGGTGGGCCACGAAGCGGCCCACAAG 9060
Dwarf white milo       9001:GTCCAGCCCCGCGCGCGCCGCGCAGTAGGATTCCAGGTGGGCCACGAAGCGGCCCACAAG 9060
Tall white sooner milo 9001:GTCCAGCCCCGCGCGCGCCGCGCAGTAGGATTCCAGGTGGGCCACGAAGCGGCCCACAAG 9060
bmr-6                  9001:GTCCAGCCCCGCGCGCGCCGCGCAGTAGGATTCCAGGTGGGCCACGAAGCGGCCCACAAG 9060
                            ************************************************************

BTx623                 9061:AGCGGGGAGCTGGGCCGCCGCGTCGTCCGACCACCGGGCCGACGCCAGGTCCCGGCGCAG 9120
Dwarf white milo       9061:AGCGGGGAGCTGGGCCGCCGCGTCGTCCGACCACCGGGCCGACGCCAGGTCCCGGCGCAG 9120
Tall white sooner milo 9061:AGCGGGGAGCTGGGCCGCCGCGTCGTCCGACCACCGGGCCGACGCCAGGTCCCGGCGCAG 9120
bmr-6                  9061:AGCGGGGAGCTGGGCCGCCGCGTCGTCCGACCACCGGGCCGACGCCAGGTCCCGGCGCAG 9120
                            ************************************************************

BTx623                 9121:CGAGCGCAGCCCGCGGAACCAGAGGTGGAAGCGCCGGGTCGCCGCCTCAAGCTCCAGCTC 9180
Dwarf white milo       9121:CGAGCGCAGCCCGCGGAACCAGAGGTGGAAGCGCCGGGTCGCCGCCTCAAGCTCCAGCTC 9180
Tall white sooner milo 9121:CGAGCGCAGCCCGCGGAACCAGAGGTGGAAGCGCCGGGTCGCCGCCTCAAGCTCCAGCTC 9180
bmr-6                  9121:CGAGCGCAGCCCGCGGAACCAGAGGTGGAAGCGCCGGGTCGCCGCCTCAAGCTCCAGCTC 9180
                            ************************************************************

BTx623                 9181:CATTCCCGCCGCCGGCCGCACAGGCAGGCAGGCAGGCAGGCGCGCGGCGGGAGGTGTGTC 9240
Dwarf white milo       9181:CATTCCCGCCGCCGGCCGCACAGGCAGGCAGGCAGGCAGGCGCGCGGCGGGAGGTGTGTC 9240
Tall white sooner milo 9181:CATTCCCGCCGCCGGCCGCACAGGCAGGCAGGCAGGCAGGCGCGCGGCGGGAGGTGTGTC 9240
bmr-6                  9181:CATTCCCGCCGCCGGCCGCACAGGCAGGCAGGCAGGCAGGCGCGCGGCGGGAGGTGTGTC 9240
                            ************************************************************

BTx623                 9241:AACGACGACGACGACGACGACGACGAAGAAGCGTGGGCGGTGGGATTTATAGGGTGCGGC 9300
Dwarf white milo       9241:AACGACGACGACGACGACGACGACGAAGAAGCGTGGGCGGTGGGATTTATAGGGTGCGGC 9300
Tall white sooner milo 9241:AACGACGACGACGACGACGACGACGAAGAAGCGTGGGCGGTGGGATTTATAGGGTGCGGC 9300
bmr-6                  9241:AACGACGACGACGACGACGACGACGAAGAAGCGTGGGCGGTGGGATTTATAGGGTGCGGC 9300
                            ************************************************************

BTx623                 9301:CGTGGAGTGGAGGCGATCGGACGACGGACGGACGGCTGCGGGCTGAGCGTATGAGGTGTC 9360
Dwarf white milo       9301:CGTGGAGTGGAGGCGATCGGACGACGGACGGACGGCTGCGGGCTGAGCGTATGAGGTGTC 9360
Tall white sooner milo 9301:CGTGGAGTGGAGGCGATCGGACGACGGACGGACGGCTGCGGGCTGAGCGTATGAGGTGTC 9360
bmr-6                  9301:CGTGGAGTGGAGGCGATCGGACGACGGACGGACGGCTGCGGGCTGAGCGTATGAGGTGTC 9360
                            ************************************************************
```

Figure S4 (page13)

```
BTx623                  9361:GCGAGGTGGCGCGCATGCAGGGGGCCATGTGTGCCACGAGCACTGGCCGTGTGGTTCTGG 9420
Dwarf white milo        9361:GCGAGGTGGCGCGCATGCAGGGGGCCATGTGTGCCACGAGCACTGGCCGTGTGGTTCTGG 9420
Tall white sooner milo  9361:GCGAGGTGGCGCGCATGCAGGGGGCCATGTGTGCCACGAGCACTGGCCGTGTGGTTCTGG 9420
bmr-6                   9361:GCGAGGTGGCGCGCATGCAGGGGGCCATGTGTGCCACGAGCACTGGCCGTGTGGTTCTGG 9420
                             ************************************************************

BTx623                  9421:ACGGCGACGCCGTCACGCCGGCGACGCGCGGGTGGGCTGGGGTGACATGTGGCCCGAGGT 9480
Dwarf white milo        9421:ACGGCGACGCCGTCACGCCGGCGACGCGCGGGTGGGCTGGGGTGACATGTGGCCCGAGGT 9480
Tall white sooner milo  9421:ACGGCGACGCCGTCACGCCGGCGACGCGCGGGTGGGCTGGGGTGACATGTGGCCCGAGGT 9480
bmr-6                   9421:ACGGCGACGCCGTCACGCCGGCGACGCGCGGGTGGGCTGGGGTGACATGTGGCCCGAGGT 9480
                             ************************************************************

BTx623                  9481:GCCAATGGGTTTACCGTCTACTGTAGAGGTGTCGCGACGCGCCGACTGGTTGGTCTGGTC 9540
Dwarf white milo        9481:GCCAATGGGTTTACCGTCTACTGTAGAGGTGTCGCGACGCGCCGACTGGTTGGTCTGGTC 9540
Tall white sooner milo  9481:GCCAATGGGTTTACCGTCTACTGTAGAGGTGTCGCGACGCGCCGACTGGTTGGTCTGGTC 9540
bmr-6                   9481:GCCAATGGGTTTACCGTCTACTGTAGAGGTGTCGCGACGCGCCGACTGGTTGGTCTGGTC 9540
                             ************************************************************

BTx623                  9541:CCAGGGGTCAGCTGTTTTGTTGATGACGCGAGGTGACGGTGACGGTGACGGTGACGGAAG 9600
Dwarf white milo        9541:CCAGGGGTCAGCTGTTTTGTTGATGACGCGAGGTGACGGTGACGGTGACGGTGACGGAAG 9600
Tall white sooner milo  9541:CCAGGGGTCAGCTGTTTTGTTGATGACGCGAGGTGACGGTGACGGTGACGGTGACGGAAG 9600
bmr-6                   9541:CCAGGGGTCAGCTGTTTTGTTGATGACGCGAGGTGACGGTGACGGTGACGGTGACGGAAG 9600
                             ************************************************************

BTx623                  9601:AGCTACTGGTGGCCTTGGAGCGCAGAAGTTTGGGCTGTCATTTTTGGGTTGGATCCCATC 9660
Dwarf white milo        9601:AGCTACTGGTGGCCTTGGAGCGCAGAAGTTTGGGCTGTCATTTTTGGGTTGGATCCCATC 9660
Tall white sooner milo  9601:AGCTACTGGTGGCCTTGGAGCGCAGAAGTTTGGGCTGTCATTTTTGGGTTGGATCCCATC 9660
bmr-6                   9601:AGCTACTGGTGGCCTTGGAGCGCAGAAGTTTGGGCTGTCATTTTTGGGTTGGATCCCATC 9660
                             ************************************************************

BTx623                  9661:GCATCCCATCTGGATGCCTGCAGATATTTTGCACTGGACGACGGTATTGAATCATGTGTG 9720
Dwarf white milo        9661:GCATCCCATCTGGATGCCTGCAGATATTTTGCACTGGACGACGGTATTGAATCATGTGTG 9720
Tall white sooner milo  9661:GCATCCCATCTGGATGCCTGCAGATATTTTGCACTGGACGACGGTATTGAATCATGTGTG 9720
bmr-6                   9661:GCATCCCATCTGGATGCCTGCAGATATTTTGCACTGGACGACGGTATTGAATCATGTGTG 9720
                             ************************************************************

BTx623                  9721:TTACTGTTATTGCACAAGCTCAAAAAAAAGTGTGTGACTGTTCACTCAGGTTTGCTGGAA 9780
Dwarf white milo        9721:TTACTGTTATTGCACAAGCTCAAAAAAAAGTGTGTGACTGTTCACTCAGGTTTGCTGGAA 9780
Tall white sooner milo  9721:TTACTGTTATTGCACAAGCTCAAAAAAAAGTGTGTGACTGTTCACTCAGGTTTGCTGGAA 9780
bmr-6                   9721:TTACTGTTATTGCACAAGCTCAAAAAAAAGTGTGTGACTGTTCACTCAGGTTTGCTGGAA 9780
                             ************************************************************

BTx623                  9781:ACAGGAGAGTCATCTCGGCCTCATCTTGTTTTATAATAGTTGGAGCTGTGGATAGTGCTG 9840
Dwarf white milo        9781:ACAGGAGAGTCATCTCGGCCTCATCTTGTTTTATAATAGTTGGAGCTGTGGATAGTGCTG 9840
Tall white sooner milo  9781:ACAGGAGAGTCATCTCGGCCTCATCTTGTTTTATAATAGTTGGAGCTGTGGATAGTGCTG 9840
bmr-6                   9781:ACAGGAGAGTCATCTCGGCCTCATCTTGTTTTATAATAGTTGGAGCTGTGGATAGTGCTG 9840
                             ************************************************************

BTx623                  9841:TCTGCTAGTGCTGTGCTGTGCTCCCAAGATCAGGTTTCTGAATTTCTACTTTCCGGTAGG 9900
Dwarf white milo        9841:TCTGCTAGTGCTGTGCTGTGCTCCCAAGATCAGGTTTCTGAATTTCTACTTTCCGGTAGG 9900
Tall white sooner milo  9841:TCTGCTAGTGCTGTGCTGTGCTCCCAAGATCAGGTTTCTGAATTTCTACTTTCCGGTAGG 9900
bmr-6                   9841:TCTGCTAGTGCTGTGCTGTGCTCCCAAGATCAGGTTTCTGAATTTCTACTTTCCGGTAGG 9900
                             ************************************************************

BTx623                  9901:TATCAATAATGGAGGTTTTATTTGCCTGGCTGCCGGCCATGGCGATCGGCCGGCGCGTGT 9960
Dwarf white milo        9901:TATCAATAATGGAGGTTTTATTTGCCTGGCTGCCGGCCATGGCGATCGGCCGGCGCGTGT 9960
Tall white sooner milo  9901:TATCAATAATGGAGGTTTTATTTGCCTGGCTGCCGGCCATGGCGATCGGCCGGCGCGTGT 9960
bmr-6                   9901:TATCAATAATGGAGGTTTTATTTGCCTGGCTGCCGGCCATGGCGATCGGCCGGCGCGTGT 9960
                             ************************************************************

BTx623                  9961:CATTAGTAGAATCTTTGTTTCGTCCTCGCGTGTCTCGTTCCTGTCCCAGGAACCTCAAAA 10020
Dwarf white milo        9961:CATTAGTAGAATCTTTGTTTCGTCCTCGCGTGTCTCGTTCCTGTCCCAGGAACCTCAAAA 10020
Tall white sooner milo  9961:CATTAGTAGAATCTTTGTTTCGTCCTCGCGTGTCTCGTTCCTGTCCCAGGAACCTCAAAA 10020
bmr-6                   9961:CATTAGTAGAATCTTTGTTTCGTCCTCGCGTGTCTCGTTCCTGTCCCAGGAACCTCAAAA 10020
                             ************************************************************

BTx623                 10021:GCGGAGGATACGTGTATATCTTGCCAGAACACCAGCACCTTCAGAGTTTCAGACACATGA 10080
Dwarf white milo       10021:GCGGAGGATACGTGTATATCTTGCCAGAACACCAGCACCTTCAGAGTTTCAGACACATGA 10080
Tall white sooner milo 10021:GCGGAGGATACGTGTATATCTTGCCAGAACACCAGCACCTTCAGAGTTTCAGACACATGA 10080
bmr-6                  10021:GCGGAGGATACGTGTATATCTTGCCAGAACACCAGCACCTTCAGAGTTTCAGACACATGA 10080
                             ************************************************************
```

Figure S4 (page14)

```
BTx623                 10081:TGGTGCAATGCAACGGGTGGTAGAAATCGTCGGTTCACGAGGATTACTAGTACTATTCCT 10140
Dwarf white milo       10081:TGGTGCAATGCAACGGGTGGTAGAAATCGTCGGTTCACGAGGATTACTAGTACTATTCCT 10140
Tall white sooner milo 10081:TGGTGCAATGCAACGGGTGGTAGAAATCGTCGGTTCACGAGGATTACTAGTACTATTCCT 10140
bmr-6                  10081:TGGTGCAATGCAACGGGTGGTAGAAATCGTCGGTTCACGAGGATTACTAGTACTATTCCT 10140
                             ************************************************************

BTx623                 10141:GCAGTTTCACATTTACAGCAGGAGATATATATATATATATGCAGTTGTCCAGTTACGTTG 10200
Dwarf white milo       10141:GCAGTTTCACATTTACAGCAGGAGATATATATATATATATGCAGTTGTCCAGTTACGTTG 10200
Tall white sooner milo 10141:GCAGTTTCACATTTACAGCAGGAGATATATATATATATATGCAGTTGTCCAGTTACGTTG 10200
bmr-6                  10141:GCAGTTTCACATTTACAGCAGGAGATATATATATATATATGCAGTTGTCCAGTTACGTTG 10200
                             ************************************************************

BTx623                 10201:ACGATGCTGGGCGATGCACAAAGGTTGCATAGCAGCAGTATGCACAGCGACACAGCCTGC 10260
Dwarf white milo       10201:ACGATGCTGGGCGATGCACAAAGGTTGCATAGCAGCAGTATGCACAGCGACACAGCCTGC 10260
Tall white sooner milo 10201:ACGATGCTGGGCGATGCACAAAGGTTGCATAGCAGCAGTATGCACAGCGACACAGCCTGC 10260
bmr-6                  10201:ACGATGCTGGGCGATGCACAAAGGTTGCATAGCAGCAGTATGCACAGCGACACAGCCTGC 10260
                             ************************************************************

BTx623                 10261:AGGGGCATCCCGTCTTGCCAAGTTAGCTTCCTTCCGCGGTCGAAAGCACCTCCAGCTCGG 10320
Dwarf white milo       10261:AGGGGCATCCCGTCTTGCCAAGTTAGCTTCCTTCCGCGGTCGAAAGCACCTCCAGCTCGG 10320
Tall white sooner milo 10261:AGGGGCATCCCGTCTTGCCAAGTTAGCTTCCTTCCGCGGTCGAAAGCACCTCCAGCTCGG 10320
bmr-6                  10261:AGGGGCATCCCGTCTTGCCAAGTTAGCTTCCTTCCGCGGTCGAAAGCACCTCCAGCTCGG 10320
                             ************************************************************

BTx623                 10321:CCCACCAGAGCGGAGACAGCCTCGGCGTCGCAGCGACCGGCGAGATACCGATGGCTTCTC 10380
Dwarf white milo       10321:CCCACCAGAGCGGAGACAGCCTCGGCGTCGCAGCGACCGGCGAGATACCGATGGCTTCTC 10380
Tall white sooner milo 10321:CCCACCAGAGCGGAGACAGCCTCGGCGTCGCAGCGACCGGCGAGATACCGATGGCTTCTC 10380
bmr-6                  10321:CCCACCAGAGCGGAGACAGCCTCGGCGTCGCAGCGACCGGCGAGATACCGATGGCTTCTC 10380
                             ************************************************************

BTx623                 10381:TCAGCCTGGCAGCGACCTCCCTCATGCTCGGTCTGTTTCTGGGATCGCTCTGGATGCAGG 10440
Dwarf white milo       10381:TCAGCCTGGCAGCGACCTCCCTCATGCTCGGTCTGTTTCTGGGATCGCTCTGGATGCAGG 10440
Tall white sooner milo 10381:TCAGCCTGGCAGCGACCTCCCTCATGCTCGGTCTGTTTCTGGGATCGCTCTGGATGCAGG 10440
bmr-6                  10381:TCAGCCTGGCAGCGACCTCCCTCATGCTCGGTCTGTTTCTGGGATCGCTCTGGATGCAGG 10440
                             ************************************************************

BTx623                 10441:CACGGATCACCTCGCCGATGACTGCTAGCTCATCTTCCCTGTGAATGTGATCGCACAGTG 10500
Dwarf white milo       10441:CACGGATCACCTCGCCGATGACTGCTAGCTCATCTTCCCTGTGAATGTGATCGCACAGTG 10500
Tall white sooner milo 10441:CACGGATCACCTCGCCGATGACTGCTAGCTCATCTTCCCTGTGAATGTGATCGCACAGTG 10500
bmr-6                  10441:CACGGATCACCTCGCCGATGACTGCTAGCTCATCTTCCCTGTGAATGTGATCGCACAGTG 10500
                             ************************************************************

BTx623                 10501:TTGGATCCAGCAAGGAGGCTATACTCCGGTCATCGTTGGCTATGCACTCCAGAGCCTAGG 10560
Dwarf white milo       10501:TTGGATCCAGCAAGGAGGCTATACTCCGGTCATCGTTGGCTATGCACTCCAGAGCCTAGG 10560
Tall white sooner milo 10501:TTGGATCCAGCAAGGAGGCTATACTCCGGTCATCGTTGGCTATGCACTCCAGAGCCTAGG 10560
bmr-6                  10501:TTGGATCCAGCAAGGAGGCTATACTCCGGTCATCGTTGGCTATGCACTCCAGAGCCTAGG 10560
                             ************************************************************

BTx623                 10561:AAAGAAGAAGAAATACAACGTCATCATTTGATTTTTTTTTCATCATCAATCAAGTAGAAA 10620
Dwarf white milo       10561:AAAGAAGAAGAAATACAACGTCATCATTTGATTTTTTTTTCATCATCAATCAAGTAGAAA 10620
Tall white sooner milo 10561:AAAGAAGAAGAAATACAACGTCATCATTTGATTTTTTTTTCATCATCAATCAAGTAGAAA 10620
bmr-6                  10561:AAAGAAGAAGAAATACAACGTCATCATTTGATTTTTTTTTCATCATCAATCAAGTAGAAA 10620
                             ************************************************************

BTx623                 10621:TGATGTTTCATCGATAGCGTCGTCATTACCGAGCTCACGAGGGAGCGCTCGTGTCCGGGG 10680
Dwarf white milo       10621:TGATGTTTCATCGATAGCGTCGTCATTACCGAGCTCACGAGGGAGCGCTCGTGTCCGGGG 10680
Tall white sooner milo 10621:TGATGTTTCATCGATAGCGTCGTCATTACCGAGCTCACGAGGGAGCGCTCGTGTCCGGGG 10680
bmr-6                  10621:TGATGTTTCATCGATAGCGTCGTCATTACCGAGCTCACGAGGGAGCGCTCGTGTCCGGGG 10680
                             ************************************************************

BTx623                 10681:TATGGAAATGGAAGTTTCCCTGAGATGATTTCAAGCAGTAGAACACCGAAGCTATGTACA 10740
Dwarf white milo       10681:TATGGAAATGGAAGTTTCCCTGAGATGATTTCAAGCAGTAGAACACCGAAGCTATGTACA 10740
Tall white sooner milo 10681:TATGGAAATGGAAGTTTCCCTGAGATGATTTCAAGCAGTAGAACACCGAAGCTATGTACA 10740
bmr-6                  10681:TATGGAAATGGAAGTTTCCCTGAGATGATTTCAAGCAGTAGAACACCGAAGCTATGTACA 10740
                             ************************************************************

BTx623                 10741:TTCCCTGCTAACCCAGCGGACACCTGTTCTTGGTGATGGTCGAGCTCGCCATTCGTCGCC 10800
Dwarf white milo       10741:TTCCCTGCTAACCCAGCGGACACCTGTTCTTGGTGATGGTCGAGCTCGCCATTCGTCGCC 10800
Tall white sooner milo 10741:TTCCCTGCTAACCCAGCGGACACCTGTTCTTGGTGATGGTCGAGCTCGCCATTCGTCGCC 10800
bmr-6                  10741:TTCCCTGCTAACCCAGCGGACACCTGTTCTTGGTGATGGTCGAGCTCGCCATTCGTCGCC 10800
                             ************************************************************
```

Figure S4 (page15)

```
BTx623                 10801:GTTTTGGCTTCAGAAATCACTTCATGCCAAACGCTCATGTCCGCTATCTGCAGGAACAAC 10860
Dwarf white milo       10801:GTTTTGGCTTCAGAAATCACTTCATGCCAAACGCTCATGTCCGCTATCTGCAGGAACAAC 10860
Tall white sooner milo 10801:GTTTTGGCTTCAGAAATCACTTCATGCCAAACGCTCATGTCCGCTATCTGCAGGAACAAC 10860
bmr-6                  10801:GTTTTGGCTTCAGAAATCACTTCATGCCAAACGCTCATGTCCGCTATCTGCAGGAACAAC 10860
                             ************************************************************

BTx623                 10861:TCAGGTCTCAGGGTGGATCAAGTTGCAGTTCCTTCACACTGATGCTAACACAGCATGGTT 10920
Dwarf white milo       10861:TCAGGTCTCAGGGTGGATCAAGTTGCAGTTCCTTCACACTGATGCTAACACAGCATGGTT 10920
Tall white sooner milo 10861:TCAGGTCTCAGGGTGGATCAAGTTGCAGTTCCTTCACACTGATGCTAACACAGCATGGTT 10920
bmr-6                  10861:TCAGGTCTCAGGGTGGATCAAGTTGCAGTTCCTTCACACTGATGCTAACACAGCATGGTT 10920
                             ************************************************************

BTx623                 10921:GTTGACACCAAGAGGTTTCAAAATTTGGTAAAACTCAGGGAGTGATAATATGAAGTTGGG 10980
Dwarf white milo       10921:GTTGACACCAAGAGGTTTCAAAATTTGGTAAAACTCAGGGAGTGATAATATGAAGTTGGG 10980
Tall white sooner milo 10921:GTTGACACCAAGAGGTTTCAAAATTTGGTAAAACTCAGGGAGTGATAATATGAAGTTGGG 10980
bmr-6                  10921:GTTGACACCAAGAGGTTTCAAAATTTGGTAAAACTCAGGGAGTGATAATATGAAGTTGGG 10980
                             ************************************************************

BTx623                 10981:TAGGACAAACAAAAGAAAAGAAAACATTAACCAAGACTAGCTTATAGCCTTATGGAACTG 11040
Dwarf white milo       10981:TAGGACAAACAAAAGAAAAGAAAACATTAACCAAGACTAGCTTATAGCCTTATGGAACTG 11040
Tall white sooner milo 10981:TAGGACAAACAAAAGAAAAGAAAACATTAACCAAGACTAGCTTATAGCCTTATGGAACTG 11040
bmr-6                  10981:TAGGACAAACAAAAGAAAAGAAAACATTAACCAAGACTAGCTTATAGCCTTATGGAACTG 11040
                             ************************************************************

BTx623                 11041:GGATGGGAGTGTGTACCTTTGCAGCACCATCTTCAGATAGCAGTATGGAACTGGACTGAA 11100
Dwarf white milo       11041:GGATGGGAGTGTGTACCTTTGCAGCACCATCTTCAGATAGCAGTATGGAACTGGACTGAA 11100
Tall white sooner milo 11041:GGATGGGAGTGTGTACCTTTGCAGCACCATCTTCAGATAGCAGTATGGAACTGGACTGAA 11100
bmr-6                  11041:GGATGGGAGTGTGTACCTTTGCAGCACCATCTTCAGATAGCAGTATGGAACTGGACTGAA 11100
                             ************************************************************

BTx623                 11101:GATCAGGGTGCACCACAGGAGGGCTGAGCTCATGCATGTGCTGGATGCAGTATGCTAGTC 11160
Dwarf white milo       11101:GATCAGGGTGCACCACAGGAGGGCTGAGCTCATGCATGTGCTGGATGCAGTATGCTAGTC 11160
Tall white sooner milo 11101:GATCAGGGTGCACCACAGGAGGGCTGAGCTCATGCATGTGCTGGATGCAGTATGCTAGTC 11160
bmr-6                  11101:GATCAGGGTGCACCACAGGAGGGCTGAGCTCATGCATGTGCTGGATGCAGTATGCTAGTC 11160
                             ************************************************************

BTx623                 11161:CCATGATAATCCGCATCCTACCTCGCCAAGCGATGTGCTCAAAATCCTCAGCTGCAACAA 11220
Dwarf white milo       11161:CCATGATAATCCGCATCCTACCTCGCCAAGCGATGTGCTCAAAATCCTCAGCTGCAACAA 11220
Tall white sooner milo 11161:CCATGATAATCCGCATCCTACCTCGCCAAGCGATGTGCTCAAAATCCTCAGCTGCAACAA 11220
bmr-6                  11161:CCATGATAATCCGCATCCTACCTCGCCAAGCGATGTGCTCAAAATCCTCAGCTGCAACAA 11220
                             ************************************************************

BTx623                 11221:GTTTGCAGAATTGTTAATTACTCGTACACAATATTTACAGAGCTACATGTACAGTTCAAT 11280
Dwarf white milo       11221:GTTTGCAGAATTGTTAATTACTCGTACACAATATTTACAGAGCTACATGTACAGTTCAAT 11280
Tall white sooner milo 11221:GTTTGCAGAATTGTTAATTACTCGTACACAATATTTACAGAGCTACATGTACAGTTCAAT 11280
bmr-6                  11221:GTTTGCAGAATTGTTAATTACTCGTACACAATATTTACAGAGCTACATGTACAGTTCAAT 11280
                             ************************************************************

BTx623                 11281:GGTATGGTTGAATTAGCAAGAACAGTTTCTATATACCATGAAGACTCTCATGAAGTGTCC 11340
Dwarf white milo       11281:GGTATGGTTGAATTAGCAAGAACAGTTTCTATATACCATGAAGACTCTCATGAAGTGTCC 11340
Tall white sooner milo 11281:GGTATGGTTGAATTAGCAAGAACAGTTTCTATATACCATGAAGACTCTCATGAAGTGTCC 11340
bmr-6                  11281:GGTATGGTTGAATTAGCAAGAACAGTTTCTATATACCATGAAGACTCTCATGAAGTGTCC 11340
                             ************************************************************

BTx623                 11341:CATTCGGCGCGTACTCGAGCACCATCATCCTGGTGAAAGGTTCCTCCTCCTCACAGAAGC 11400
Dwarf white milo       11341:CATTCGGCGCGTACTCGAGCACCATCATCCTGGTGAAAGGTTCCTCCTCCTCACAGAAGC 11400
Tall white sooner milo 11341:CATTCGGCGCGTACTCGAGCACCATCATCCTGGTGAAAGGTTCCTCCTCCTCACAGAAGC 11400
bmr-6                  11341:CATTCGGCGCGTACTCGAGCACCATCATCCTGGTGAAAGGTTCCTCCTCCTCACAGAAGC 11400
                             ************************************************************

BTx623                 11401:CAAGCAGATTGATGAAGTTCTTGTGGTTTATTCGCGACAGGGTGTCTATCTGCTCAATTC 11460
Dwarf white milo       11401:CAAGCAGATTGATGAAGTTCTTGTGGTTTATTCGCGACAGGGTGTCTATCTGCTCAATTC 11460
Tall white sooner milo 11401:CAAGCAGATTGATGAAGTTCTTGTGGTTTATTCGCGACAGGGTGTCTATCTGCTCAATTC 11460
bmr-6                  11401:CAAGCAGATTGATGAAGTTCTTGTGGTTTATTCGCGACAGGGTGTCTATCTGCTCAATTC 11460
                             ************************************************************

BTx623                 11461:ATCATTAAAGTAATTCAAGCTAATATGCAAATACACAAACTCAAATGGGTGTGTTGTTCA 11520
Dwarf white milo       11461:ATCATTAAAGTAATTCAAGCTAATATGCAAATACACAAACTCAAATGGGTGTGTTGTTCA 11520
Tall white sooner milo 11461:ATCATTAAAGTAATTCAAGCTAATATGCAAATACACAAACTCAAATGGGTGTGTTGTTCA 11520
bmr-6                  11461:ATCATTAAAGTAATTCAAGCTAATATGCAAATACACAAACTCAAATGGGTGTGTTGTTCA 11520
                             ************************************************************
```

```
BTx623                 11521:CATATATCTAAATAAATGGGCCAAATTCTGATGATAACCTTTCTCCTGAAGCAGGTCTCT 11580
Dwarf white milo       11521:CATATATCTAAATAAATGGGCCAAATTCTGATGATAACCTTTCTCCTGAAGCAGGTCTCT 11580
Tall white sooner milo 11521:CATATATCTAAATAAATGGGCCAAATTCTGATGATAACCTTTCTCCTGAAGCAGGTCTCT 11580
bmr-6                  11521:CATATATCTAAATAAATGGGCCAAATTCTGATGATAACCTTTCTCCTGAAGCAGGTCTCT 11580
                             ************************************************************

BTx623                 11581:GAATGCTCTGACCACTCCTTGCTCGATGTAATCATAGTGGACACCACGGCGATCTCAACC 11640
Dwarf white milo       11581:GAATGCTCTGACCACTCCTTGCTCGATGTAATCATAGTGGACACCACGGCGATCTCAACC 11640
Tall white sooner milo 11581:GAATGCTCTGACCACTCCTTGCTCGATGTAATCATAGTGGACACCACGGCGATCTCAACC 11640
bmr-6                  11581:GAATGCTCTGACCACTCCTTGCTCGATGTAATCATAGTGGACACCACGGCGATCTCAACC 11640
                             ************************************************************

BTx623                 11641:CCAGTTGACAAAGTTCCCTTGTAGACGGTGTAGTGTGGGTAGCTCGCCACGATGTTACTG 11700
Dwarf white milo       11641:CCAGTTGACAAAGTTCCCTTGTAGACGGTGTAGTGTGGGTAGCTCGCCACGATGTTACTG 11700
Tall white sooner milo 11641:CCAGTTGACAAAGTTCCCTTGTAGACGGTGTAGTGTGGGTAGCTCGCCACGATGTTACTG 11700
bmr-6                  11641:CCAGTTGACAAAGTTCCCTTGTAGACGGTGTAGTGTGGGTAGCTCGCCACGATGTTACTG 11700
                             ************************************************************

BTx623                 11701:AAGTCCTCACAGGCACGTTCCAGCTCTGAACGCTGCAACTTTGGTACTCCTGATGAAGCA 11760
Dwarf white milo       11701:AAGTCCTCACAGGCACGTTCCAGCTCTGAACGCTGCAACTTTGGTACTCCTGATGAAGCA 11760
Tall white sooner milo 11701:AAGTCCTCACAGGCACGTTCCAGCTCTGAACGCTGCAACTTTGGTACTCCTGATGAAGCA 11760
bmr-6                  11701:AAGTCCTCACAGGCACGTTCCAGCTCTGAACGCTGCAACTTTGGTACTCCTGATGAAGCA 11760
                             ************************************************************

BTx623                 11761:ATGAGAATCAGCTGGGTTAATTGCAACAAGCAGCAGCTTAAGTTGTACTAGATATCTGAA 11820
Dwarf white milo       11761:ATGAGAATCAGCTGGGTTAATTGCAACAAGCAGCAGCTTAAGTTGTACTAGATATCTGAA 11820
Tall white sooner milo 11761:ATGAGAATCAGCTGGGTTAATTGCAACAAGCAGCAGCTTAAGTTGTACTAGATATCTGAA 11820
bmr-6                  11761:ATGAGAATCAGCTGGGTTAATTGCAACAAGCAGCAGCTTAAGTTGTACTAGATATCTGAA 11820
                             ************************************************************

BTx623                 11821:CAATTCAGACAGCAAAGTATCAACTGAATCTTCCATCCTATTTTTTTTCCCTTGCAAGTA 11880
Dwarf white milo       11821:CAATTCAGACAGCAAAGTATCAACTGAATCTTCCATCCTATTTTTTTTCCCTTGCAAGTA 11880
Tall white sooner milo 11821:CAATTCAGACAGCAAAGTATCAACTGAATCTTCCATCCTATTTTTTTTCCCTTGCAAGTA 11880
bmr-6                  11821:CAATTCAGACAGCAAAGTATCAACTGAATCTTCCATCCTATTTTTTTTCCCTTGCAAGTA 11880
                             ************************************************************

BTx623                 11881:GTACAGATTTTTTTTAAAAAAAAGGGGGGATGTAAAATAATTTTGAGCACATATATGAAG 11940
Dwarf white milo       11881:GTACAGATTTTTTTTAAAAAAAAGGGGGGATGTAAAATAATTTTGAGCACATATATGAAG 11940
Tall white sooner milo 11881:GTACAGATTTTTTTAAAAAAAAAGGGGGGATGTAAAATAATTTTGAGCACATATATGAAG 11940
bmr-6                  11881:GTACAGATTTTTTTTAAAAAAAAGGGGGGATGTAAAATAATTTTGAGCACATATATGAAG 11940
                             **************.*********************************************

BTx623                 11941:GAACTAGTAGCAAATATAGTACTCCCATACAATAGCAGTATGTATACCTGTAGCAAAGGC 12000
Dwarf white milo       11941:GAACTAGTAGCAAATATAGTACTCCCATACAATAGCAGTATGTATACCTGTAGCAAAGGC 12000
Tall white sooner milo 11941:GAACTAGTAGCAAATATAGTACTCCCATACAATAGCAGTATGTATACCTGTAGCAAAGGC 12000
bmr-6                  11941:GAACTAGTAGCAAATATAGTACTCCCATACAATAGCAGTATGTATACCTGTAGCAAAGGC 12000
                             ************************************************************

BTx623                 12001:TTTCTGCAGCTGGCCACTGAGTCCAGTCTTCCAAGGGCCAATGGTCGCAACCGCACTCTT 12060
Dwarf white milo       12001:TTTCTGCAGCTGGCCACTGAGTCCAGTCTTCCAAGGGCCAATGGTCGCAACCGCACTCTT 12060
Tall white sooner milo 12001:TTTCTGCAGCTGGCCACTGAGTCCAGTCTTCCAAGGGCCAATGGTCGCAACCGCACTCTT 12060
bmr-6                  12001:TTTCTGCAGCTGGCCACTGAGTCCAGTCTTCCAAGGGCCAATGGTCGCAACCGCACTCTT 12060
                             ************************************************************

BTx623                 12061:ACGACAAAGAAGCCAGCACGCTAGTCCAGCAAGCAACAGCAGGGCAAGCGCCGGAAGAAC 12120
Dwarf white milo       12061:ACGACAAAGAAGCCAGCACGCTAGTCCAGCAAGCAACAGCAGGGCAAGCGCCGGAAGAAC 12120
Tall white sooner milo 12061:ACGACAAAGAAGCCAGCACGCTAGTCCAGCAAGCAACAGCAGGGCAAGCGCCGGAAGAAC 12120
bmr-6                  12061:ACGACAAAGAAGCCAGCACGCTAGTCCAGCAAGCAACAGCAGGGCAAGCGCCGGAAGAAC 12120
                             ************************************************************

BTx623                 12121:AACCATGTAGAGCCACCTCATAGATCTCGACAAATAAGCCGCCGGCGTCGTTGGTGCCGC 12180
Dwarf white milo       12121:AACCATGTAGAGCCACCTCATAGATCTCGACAAATAAGCCGCCGGCGTCGTTGGTGCCGC 12180
Tall white sooner milo 12121:AACCATGTAGAGCCACCTCATAGATCTCGACAAATAAGCCGCCGGCGTCGTTGGTGCCGC 12180
bmr-6                  12121:AACCATGTAGAGCCACCTCATAGATCTCGACAAATAAGCCGCCGGCGTCGTTGGTGCCGC 12180
                             ************************************************************

BTx623                 12181:CTCGCTCGGATCGTTCTGCCCCCCGTGTCGACTGGGAGACGCCGCCGGAAAAGAGCCGGT 12240
Dwarf white milo       12181:CTCGCTCGGATCGTTCTGCCCCCCGTGTCGACTGGGAGACGCCGCCGGAAAAGAGCCGGT 12240
Tall white sooner milo 12181:CTCGCTCGGATCGTTCTGCCCCCCGTGTCGACTGGGAGACGCCGCCGGAAAAGAGCCGGT 12240
bmr-6                  12181:CTCGCTCGGATCGTTCTGCCCCCCGTGTCGACTGGGAGACGCCGCCGGAAAAGAGCCGGT 12240
                             ************************************************************
```

Figure S4 (page17)

```
BTx623                 12241:TCCATGAGACGGGACATGAGCGTCGACTGGGACGCCATGAACGTTCGCAGGCAGAGGCAG 12300
Dwarf white milo       12241:TCCATGAGACGGGACATGAGCGTCGACTGGGACGCCATGAACGTTCGCAGGCAGAGGCAG 12300
Tall white sooner milo 12241:TCCATGAGACGGGACATGAGCGTCGACTGGGACGCCATGAACGTTCGCAGGCAGAGGCAG 12300
bmr-6                  12241:TCCATGAGACGGGACATGAGCGTCGACTGGGACGCCATGAACGTTCGCAGGCAGAGGCAG 12300
                             ************************************************************

BTx623                 12301:CGCGGCGAGATTTGTGGTGTCCTGCTGAAGTAGTAGCCTCCGATGGCTCGGCTGCGCCGC 12360
Dwarf white milo       12301:CGCGGCGAGATTTGTGGTGTCCTGCTGAAGTAGTAGCCTCCGATGGCTCGGCTGCGCCGC 12360
Tall white sooner milo 12301:CGCGGCGAGATTTGTGGTGTCCTGCTGAAGTAGTAGCCTCCGATGGCTCGGCTGCGCCGC 12360
bmr-6                  12301:CGCGGCGAGATTTGTGGTGTCCTGCTGAAGTAGTAGCCTCCGATGGCTCGGCTGCGCCGC 12360
                             ************************************************************

BTx623                 12361:CGGCAGATCCGCGGCGGTCATGGCAGAATGGAGGGTCAACATGACCAGCACGAGGGAACT 12420
Dwarf white milo       12361:CGGCAGATCCGCGGCGGTCATGGCAGAATGGAGGGTCAACATGACCAGCACGAGGGAACT 12420
Tall white sooner milo 12361:CGGCAGATCCGCGGCGGTCATGGCAGAATGGAGGGTCAACATGACCAGCACGAGGGAACT 12420
bmr-6                  12361:CGGCAGATCCGCGGCGGTCATGGCAGAATGGAGGGTCAACATGACCAGCACGAGGGAACT 12420
                             ************************************************************

BTx623                 12421:CGACGCGTCCATCGTTCGTCGGCGTGCCCAAAGATCGGTCGCATCAGATGAGCAGATAGC 12480
Dwarf white milo       12421:CGACGCGTCCATCGTTCGTCGGCGTGCCCAAAGATCGGTCGCATCAGATGAGCAGATAGC 12480
Tall white sooner milo 12421:CGACGCGTCCATCGTTCGTCGGCGTGCCCAAAGATCGGTCGCATCAGATGAGCAGATAGC 12480
bmr-6                  12421:CGACGCGTCCATCGTTCGTCGGCGTGCCCAAAGATCGGTCGCATCAGATGAGCAGATAGC 12480
                             ************************************************************

BTx623                 12481:CTCTACGCGCTGCGTGCCCTCCCCTGAGAAAAAAAAAGGGATATGGCAGAATCATCGGCG 12540
Dwarf white milo       12481:CTCTACGCGCTGCGTGCCCTCCCCTGAGAAAAAAAAAGGGATATGGCAGAATCATCGGCG 12540
Tall white sooner milo 12481:CTCTACGCGCTGCGTGCCCTCCCCTGAGAAAAAAAAAGGGATATGGCAGAATCATCGGCG 12540
bmr-6                  12481:CTCTACGCGCTGCGTGCCCTCCCCTGAGAAAAAAAAAGGGATATGGCAGAATCATCGGCG 12540
                             ************************************************************

BTx623                 12541:TCGTCAGGGCTGCATGTTTTGAAGCATTAGAGATACTAGTAGAACACTGGATAAAAAAAG 12600
Dwarf white milo       12541:TCGTCAGGGCTGCATGTTTTGAAGCATTAGAGATACTAGTAGAACACTGGATAAAAAAAG 12600
Tall white sooner milo 12541:TCGTCAGGGCTGCATGTTTTGAAGCATTAGAGATACTAGTAGAACACTGGATAAAAAAAG 12600
bmr-6                  12541:TCGTCAGGGCTGCATGTTTTGAAGCATTAGAGATACTAGTAGAACACTGGATAAAAAAAG 12600
                             ************************************************************

BTx623                 12601:AGGTGTTGGGAAATTCAGAGTTTCAGACAGCTCCATGATTGATCGCATCGCGTCACCGAC 12660
Dwarf white milo       12601:AGGTGTTGGGAAATTCAGAGTTTCAGACAGCTCCATGATTGATCGCATCGCGTCACCGAC 12660
Tall white sooner milo 12601:AGGTGTTGGGAAATTCAGAGTTTCAGACAGCTCCATGATTGATCGCATCGCGTCACCGAC 12660
bmr-6                  12601:AGGTGTTGGGAAATTCAGAGTTTCAGACAGCTCCATGATTGATCGCATCGCGTCACCGAC 12660
                             ************************************************************

BTx623                 12661:GACAACTGATCGAAGAAACATGATTGATGAGAGGAAGCAGGGTAGATTGAGAGCTTACAG 12720
Dwarf white milo       12661:GACAACTGATCGAAGAAACATGATTGATGAGAGGAAGCAGGGTAGATTGAGAGCTTACAG 12720
Tall white sooner milo 12661:GACAACTGATCGAAGAAACATGATTGATGAGAGGAAGCAGGGTAGATTGAGAGCTTACAG 12720
bmr-6                  12661:GACAACTGATCGAAGAAACATGATTGATGAGAGGAAGCAGGGTAGATTGAGAGCTTACAG 12720
                             ************************************************************

BTx623                 12721:TGTGAACGAAACGAAGAGGAAGACGATGACGAGGGAAGGGTCGGAGTTGCTTCGTGGCGG 12780
Dwarf white milo       12721:TGTGAACGAAACGAAGAGGAAGACGATGACGAGGGAAGGGTCGGAGTTGCTTCGTGGCGG 12780
Tall white sooner milo 12721:TGTGAACGAAACGAAGAGGAAGACGATGACGAGGGAAGGGTCGGAGTTGCTTCGTGGCGG 12780
bmr-6                  12721:TGTGAACGAAACGAAGAGGAAGACGATGACGAGGGAAGGGTCGGAGTTGCTTCGTGGCGG 12780
                             ************************************************************

BTx623                 12781:TAGTAGTTGGAGGAGCCAATAACACACACTTTTCTTTGTTACCAGTGACAAAGAAAAAAT 12840
Dwarf white milo       12781:TAGTAGTTGGAGGAGCCAATAACACACACTTTTCTTTGTTACCAGTGACAAAGAAAAAAT 12840
Tall white sooner milo 12781:TAGTAGTTGGAGGAGCCAATAACACACACTTTTCTTTGTTACCAGTGACAAAGAAAAAAT 12840
bmr-6                  12781:TAGTAGTTGGAGGAGCCAATAACACACACTTTTCTTTGTTACCAGTGACAAAGAAAAAAT 12840
                             ************************************************************

BTx623                 12841:GGGGGACTGCTTTGCTTGATTGAGATTAACGACTACTAGAGCGAAACCGGCAATCCCGTT 12900
Dwarf white milo       12841:GGGGGACTGCTTTGCTTGATTGAGATTAACGACTACTAGAGCGAAACCGGCAATCCCGTT 12900
Tall white sooner milo 12841:GGGGGACTGCTTTGCTTGATTGAGATTAACGACTACTAGAGCGAAACCGGCAATCCCGTT 12900
bmr-6                  12841:GGGGGACTGCTTTGCTTGATTGAGATTAACGACTACTAGAGCGAAACCGGCAATCCCGTT 12900
                             ************************************************************

BTx623                 12901:GCTCGTCGCAGTTGCAGTTGCGGACAGCGTCACAGTTTTGTCAACGAAGCGTTCAATAAC 12960
Dwarf white milo       12901:GCTCGTCGCAGTTGCAGTTGCGGACAGCGTCACAGTTTTGTCAACGAAGCGTTCAATAAC 12960
Tall white sooner milo 12901:GCTCGTCGCAGTTGCAGTTGCGGACAGCGTCACAGTTTTGTCAACGAAGCGTTCAATAAC 12960
bmr-6                  12901:GCTCGTCGCAGTTGCAGTTGCGGACAGCGTCACAGTTTTGTCAACGAAGCGTTCAATAAC 12960
                             ************************************************************
```

Figure S4 (page18)

```
BTx623                 12961:TGGGGCTGGGGGAATAAAGAACAGCACCCGAGGGACGGAGCCCACTGCCCACCGCTATCG 13020
Dwarf white milo       12961:TGGGGCTGGGGGAATAAAGAACAGCACCCGAGGGACGGAGCCCACTGCCCACCGCTATCG 13020
Tall white sooner milo 12961:TGGGGCTGGGGGAATAAAGAACAGCACCCGAGGGACGGAGCCCACTGCCCACCGCTATCG 13020
bmr-6                  12961:TGGGGCTGGGGGAATAAAGAACAGCACCCGAGGGACGGAGCCCACTGCCCACCGCTATCG 13020
                             ************************************************************

BTx623                 13021:TCTATCGATCTCGTCCATGGCACGGCTCGCGTCGTGTCCGTCCGCACTGGCACGTAGCTC 13080
Dwarf white milo       13021:TCTATCGATCTCGTCCATGGCACGGCTCGCGTCGTGTCCGTCCGCACTGGCACGTAGCTC 13080
Tall white sooner milo 13021:TCTATCGATCTCGTCCATGGCACGGCTCGCGTCGTGTCCGTCCGCACTGGCACGTAGCTC 13080
bmr-6                  13021:TCTATCGATCTCGTCCATGGCACGGCTCGCGTCGTGTCCGTCCGCACTGGCACGTAGCTC 13080
                             ************************************************************

BTx623                 13081:CTTGTCATGGCGCGCCGCCACGGACGCGTAGCGTTTTTTTAGAAAATCGCAGCTGCCGTC 13140
Dwarf white milo       13081:CTTGTCATGGCGCGCCGCCACGGACGCGTAGCGTTTTTTTAGAAAATCGCAGCTGCCGTC 13140
Tall white sooner milo 13081:CTTGTCATGGCGCGCCGCCACGGACGCGTAGCGTTTTTTTAGAAAATCGCAGCTGCCGTC 13140
bmr-6                  13081:CTTGTCATGGCGCGCCGCCACGGACGCGTAGCGTTTTTTTAGAAAATCGCAGCTGCCGTC 13140
                             ************************************************************

BTx623                 13141:GTACTGGGGCGCCGGCCGGCAAGTCAGCCACGTGAACGGTGAACCTAGAGTGCGTAACGA 13200
Dwarf white milo       13141:GTACTGGGGCGCCGGCCGGCAAGTCAGCCACGTGAACGGTGAACCTAGAGTGCGTAACGA 13200
Tall white sooner milo 13141:GTACTGGGGCGCCGGCCGGCAAGTCAGCCACGTGAACGGTGAACCTAGAGTGCGTAACGA 13200
bmr-6                  13141:GTACTGGGGCGCCGGCCGGCAAGTCAGCCACGTGAACGGTGAACCTAGAGTGCGTAACGA 13200
                             ************************************************************

BTx623                 13201:ACCAGAAACTAGTCCAGAATCTGCGTCGCAGTCGCAGCTTGCCCGAAACAAAGACCGCGC 13260
Dwarf white milo       13201:ACCAGAAACTAGTCCAGAATCTGCGTCGCAGTCGCAGCTTGCCCGAAACAAAGACCGCGC 13260
Tall white sooner milo 13201:ACCAGAAACTAGTCCAGAATCTGCGTCGCAGTCGCAGCTTGCCCGAAACAAAGACCGCGC 13260
bmr-6                  13201:ACCAGAAACTAGTCCAGAATCTGCGTCGCAGTCGCAGCTTGCCCGAAACAAAGACCGCGC 13260
                             ************************************************************

BTx623                 13261:GTGCAAAAGCAAGAACCCACCGGGCCGCGCTCGCCGAAAACTTGTCCGTGACTAGCGCCG 13320
Dwarf white milo       13261:GTGCAAAAGCAAGAACCCACCGGGCCGCGCTCGCCGAAAACTTGTCCGTGACTAGCGCCG 13320
Tall white sooner milo 13261:GTGCAAAAGCAAGAACCCACCGGGCCGCGCTCGCCGAAAACTTGTCCGTGACTAGCGCCG 13320
bmr-6                  13261:GTGCAAAAGCAAGAACCCACCGGGCCGCGCTCGCCGAAAACTTGTCCGTGACTAGCGCCG 13320
                             ************************************************************

BTx623                 13321:CGACGACGGCGACCTTGGGGACGGGATGGACTCGAGAACCCATCGATTTCGATTTCGATT 13380
Dwarf white milo       13321:CGACGACGGCGACCTTGGGGACGGGATGGACTCGAGAACCCATCGATTTCGATTTCGATT 13380
Tall white sooner milo 13321:CGACGACGGCGACCTTGGGGACGGGATGGACTCGAGAACCCATCGATTTCGATTTCGATT 13380
bmr-6                  13321:CGACGACGGCGACCTTGGGGACGGGATGGACTCGAGAACCCATCGATTTCGATTTCGATT 13380
                             ************************************************************

BTx623                 13381:TCGATTTCGATTTCTAGCGGGGTTTGCGGCGCCAGAGGCCGTGCCGACGGCGCGACGCCG 13440
Dwarf white milo       13381:TCGATTTCGATTTCTAGCGGGGTTTGCGGCGCCAGAGGCCGTGCCGACGGCGCGACGCCG 13440
Tall white sooner milo 13381:TCGATTTCGATTTCTAGCGGGGTTTGCGGCGCCAGAGGCCGTGCCGACGGCGCGACGCCG 13440
bmr-6                  13381:TCGATTTCGATTTCTAGCGGGGTTTGCGGCGCCAGAGGCCGTGCCGACGGCGCGACGCCG 13440
                             ************************************************************

BTx623                 13441:AACGCGGCACACCCCCCCACGCCTGCGCCTCGGCGCGCGCGGGAGGAAACGGCCGGAGGC 13500
Dwarf white milo       13441:AACGCGGCACACCCCCCCACGCCTGCGCCTCGGCGCGCGCGGGAGGAAACGGCCGGAGGC 13500
Tall white sooner milo 13441:AACGCGGCACACCCCCCCACGCCTGCGCCTCGGCGCGCGCGGGAGGAAACGGCCGGAGGC 13500
bmr-6                  13441:AACGCGGCACACCCCCCCACGCCTGCGCCTCGGCGCGCGCGGGAGGAAACGGCCGGAGGC 13500
                             ************************************************************

BTx623                 13501:TACTATAGCCGTTGCGGCTGGTCCCCCTGAGCAAGTCGTCGGTTTCGGCAGGCGCGCGGG 13560
Dwarf white milo       13501:TACTATAGCCGTTGCGGCTGGTCCCCCTGAGCAAGTCGTCGGTTTCGGCAGGCGCGCGGG 13560
Tall white sooner milo 13501:TACTATAGCCGTTGCGGCTGGTCCCCCTGAGCAAGTCGTCGGTTTCGGCAGGCGCGCGGG 13560
bmr-6                  13501:TACTATAGCCGTTGCGGCTGGTCCCCCTGAGCAAGTCGTCGGTTTCGGCAGGCGCGCGGG 13560
                             ************************************************************

BTx623                 13561:GTCGAGACCGCGAGGCGCCGAGACCCCGAGGTCTGCTGCACTCTGCTACGGGGCGCGTCA 13620
Dwarf white milo       13561:GTCGAGACCGCGAGGCGCCGAGACCCCGAGGTCTGCTGCACTCTGCTACGGGGCGCGTCA 13620
Tall white sooner milo 13561:GTCGAGACCGCGAGGCGCCGAGACCCCGAGGTCTGCTGCACTCTGCTACGGGGCGCGTCA 13620
bmr-6                  13561:GTCGAGACCGCGAGGCGCCGAGACCCCGAGGTCTGCTGCACTCTGCTACGGGGCGCGTCA 13620
                             ************************************************************

BTx623                 13621:CGGCCCACGAGCACTAGTGAGCAGCCAAGCTAGCCAACCAAACAGGACAGGAGGCTCCGT 13680
Dwarf white milo       13621:CGGCCCACGAGCACTAGTGAGCAGCCAAGCTAGCCAACCAAACAGGACAGGAGGCTCCGT 13680
Tall white sooner milo 13621:CGGCCCACGAGCACTAGTGAGCAGCCAAGCTAGCCAACCAAACAGGACAGGAGGCTCCGT 13680
bmr-6                  13621:CGGCCCACGAGCACTAGTGAGCAGCCAAGCTAGCCAACCAAACAGGACAGGAGGCTCCGT 13680
                             ************************************************************
```

Figure S4 (page19)

```
BTx623                  13681:GCCGCTGCATTCGCTTGACTTGGGGCGACGACCGCGTCACAGAATTGGTCATTCATGGCT 13740
Dwarf white milo        13681:GCCGCTGCATTCGCTTGACTTGGGGCGACGACCGCGTCACAGAATTGGTCATTCATGGCT 13740
Tall white sooner milo  13681:GCCGCTGCATTCGCTTGACTTGGGGCGACGACCGCGTCACAGAATTGGTCATTCATGGCT 13740
bmr-6                   13681:GCCGCTGCATTCGCTTGACTTGGGGCGACGACCGCGTCACAGAATTGGTCATTCATGGCT 13740
                              ************************************************************

BTx623                  13741:GTTTCTTGCCCGTTGGCAGTAGAGCCAGCCAGGTAAAGATAACTGGAGTCTACACCGTCA 13800
Dwarf white milo        13741:GTTTCTTGCCCGTTGGCAGTAGAGCCAGCCAGGTAAAGATAACTGGAGTCTACACCGTCA 13800
Tall white sooner milo  13741:GTTTCTTGCCCGTTGGCAGTAGAGCCAGCCAGGTAAAGATAACTGGAGTCTACACCGTCA 13800
bmr-6                   13741:GTTTCTTGCCCGTTGGCAGTAGAGCCAGCCAGGTAAAGATAACTGGAGTCTACACCGTCA 13800
                              ************************************************************

BTx623                  13801:AAACTCTTTTGTTTGCCACACATCCTGTTGCGTAGATTGGCTGCATATATTTGTTGGTAG 13860
Dwarf white milo        13801:AAACTCTTTTGTTTGCCACACATCCTGTTGCGTAGATTGGCTGCATATATTTGTTGGTAG 13860
Tall white sooner milo  13801:AAACTCTTTTGTTTGCCACACATCCTGTTGCGTAGATTGGCTGCATATATTTGTTGGTAG 13860
bmr-6                   13801:AAACTCTTTTGTTTGCCACACATCCTGTTGCGTAGATTGGCTGCATATATTTGTTGGTAG 13860
                              ************************************************************

BTx623                  13861:TTGGTGAGGCTTATAATATATGATCGATCATTGTCACGGTATGGATAGAAATGGAGAGGA 13920
Dwarf white milo        13861:TTGGTGAGGCTTATAATATATGATCGATCATTGTCACGGTATGGATAGAAATGGAGAGGA 13920
Tall white sooner milo  13861:TTGGTGAGGCTTATAATATATGATCGATCATTGTCACGGTATGGATAGAAATGGAGAGGA 13920
bmr-6                   13861:TTGGTGAGGCTTATAATATATGATCGATCATTGTCACGGTATGGATAGAAATGGAGAGGA 13920
                              ************************************************************

BTx623                  13921:AGGATTCACTGGCTTGCAGTCTTGCACAAGACAAAGGCGATACTGACTAAACGTGAATGT 13980
Dwarf white milo        13921:AGGATTCACTGGCTTGCAGTCTTGCACAAGACAAAGGCGATACTGACTAAACGTGAATGT 13980
Tall white sooner milo  13921:AGGATTCACTGGCTTGCAGTCTTGCACAAGACAAAGGCGATACTGACTAAACGTGAATGT 13980
bmr-6                   13921:AGGATTCACTGGCTTGCAGTCTTGCACAAGACAAAGGCGATACTGACTAAACGTGAATGT 13980
                              ************************************************************

BTx623                  13981:TAGATAAGTATTCCAGCGATGACAGCGACAAACGCGAGGCATTGACTTGGAGCAGTGCTA 14040
Dwarf white milo        13981:TAGATAAGTATTCCAGCGATGACAGCGACAAACGCGAGGCATTGACTTGGAGCAGTGCTA 14040
Tall white sooner milo  13981:TAGATAAGTATTCCAGCGATGACAGCGACAAACGCGAGGCATTGACTTGGAGCAGTGCTA 14040
bmr-6                   13981:TAGATAAGTATTCCAGCGATGACAGCGACAAACGCGAGGCATTGACTTGGAGCAGTGCTA 14040
                              ************************************************************

BTx623                  14041:TCTCCACAGGCGACTGGTTCAACTTCAAGCAGGCTGATGATAGATAGTGGCCTTGTTTAC 14100
Dwarf white milo        14041:TCTCCACAGGCGACTGGTTCAACTTCAAGCAGGCTGATGATAGATAGTGGCCTTGTTTAC 14100
Tall white sooner milo  14041:TCTCCACAGGCGACTGGTTCAACTTCAAGCAGGCTGATGATAGATAGTGGCCTTGTTTAC 14100
bmr-6                   14041:TCTCCACAGGCGACTGGTTCAACTTCAAGCAGGCTGATGATAGATAGTGGCCTTGTTTAC 14100
                              ************************************************************

BTx623                  14101:TTCCATCCAAAATCCGAAAAAAAAATTCAAGATTTTTCATTATATCGAATCTTTGAATAC 14160
Dwarf white milo        14101:TTCCATCCAAAATCCGAAAAAAAAATTCAAGATTTTTCATTATATCGAATCTTTGAATAC 14160
Tall white sooner milo  14101:TTCCATCCAAAATCCGAAAAAAAAATTCAAGATTTTTCATTATATCGAATCTTTGAATAC 14160
bmr-6                   14101:TTCCATCCAAAATCCGAAAAAAAAATTCAAGATTTTTCATTATATCGAATCTTTGAATAC 14160
                              ************************************************************

BTx623                  14161:ATATATGGAGCATTAAATATAGACAAAATAAAAACTAATTATACAGTTTATTTGTAATTC 14220
Dwarf white milo        14161:ATATATGGAGCATTAAATATAGACAAAATAAAAACTAATTATACAGTTTATTTGTAATTC 14220
Tall white sooner milo  14161:ATATATGGAGCATTAAATATAGACAAAATAAAAACTAATTATACAGTTTATTTGTAATTC 14220
bmr-6                   14161:ATATATGGAGCATTAAATATAGACAAAATAAAAACTAATTATACAGTTTATTTGTAATTC 14220
                              ************************************************************

BTx623                  14221:ACGGGATGAATCTTTTAAGCCTATTTAGTCCATAATTAGATAATAACTTTCAAATACAAA 14280
Dwarf white milo        14221:ACGGGATGAATCTTTTAAGCCTATTTAGTCCATAATTAGATAATAACTTTCAAATACAAA 14280
Tall white sooner milo  14221:ACGGGATGAATCTTTTAAGCCTATTTAGTCCATAATTAGATAATAACTTTCAAATACAAA 14280
bmr-6                   14221:ACGGGATGAATCTTTTAAGCCTATTTAGTCCATAATTAGATAATAACTTTCAAATACAAA 14280
                              ************************************************************

BTx623                  14281:TGTAAGTGCTACAGTGTCTCGAAAAATTCGAGAAGAAACTAAACATGGCCACACTATGGC 14340
Dwarf white milo        14281:TGTAAGTGCTACAGTGTCTCGAAAAATTCGAGAAGAAACTAAACATGGCCACACTATGGC 14340
Tall white sooner milo  14281:TGTAAGTGCTACAGTGTCTCGAAAAATTCGAGAAGAAACTAAACATGGCCACACTATGGC 14340
bmr-6                   14281:TGTAAGTGCTACAGTGTCTCGAAAAATTCGAGAAGAAACTAAACATGGCCACACTATGGC 14340
                              ************************************************************

BTx623                  14341:CCTGTTTGCTTCAACTTATCTACCGAATCTACTAGTCATTCATTAGTGTTTTTCTCTCAT 14400
Dwarf white milo        14341:CCTGTTTGCTTCAACTTATCTACCGAATCTACTAGTCATTCATTAGTGTTTTTCTCTCAT 14400
Tall white sooner milo  14341:CCTGTTTGCTTCAACTTATCTACCGAATCTACTAGTCATTCATTAGTGTTTTTCTCTCAT 14400
bmr-6                   14341:CCTGTTTGCTTCAACTTATCTACCGAATCTACTAGTCATTCATTAGTGTTTTTCTCTCAT 14400
                              ************************************************************
```

Figure S4 (page20)

```
BTx623                  14401:AATAAATCAACGAATAAAACTTTTAGCCAATTTTTCAGACATGTTGTAAAGGAATCTTAA 14460
Dwarf white milo        14401:AATAAATCAACGAATAAAACTTTTAGCCAATTTTTCAGACATGTTGTAAAGGAATCTTAA 14460
Tall white sooner milo  14401:AATAAATCAACGAATAAAACTTTTAGCCAATTTTTCAGACATGTTGTAAAGGAATCTTAA 14460
bmr-6                   14401:AATAAATCAACGAATAAAACTTTTAGCCAATTTTTCAGACATGTTGTAAAGGAATCTTAA 14460
                              ************************************************************

BTx623                  14461:TTGTTCATTCATGTGTGCATATGGACATAGTGGTGGAACTTTAGTCTCATTTTAGCCTTG 14520
Dwarf white milo        14461:TTGTTCATTCATGTGTGCATATGGACATAGTGGTGGAACTTTAGTCTCATTTTAGCCTTG 14520
Tall white sooner milo  14461:TTGTTCATTCATGTGTGCATATGGACATAGTGGTGGAACTTTAGTCTCATTTTAGCCTTG 14520
bmr-6                   14461:TTGTTCATTCATGTGTGCATATGGACATAGTGGTGGAACTTTAGTCTCATTTTAGCCTTG 14520
                              ************************************************************

BTx623                  14521:TTTAGTTCATTATCAGATTTTTTTTGGCTAATGTAGCACTTTCGTTTGTATTTGACAATT 14580
Dwarf white milo        14521:TTTAGTTCATTATCAGATTTTTTTTGGCTAATGTAGCACTTTCGTTTGTATTTGACAATT 14580
Tall white sooner milo  14521:TTTAGTTCATTATCAGATTTTTTTTGGCTAATGTAGCACTTTCGTTTGTATTTGACAATT 14580
bmr-6                   14521:TTTAGTTCATTATCAGATTTTTTTTGGCTAATGTAGCACTTTCGTTTGTATTTGACAATT 14580
                              ************************************************************

BTx623                  14581:ATTGTTCAATCATGAATTAACTAGGTTTAAAAGTTTCGTCTCACAAATTGCAGGTAAACT 14640
Dwarf white milo        14581:ATTGTTCAATCATGAATTAACTAGGTTTAAAAGTTTCGTCTCACAAATTGCAGGTAAACT 14640
Tall white sooner milo  14581:ATTGTTCAATCATGAATTAACTAGGTTTAAAAGTTTCGTCTCACAAATTGCAGGTAAACT 14640
bmr-6                   14581:ATTGTTCAATCATGAATTAACTAGGTTTAAAAGTTTCGTCTCACAAATTGCAGGTAAACT 14640
                              ************************************************************

BTx623                  14641:ATGCAATTAGTTATTTTTTATCTATATTTAAAGCTCTATGCATATGCAACAAGATTTGAT 14700
Dwarf white milo        14641:ATGCAATTAGTTATTTTTTATCTATATTTAAAGCTCTATGCATATGCAACAAGATTTGAT 14700
Tall white sooner milo  14641:ATGCAATTAGTTATTTTTTATCTATATTTAAAGCTCTATGCATATGCAACAAGATTTGAT 14700
bmr-6                   14641:ATGCAATTAGTTATTTTTTATCTATATTTAAAGCTCTATGCATATGCAACAAGATTTGAT 14700
                              ************************************************************

BTx623                  14701:GTGATGAATTTTTTTTAAAAAAATATTAGATTTTAGGTGAAACTAAACAAGTCCTTAGTA 14760
Dwarf white milo        14701:GTGATGAATTTTTTTTAAAAAAATATTAGATTTTAGGTGAAACTAAACAAGTCCTTAGTA 14760
Tall white sooner milo  14701:GTGATGAATTTTTTTAAAAAAAATATTAGATTTTAGGTGAAACTAAACAAGTCCTTAGTA 14760
bmr-6                   14701:GTGATGAATTTTTTTTAAAAAAATATTAGATTTTAGGTGAAACTAAACAAGTCCTTAGTA 14760
                              ***************.********************************************

BTx623                  14761:GTTGAAGTGGATGATGTCATTTTTAGATATGAAAGCCCTTTGCTCTCCGCTTCACTATAT 14820
Dwarf white milo        14761:GTTGAAGTGGATGATGTCATTTTTAGATATGAAAGCCCTTTGCTCTCCGCTTCACTATAT 14820
Tall white sooner milo  14761:GTTGAAGTGGATGATGTCATTTTTAGATATGAAAGCCCTTTGCTCTCCGCTTCACTATAT 14820
bmr-6                   14761:GTTGAAGTGGATGATGTCATTTTTAGATATGAAAGCCCTTTGCTCTCCGCTTCACTATAT 14820
                              ************************************************************

BTx623                  14821:CTAATGTGAGAAGAGAGAAAGAAACTCCATGCGTGTGTGCTCGCGATGACAGCCGTGTGG 14880
Dwarf white milo        14821:CTAATGTGAGAAGAGAGAAAGAAACTCCATGCGTGTGTGCTCGCGATGACAGCCGTGTGG 14880
Tall white sooner milo  14821:CTAATGTGAGAAGAGAGAAAGAAACTCCATGCGTGTGTGCTCGCGATGACAGCCGTGTGG 14880
bmr-6                   14821:CTAATGTGAGAAGAGAGAAAGAAACTCCATGCGTGTGTGCTCGCGATGACAGCCGTGTGG 14880
                              ************************************************************

BTx623                  14881:CAGGGTCAGAGCGTGAAAGAAGAGGGAGCCTGACAAAGCCCTTTTTATATAAGTTAATTA 14940
Dwarf white milo        14881:CAGGGTCAGAGCGTGAAAGAAGAGGGAGCCTGACAAAGCCCTTTTTATATAAGTTAATTA 14940
Tall white sooner milo  14881:CAGGGTCAGAGCGTGAAAGAAGAGGGAGCCTGACAAAGCCCTTTTTATATAAGTTAATTA 14940
bmr-6                   14881:CAGGGTCAGAGCGTGAAAGAAGAGGGAGCCTGACAAAGCCCTTTTTATATAAGTTAATTA 14940
                              ************************************************************

BTx623                  14941:CGAGAAATTATTCAAGACATACGTTTTCTCTTTCTCTACTTTTTTTAGTCGTTTCAAATA 15000
Dwarf white milo        14941:CGAGAAATTATTCAAGACATACGTTTTCTCTTTCTCTACTTTTTTTAGTCGTTTCAAATA 15000
Tall white sooner milo  14941:CGAGAAATTATTCAAGACATACGTTTTCTCTTTCTCTACTTTTTTTAGTCGTTTCAAATA 15000
bmr-6                   14941:CGAGAAATTATTCAAGACATACGTTTTCTCTTTCTCTACTTTTTTTAGTCGTTTCAAATA 15000
                              ************************************************************

BTx623                  15001:CTTACTGATTTATCAAGTGTACGGCCAGAGTGTGAAAGAAGAGGAAGCCTGACAAAGCCC 15060
Dwarf white milo        15001:CTTACTGATTTATCAAGTGTACGGCCAGAGTGTGAAAGAAGAGGAAGCCTGACAAAGCCC 15060
Tall white sooner milo  15001:CTTACTGATTTATCAAGTGTACGGCCAGAGTGTGAAAGAAGAGGAAGCCTGACAAAGCCC 15060
bmr-6                   15001:CTTACTGATTTATCAAGTGTACGGCCAGAGTGTGAAAGAAGAGGAAGCCTGACAAAGCCC 15060
                              ************************************************************

BTx623                  15061:TTTTTTATATCTAAGTTAATTACGAGAAATCACTCGAGACACACGTTTTCTCTTTCTTTA 15120
Dwarf white milo        15061:TTTTTTATATCTAAGTTAATTACGAGAAATCACTCGAGACACACGTTTTCTCTTTCTTTA 15120
Tall white sooner milo  15061:TTTTTTATATCTAAGTTAATTACGAGAAATCACTCGAGACACACGTTTTCTCTTTCTTTA 15120
bmr-6                   15061:TTTTTTATATCTAAGTTAATTACGAGAAATCACTCGAGACACACGTTTTCTCTTTCTTTA 15120
                              ************************************************************
```

```
BTx623               15121:ATTTTTTTAGCTGTTTCAAATACTTGATTTATCAAGTGTGTTTTTAGAGAGGTGCTTCAA 15180
Dwarf white milo     15121:ATTTTTTTAGCTGTTTCAAATACTTGATTTATCAAGTGTGTTTTTAGAGAGGTGCTTCAA 15180
Tall white sooner milo 15121:ATTTTTTTAGCTGTTTCAAATACTTGATTTATCAAGTGTGTTTTTAGAGAGGTGCTTCAA 15180
bmr-6                15121:ATTTTTTTAGCTGTTTCAAATACTTGATTTATCAAGTGTGTTTTTAGAGAGGTGCTTCAA 15180
                           ************************************************************

BTx623               15181:TGAAAGTGTGAAAGCACCCGCTGCGCCCTTTGTTTAGGGTAGGTTGCTTATTCTTATTTG 15240
Dwarf white milo     15181:TGAAAGTGTGAAAGCACCCGCTGCGCCCTTTGTTTAGGGTAGGTTGCTTATTCTTATTTG 15240
Tall white sooner milo 15181:TGAAAGTGTGAAAGCACCCGCTGCGCCCTTTGTTTAGGGTAGGTTGCTTATTCTTATTTG 15240
bmr-6                15181:TGAAAGTGTGAAAGCACCCGCTGCGCCCTTTGTTTAGGGTAGGTTGCTTATTCTTATTTG 15240
                           ************************************************************

BTx623               15241:TGTACTTCGTCCACTTTAAATTATAAATTCCTTTGGTTTTGTTAGATAGATAGATTTTAC 15300
Dwarf white milo     15241:TGTACTTCGTCCACTTTAAATTATAAATTCCTTTGGTTTTGTTAGATAGATAGATTTTAC 15300
Tall white sooner milo 15241:TGTACTTCGTCCACTTTAAATTATAAATTCCTTTGGTTTTGTTAGATAGATAGATTTTAC 15300
bmr-6                15241:TGTACTTCGTCCACTTTAAATTATAAATTCCTTTGGTTTTGTTAGATAGATAGATTTTAC 15300
                           ************************************************************

BTx623               15301:TATATATTTAGATATAGATTTTTTTAAAGAGCAACAAGAGGTTTGCCATGGTTTTATTGA 15360
Dwarf white milo     15301:TATATATTTAGATATAGATTTTTTTAAAGAGCAACAAGAGGTTTGCCATGGTTTTATTGA 15360
Tall white sooner milo 15301:TATATATTTAGATATAGATTTTTTTAAAGAGCAACAAGAGGTTTGCCATGGTTTTATTGA 15360
bmr-6                15301:TATATATTTAGATATAGATTTTTTTAAAGAGCAACAAGAGGTTTGCCATGGTTTTATTGA 15360
                           ************************************************************

BTx623               15361:CGATAAAAAGAGCAAAAACATGATTACAAATGCATGACTAGAGTAACACCGGTGGTCCAC 15420
Dwarf white milo     15361:CGATAAAAAGAGCAAAAACATGATTACAAATGCATGACTAGAGTAACACCGGTGGTCCAC 15420
Tall white sooner milo 15361:CGATAAAAAGAGCAAAAACATGATTACAAATGCATGACTAGAGTAACACCGGTGGTCCAC 15420
bmr-6                15361:CGATAAAAAGAGCAAAAACATGATTACAAATGCATGACTAGAGTAACACCGGTGGTCCAC 15420
                           ************************************************************

BTx623               15421:AACTACCAATCCTGATGTGACAAAAAGAAAAAGAGAGAGAGAGAAAAAAAAAGACACAACA 15480
Dwarf white milo     15421:AACTACCAATCCTGATGTGACAAAAAGAAAAAGAGAGAGAGAGAAAAAAAAAGACACAACA 15480
Tall white sooner milo 15421:AACTACCAATCCTGATGTGACAAAAAGAAAAAGAGAGAGAGAGAAAAAAAAAGACACAACA 15480
bmr-6                15421:AACTACCAATCCTGATGTGACAAAAAGAAAAAGAGAGAGAGAGAAAAAAAAAGACACAACA 15480
                           ************************************************************

BTx623               15481:GGTCAGAAAAAAAAGGTCATAAAAAGTACCTCCAAAAAATCCAAGAGGAATGGCACAACAC 15540
Dwarf white milo     15481:GGTCAGAAAAAAAAGGTCATAAAAAGTACCTCCAAAAAATCCAAGAGGAATGGCACAACAC 15540
Tall white sooner milo 15481:GGTCAGAAAAAAAAGGTCATAAAAAGTACCTCCAAAAAATCCAAGAGGAATGGCACAACAC 15540
bmr-6                15481:GGTCAGAAAAAAAAGGTCATAAAAAGTACCTCCAAAAAATCCAAGAGGAATGGCACAACAC 15540
                           ************************************************************

BTx623               15541:AATGACTAAACGCAACTAAAAACTTAGGAAGACAACTTTAAAAGAATCTGTGGTAGTATT 15600
Dwarf white milo     15541:AATGACTAAACGCAACTAAAAACTTAGGAAGACAACTTTAAAAGAATCTGTGGTAGTATT 15600
Tall white sooner milo 15541:AATGACTAAACGCAACTAAAAACTTAGGAAGACAACTTTAAAAGAATCTGTGGTAGTATT 15600
bmr-6                15541:AATGACTAAACGCAACTAAAAACTTAGGAAGACAACTTTAAAAGAATCTGTGGTAGTATT 15600
                           ************************************************************

BTx623               15601:TGTTATAGTGCCAAGTTAAACTGTTGTATATCATGCTTGCACAAAGAGAGTAGCTAGTTA 15660
Dwarf white milo     15601:TGTTATAGTGCCAAGTTAAACTGTTGTATATCATGCTTGCACAAAGAGAGTAGCTAGTTA 15660
Tall white sooner milo 15601:TGTTATAGTGCCAAGTTAAACTGTTGTATATCATGCTTGCACAAAGAGAGTAGCTAGTTA 15660
bmr-6                15601:TGTTATAGTGCCAAGTTAAACTGTTGTATATCATGCTTGCACAAAGAGAGTAGCTAGTTA 15660
                           ************************************************************

BTx623               15661:GATTCGATGTCACGAAAGAATATTTAAAATTTTTTGGAATTGGTGCAACTAAAAGGCCTT 15720
Dwarf white milo     15661:GATTCGATGTCACGAAAGAATATTTAAAATTTTTTGGAATTGGTGCAACTAAAAGGCCTT 15720
Tall white sooner milo 15661:GATTCGATGTCACGAAAGAATATTTAAAATTTTTTGGAATTGGTGCAACTAAAAGGCCTT 15720
bmr-6                15661:GATTCGATGTCACGAAAGAATATTTAAAATTTTTTGGAATTGGTGCAACTAAAAGGCCTT 15720
                           ************************************************************

BTx623               15721:ATTTCTGTTTCAATCATCGGCCCAGCTTTACTGAGATGCCAATCTCGGGCATCCTCCATT 15780
Dwarf white milo     15721:ATTTCTGTTTCAATCATCGGCCCAGCTTTACTGAGATGCCAATCTCGGGCATCCTCCATT 15780
Tall white sooner milo 15721:ATTTCTGTTTCAATCATCGGCCCAGCTTTACTGAGATGCCAATCTCGGGCATCCTCCATT 15780
bmr-6                15721:ATTTCTGTTTCAATCATCGGCCCAGCTTTACTGAGATGCCAATCTCGGGCATCCTCCATT 15780
                           ************************************************************

BTx623               15781:TTACATGGGTTGGACCACTTGGACGGAGTAACGACCACGTGGTTCTCGCTATATTTGAAC 15840
Dwarf white milo     15781:TTACATGGGTTGGACCACTTGGACGGAGTAACGACCACGTGGTTCTCGCTATATTTGAAC 15840
Tall white sooner milo 15781:TTACATGGGTTGGACCACTTGGACGGAGTAACGACCACGTGGTTCTCGCTATATTTGAAC 15840
bmr-6                15781:TTACATGGGTTGGACCACTTGGACGGAGTAACGACCACGTGGTTCTCGCTATATTTGAAC 15840
                           ************************************************************
```

Figure S4 (page22)

```
BTx623                  15841:GCATGCCTTCCCGCGCGAATCGCGCACCGGCAGCGCGAAGGCGCCAGCTATGCACAGCGA 15900
Dwarf white milo        15841:GCATGCCTTCCCGCGCGAATCGCGCACCGGCAGCGCGAAGGCGCCAGCTATGCACAGCGA 15900
Tall white sooner milo  15841:GCATGCCTTCCCGCGCGAATCGCGCACCGGCAGCGCGAAGGCGCCAGCTATGCACAGCGA 15900
bmr-6                   15841:GCATGCCTTCCCGCGCGAATCGCGCACCGGCAGCGCGAAGGCGCCAGCTATGCACAGCGA 15900
                              ************************************************************

BTx623                  15901:ATGGAGCTAGAAACCGCCGGCGGCGAGCGCGCGCGACGAAGACGATCCAAATTTAGGCGC 15960
Dwarf white milo        15901:ATGGAGCTAGAAACCGCCGGCGGCGAGCGCGCGCGACGAAGACGATCCAAATTTAGGCGC 15960
Tall white sooner milo  15901:ATGGAGCTAGAAACCGCCGGCGGCGAGCGCGCGCGACGAAGACGATCCAAATTTAGGCGC 15960
bmr-6                   15901:ATGGAGCTAGAAACCGCCGGCGGCGAGCGCGCGCGACGAAGACGATCCAAATTTAGGCGC 15960
                              ************************************************************

BTx623                  15961:CAGAATGACTAGAGGAGCGGTCACGCAGGAGGAGGCCGGAGGGAAGGGGGAGACCGGCTC 16020
Dwarf white milo        15961:CAGAATGACTAGAGGAGCGGTCACGCAGGAGGAGGCCGGAGGGAAGGGGGAGACCGGCTC 16020
Tall white sooner milo  15961:CAGAATGACTAGAGGAGCGGTCACGCAGGAGGAGGCCGGAGGGAAGGGGGAGACCGGCTC 16020
bmr-6                   15961:CAGAATGACTAGAGGAGCGGTCACGCAGGAGGAGGCCGGAGGGAAGGGGGAGACCGGCTC 16020
                              ************************************************************

BTx623                  16021:CTCGACCTCTCCGCCGCCGCCGCCGCCGCAATGGGGTCCCCACCCCGCTCTGCGGTCATC 16080
Dwarf white milo        16021:CTCGACCTCTCCGCCGCCGCCGCCGCCGCAATGGGGTCCCCACCCCGCTCTGCGGTCATC 16080
Tall white sooner milo  16021:CTCGACCTCTCCGCCGCCGCCGCCGCCGCAATGGGGTCCCCACCCCGCTCTGCGGTCATC 16080
bmr-6                   16021:CTCGACCTCTCCGCCGCCGCCGCCGCCGCAATGGGGTCCCCACCCCGCTCTGCGGTCATC 16080
                              ************************************************************

BTx623                  16081:GCCGTGGCCTTCCTCCTCCTCGCCTCCTCCGGTGAGGCGTTCTTCGACATCTTCAACTTC 16140
Dwarf white milo        16081:GCCGTGGCCTTCCTCCTCCTCGCCTCCTCCGGTGAGGCGTTCTTCGACATCTTCAACTTC 16140
Tall white sooner milo  16081:GCCGTGGCCTTCCTCCTCCTCGCCTCCTCCGGTGAGGCGTTCTTCGACATCTTCAACTTC 16140
bmr-6                   16081:GCCGTGGCCTTCCTCCTCCTCGCCTCCTCCGGTGAGGCGTTCTTCGACATCTTCAACTTC 16140
                              ************************************************************

BTx623                  16141:TTCCACCCGCACTCGGAGACCGACTACTTCCAGAACGCCTTCGAGGGCACGCCGGAGCAG 16200
Dwarf white milo        16141:TTCCACCCGCACTCGGAGACCGACTACTTCCAGAACGCCTTCGAGGGCACGCCGGAGCAG 16200
Tall white sooner milo  16141:TTCCACCCGCACTCGGAGACCGACTACTTCCAGAACGCCTTCGAGGGCACGCCGGAGCAG 16200
bmr-6                   16141:TTCCACCCGCACTCGGAGACCGACTACTTCCAGAACGCCTTCGAGGGCACGCCGGAGCAG 16200
                              ************************************************************

BTx623                  16201:ACGGTGCCCACGCAGACGGAGCAGGAGGAGCGGGGCGCCGCGCCGGCCCTCGCCACGGGC 16260
Dwarf white milo        16201:ACGGTGCCCACGCAGACGGAGCAGGAGGAGCGGGGCGCCGCGCCGGCCCTCGCCACGGGC 16260
Tall white sooner milo  16201:ACGGTGCCCACGCAGACGGAGCAGGAGGAGCGGGGCGCCGCGCCGGCCCTCGCCACGGGC 16260
bmr-6                   16201:ACGGTGCCCACGCAGACGGAGCAGGAGGAGCGGGGCGCCGCGCCGGCCCTCGCCACGGGC 16260
                              ************************************************************

BTx623                  16261:CTCACCAGGGTGCCGGCCGGGGGACCGCCCAGCAAGGCTGCGCAGGACACGGTCGCGCTC 16320
Dwarf white milo        16261:CTCACCAGGGTGCCGGCCGGGGGACCGCCCAGCAAGGCTGCGCAGGACACGGTCGCGCTC 16320
Tall white sooner milo  16261:CTCACCAGGGTGCCGGCCGGGGGACCGCCCAGCAAGGCTGCGCAGGACACGGTCGCGCTC 16320
bmr-6                   16261:CTCACCAGGGTGCCGGCCGGGGGACCGCCCAGCAAGGCTGCGCAGGACACGGTCGCGCTC 16320
                              ************************************************************

BTx623                  16321:CCCGCCGACAAAGGCGGCGGGGCAGTCGGCTCGTGGACCATCGTCAGCGAAAACTCCGGC 16380
Dwarf white milo        16321:CCCGCCGACAAAGGCGGCGGGGCAGTCGGCTCGTGGACCATCGTCAGCGAAAACTCCGGC 16380
Tall white sooner milo  16321:CCCGCCGACAAAGGCGGCGGGGCAGTCGGCTCGTGGACCATCGTCAGCGAAAACTCCGGC 16380
bmr-6                   16321:CCCGCCGACAAAGGCGGCGGGGCAGTCGGCTCGTGGACCATCGTCAGCGAAAACTCCGGC 16380
                              ************************************************************

BTx623                  16381:GTGTCGGCCATGCACATGGTCGTTATGCGCCATGGCAAGGCCGTCATGTTCGATACCAGC 16440
Dwarf white milo        16381:GTGTCGGCCATGCACATGGTCGTTATGCGCCATGGCAAGGCCGTCATGTTCGATACCAGC 16440
Tall white sooner milo  16381:GTGTCGGCCATGCACATGGTCGTTATGCGCCATGGCAAGGCCGTCATGTTCGATACCAGC 16440
bmr-6                   16381:GTGTCGGCCATGCACATGGTCGTTATGCGCCATGGCAAGGCCGTCATGTTCGATACCAGC 16440
                              ************************************************************

BTx623                  16441:ACCACCGGGCGGTCACTCATGCGGTTGCCCCAGGACAACTGCCGCATCGACCCGCGCGCC 16500
Dwarf white milo        16441:ACCACCGGGCGGTCACTCATGCGGTTGCCCCAGGACAACTGCCGCATCGACCCGCGCGCC 16500
Tall white sooner milo  16441:ACCACCGGGCGGTCACTCATGCGGTTGCCCCAGGACAACTGCCGCATCGACCCGCGCGCC 16500
bmr-6                   16441:ACCACCGGGCGGTCACTCATGCGGTTGCCCCAGGACAACTGCCGCATCGACCCGCGCGCC 16500
                              ************************************************************

BTx623                  16501:AAGGAGGAGGGCACCATGGACTGCTGGGCGCACTCCGTCGAGTTCGACTACAACACCGGC 16560
Dwarf white milo        16501:AAGGAGGAGGGCACCATGGACTGCTGGGCGCACTCCGTCGAGTTCGACTACAACACCGGC 16560
Tall white sooner milo  16501:AAGGAGGAGGGCACCATGGACTGCTGGGCGCACTCCGTCGAGTTCGACTACAACACCGGC 16560
bmr-6                   16501:AAGGAGGAGGGCACCATGGACTGCTGGGCGCACTCCGTCGAGTTCGACTACAACACCGGC 16560
                              ************************************************************
```

Figure S4 (page23)

```
BTx623                 16561:GGCCTCCGCCCTCTCAAGGTTATTGGCTGCCTTAACGTTCTTGCATGCATGTGCAATGGC 16620
Dwarf white milo       16561:GGCCTCCGCCCTCTCAAGGTTATTGGCTGCCTTAACGTTCTTGCATGCATGTGCAATGGC 16620
Tall white sooner milo 16561:GGCCTCCGCCCTCTCAAGGTTATTGGCTGCCTTAACGTTCTTGCATGCATGTGCAATGGC 16620
bmr-6                  16561:GGCCTCCGCCCTCTCAAGGTTATTGGCTGCCTTAACGTTCTTGCATGCATGTGCAATGGC 16620
                             ************************************************************

BTx623                 16621:TCCGGTCCCAGTCTCAGGCCGGTTTTGATGGCATGCAATGCAATGCAATGCGCAGATCTT 16680
Dwarf white milo       16621:TCCGGTCCCAGTCTCAGGCCGGTTTTGATGGCATGCAATGCAATGCAATGCGCAGATCTT 16680
Tall white sooner milo 16621:TCCGGTCCCAGTCTCAGGCCGGTTTTGATGGCATGCAATGCAATGCAATGCGCAGATCTT 16680
bmr-6                  16621:TCCGGTCCCAGTCTCAGGCCGGTTTTGATGGCATGCAATGCAATGCAATGCGCAGATCTT 16680
                             ************************************************************

BTx623                 16681:AACAGACACATGGTGCTCGTCGGGCGCGTTGGACCCGGACGGCAACCTGGTGCAGACCGG 16740
Dwarf white milo       16681:AACAGACACATGGTGCTCGTCGGGCGCGTTGGACCCGGACGGCAACCTGGTGCAGACCGG 16740
Tall white sooner milo 16681:AACAGACACATGGTGCTCGTCGGGCGCGTTGGACCCGGACGGCAACCTGGTGCAGACCGG 16740
bmr-6                  16681:AACAGACACATGGTGCTCGTCGGGCGCGTTGGACCCGGACGGCAACCTGGTGCAGACCGG 16740
                             ************************************************************

BTx623                 16741:CGGCTACTTCGAGGGCGAGAAGGTTGTGAGGACCCTGAGCCCGTGCGACACCTGCGACTG 16800
Dwarf white milo       16741:CGGCTACTTCGAGGGCGAGAAGGTTGTGAGGACCCTGAGCCCGTGCGACACCTGCGACTG 16800
Tall white sooner milo 16741:CGGCTACTTCGAGGGCGAGAAGGTTGTGAGGACCCTGAGCCCGTGCGACACCTGCGACTG 16800
bmr-6                  16741:CGGCTACTTCGAGGGCGAGAAGGTTGTGAGGACCCTGAGCCCGTGCGACACCTGCGACTG 16800
                             ************************************************************

BTx623                 16801:GCTGGAGCAACCCAACAGCTTCGCCGAGGGGAGATGGTACGCGACGCAGGTAGCGCTCCC 16860
Dwarf white milo       16801:GCTGGAGCAACCCAACAGCTTCGCCGAGGGGAGATGGTACGCGACGCAGGTAGCGCTCCC 16860
Tall white sooner milo 16801:GCTGGAGCAACCCAACAGCTTCGCCGAGGGGAGATGGTACGCGACGCAGGTAGCGCTCCC 16860
bmr-6                  16801:GCTGGAGCAACCCAACAGCTTCGCCGAGGGGAGATGGTACGCGACGCAGGTAGCGCTCCC 16860
                             ************************************************************

BTx623                 16861:GGACGGCCGGTTCATCATGTTCGGCGGTCGCCGCGCCTTCAGCTACGAGTACGTCCCGTG 16920
Dwarf white milo       16861:GGACGGCCGGTTCATCATGTTCGGCGGTCGCCGCGCCTTCAGCTACGAGTACGTCCCGTG 16920
Tall white sooner milo 16861:GGACGGCCGGTTCATCATGTTCGGCGGTCGCCGCGCCTTCAGCTACGAGTACGTCCCGTG 16920
bmr-6                  16861:GGACGGCCGGTTCATCATGTTCGGCGGTCGCCGCGCCTTCAGCTACGAGTACGTCCCGTG 16920
                             ************************************************************

BTx623                 16921:GCCCGGGAAGTCCAACGACAAGGCCGTCCGGTTGCCCTTCTTCCGCGAGACCACCGACGA 16980
Dwarf white milo       16921:GCCCGGGAAGTCCAACGACAAGGCCGTCCGGTTGCCCTTCTTCCGCGAGACCACCGACGA 16980
Tall white sooner milo 16921:GCCCGGGAAGTCCAACGACAAGGCCGTCCGGTTGCCCTTCTTCCGCGAGACCACCGACGA 16980
bmr-6                  16921:GCCCGGGAAGTCCAACGACAAGGCCGTCCGGTTGCCCTTCTTCCGCGAGACCACCGACGA 16980
                             ************************************************************

BTx623                 16981:CGTGGAGAACAACCTGTACCCGTTCGTGAACCTCCTCCCCAGCGGCAACCTGTTCCTCTT 17040
Dwarf white milo       16981:CGTGGAGAACAACCTGTACCCGTTCGTGAACCTCCTCCCCAGCGGCAACCTGTTCCTCTT 17040
Tall white sooner milo 16981:CGTGGAGAACAACCTGTACCCGTTCGTGAACCTCCTCCCCAGCGGCAACCTGTTCCTCTT 17040
bmr-6                  16981:CGTGGAGAACAACCTGTACCCGTTCGTGAACCTCCTCCCCAGCGGCAACCTGTTCCTCTT 17040
                             ************************************************************

BTx623                 17041:CGCCAACGACCGCTCCGTCATCTTCGACGCCAAGTCGAGCAAGATCGTGCGCGAGCTCCC 17100
Dwarf white milo       17041:CGCCAACGACCGCTCCGTCATCTTCGACGCCAAGTCGAGCAAGATCGTGCGCGAGCTCCC 17100
Tall white sooner milo 17041:CGCCAACGACCGCTCCGTCATCTTCGACGCCAAGTCGAGCAAGATCGTGCGCGAGCTCCC 17100
bmr-6                  17041:CGCCAACGACCGCTCCGTCATCTTCGACGCCAAGTCGAGCAAGATCGTGCGCGAGCTCCC 17100
                             ************************************************************

BTx623                 17101:CAAGCTCGACGGCGGGAGCCGCAACTACCCCGGGTCCGCCATGTCGACGCTGCTCCCGCT 17160
Dwarf white milo       17101:CAAGCTCGACGGCGGGAGCCGCAACTACCCCGGGTCCGCCATGTCGACGCTGCTCCCGCT 17160
Tall white sooner milo 17101:CAAGCTCGACGGCGGGAGCCGCAACTACCCCGGGTCCGCCATGTCGACGCTGCTCCCGCT 17160
bmr-6                  17101:CAAGCTCGACGGCGGGAGCCGCAACTACCCCGGGTCCGCCATGTCGACGCTGCTCCCGCT 17160
                             ************************************************************

BTx623                 17161:CGACCTCCGCAACGTCACCGGCGACCCGGAGCCGGTGGTGGTCATCTGCGGCGGGGCGCC 17220
Dwarf white milo       17161:CGACCTCCGCAACGTCACCGGCGACCCGGAGCCGGTGGTGGTCATCTGCGGCGGGGCGCC 17220
Tall white sooner milo 17161:CGACCTCCGCAACGTCACCGGCGACCCGGAGCCGGTGGTGGTCATCTGCGGCGGGGCGCC 17220
bmr-6                  17161:CGACCTCCGCAACGTCACCGGCGACCCGGAGCCGGTGGTGGTCATCTGCGGCGGGGCGCC 17220
                             ************************************************************

BTx623                 17221:CAAGAAGGCCTTCAGGAAAGGCGAGAACAACACGTTCCTGCCGGCGCTCCGCGACTGCGC 17280
Dwarf white milo       17221:CAAGAAGGCCTTCAGGAAAGGCGAGAACAACACGTTCCTGCCGGCGCTCCGCGACTGCGC 17280
Tall white sooner milo 17221:CAAGAAGGCCTTCAGGAAAGGCGAGAACAACACGTTCCTGCCGGCGCTCCGCGACTGCGC 17280
bmr-6                  17221:CAAGAAGGCCTTCAGGAAAGGCGAGAACAACACGTTCCTGCCGGCGCTCCGCGACTGCGC 17280
                             ************************************************************
```

Figure S4 (page24)

```
BTx623               17281:CCGCATCAACCTGGCAAGGCCCGACGCGCAGTGGGAGAGCGAGGACATGCCCGTGGGCCG 17340
Dwarf white milo     17281:CCGCATCAACCTGGCAAGGCCCGACGCGCAGTGGGAGAGCGAGGACATGCCCGTGGGCCG 17340
Tall white sooner milo 17281:CCGCATCAACCTGGCAAGGCCCGACGCGCAGTGGGAGAGCGAGGACATGCCCGTGGGCCG 17340
bmr-6                17281:CCGCATCAACCTGGCAAGGCCCGACGCGCAGTGGGAGAGCGAGGACATGCCCGTGGGCCG 17340
                           ************************************************************

BTx623               17341:CGTCATGGGCGACATGCTGATCCTCCCCACCGGCGACCTGCTGCTGCTCAGCGGCGCCGC 17400
Dwarf white milo     17341:CGTCATGGGCGACATGCTGATCCTCCCCACCGGCGACCTGCTGCTGCTCAGCGGCGCCGC 17400
Tall white sooner milo 17341:CGTCATGGGCGACATGCTGATCCTCCCCACCGGCGACCTGCTGCTGCTCAGCGGCGCCGC 17400
bmr-6                17341:CGTCATGGGCGACATGCTGATCCTCCCCACCGGCGACCTGCTGCTGCTCAGCGGCGCCGC 17400
                           ************************************************************

BTx623               17401:CAAGGGCTGCGCCGGCTGGGGCTTCGGCAGGCAGCCGGTGCTGACCCCGATCCTGTACTC 17460
Dwarf white milo     17401:CAAGGGCTGCGCCGGCTGGGGCTTCGGCAGGCAGCCGGTGCTGACCCCGATCCTGTACTC 17460
Tall white sooner milo 17401:CAAGGGCTGCGCCGGCTGGGGCTTCGGCAGGCAGCCGGTGCTGACCCCGATCCTGTACTC 17460
bmr-6                17401:CAAGGGCTGCGCCGGCTGGGGCTTCGGCAGGCAGCCGGTGCTGACCCCGATCCTGTACTC 17460
                           ************************************************************

BTx623               17461:GCCGCGCAAGGCGGAGGGCCCGCGGTTCCGGGCGCTGGCGTCGTCCACCATCGCGCGCAT 17520
Dwarf white milo     17461:GCCGCGCAAGGCGGAGGGCCCGCGGTTCCGGGCGCTGGCGTCGTCCACCATCGCGCGCAT 17520
Tall white sooner milo 17461:GCCGCGCAAGGCGGAGGGCCCGCGGTTCCGGGCGCTGGCGTCGTCCACCATCGCGCGCAT 17520
bmr-6                17461:GCCGCGCAAGGCGGAGGGCCCGCGGTTCCGGGCGCTGGCGTCGTCCACCATCGCGCGCAT 17520
                           ************************************************************

BTx623               17521:GTACCACTCCAGCAGCGCCGTGCTGCCCGACGCCACCGTGCTGGTGGCCGGCGGCAACGC 17580
Dwarf white milo     17521:GTACCACTCCAGCAGCGCCGTGCTGCCCGACGCCACCGTGCTGGTGGCCGGCGGCAACGC 17580
Tall white sooner milo 17521:GTACCACTCCAGCAGCGCCGTGCTGCCCGACGCCACCGTGCTGGTGGCCGGCGGCAACGC 17580
bmr-6                17521:GTACCACTCCAGCAGCGCCGTGCTGCCCGACGCCACCGTGCTGGTGGCCGGCGGCAACGC 17580
                           ************************************************************

BTx623               17581:CAACGCGGCCTACAACTTCAGCGACGTGGACTTCCCCACCGAGGTGCGCGTGGAGCGGTT 17640
Dwarf white milo     17581:CAACGCGGCCTACAACTTCAGCGACGTGGACTTCCCCACCGAGGTGCGCGTGGAGCGGTT 17640
Tall white sooner milo 17581:CAACGCGGCCTACAACTTCAGCGACGTGGACTTCCCCACCGAGGTGCGCGTGGAGCGGTT 17640
bmr-6                17581:CAACGCGGCCTACAACTTCAGCGACGTGGACTTCCCCACCGAGGTGCGCGTGGAGCGGTT 17640
                           ************************************************************

BTx623               17641:CACCCCGCCGTACCTCAGCGATGATGGGGCCGCCGACAACCGCGCGGTGATCGACTTGGC 17700
Dwarf white milo     17641:CACCCCGCCGTACCTCAGCGATGATGGGGCCGCCGACAACCGCGCGGTGATCGACTTGGC 17700
Tall white sooner milo 17641:CACCCCGCCGTACCTCAGCGATGATGGGGCCGCCGACAACCGCGCGGTGATCGACTTGGC 17700
bmr-6                17641:CACCCCGCCGTACCTCAGCGATGATGGGGCCGCCGACAACCGCGCGGTGATCGACTTGGC 17700
                           ************************************************************

BTx623               17701:GTCGTTGCCCGTGGACGGGATGCGGTACGGCGCCCCGTTCGCGTTCCGGTTCTCCGTGAC 17760
Dwarf white milo     17701:GTCGTTGCCCGTGGACGGGATGCGGTACGGCGCCCCGTTCGCGTTCCGGTTCTCCGTGAC 17760
Tall white sooner milo 17701:GTCGTTGCCCGTGGACGGGATGCGGTACGGCGCCCCGTTCGCGTTCCGGTTCTCCGTGAC 17760
bmr-6                17701:GTCGTTGCCCGTGGACGGGATGCGGTACGGCGCCCCGTTCGCGTTCCGGTTCTCCGTGAC 17760
                           ************************************************************

BTx623               17761:CTCCGAGCCGGCCGTGGTGGAGGCCGACGTGAAGGTCACCTTGTACGCGCCGCCGTTCAC 17820
Dwarf white milo     17761:CTCCGAGCCGGCCGTGGTGGAGGCCGACGTGAAGGTCACCTTGTACGCGCCGCCGTTCAC 17820
Tall white sooner milo 17761:CTCCGAGCCGGCCGTGGTGGAGGCCGACGTGAAGGTCACCTTGTACGCGCCGCCGTTCAC 17820
bmr-6                17761:CTCCGAGCCGGCCGTGGTGGAGGCCGACGTGAAGGTCACCTTGTACGCGCCGCCGTTCAC 17820
                           ************************************************************

BTx623               17821:CACGCACGGCTGCTCCATGAACCAGCGGCTGCTGATCCTGCACTTCACCTCGTACGTCCA 17880
Dwarf white milo     17821:CACGCACGGCTGCTCCATGAACCAGCGGCTGCTGATCCTGCACTTCACCTCGTACGTCCA 17880
Tall white sooner milo 17821:CACGCACGGCTGCTCCATGAACCAGCGGCTGCTGATCCTGCACTTCACCTCGTACGTCCA 17880
bmr-6                17821:CACGCACGGCTGCTCCATGAACCAGCGGCTGCTGATCCTGCACTTCACCTCGTACGTCCA 17880
                           ************************************************************

BTx623               17881:GGAGGGACGGAGCTACAGGGTGTGCGTGGACGGGCCGGGCAAGCCGGAGCTAGCGCCGCG 17940
Dwarf white milo     17881:GGAGGGACGGAGCTACAGGGTGTGCGTGGACGGGCCGGGCAAGCCGGAGCTAGCGCCGCG 17940
Tall white sooner milo 17881:GGAGGGACGGAGCTACAGGGTGTGCGTGGACGGGCCGGGCAAGCCGGAGCTAGCGCCGCG 17940
bmr-6                17881:GGAGGGACGGAGCTACAGGGTGTGCGTGGACGGGCCGGGCAAGCCGGAGCTAGCGCCGCG 17940
                           ************************************************************

BTx623               17941:GGGGTACTACCTGCTGTTCGTCGTGGCCAAGGGCGTGCCGAGCGTCGGTGTGTGGGTGAA 18000
Dwarf white milo     17941:GGGGTACTACCTGCTGTTCGTCGTGGCCAAGGGCGTGCCGAGCGTCGGTGTGTGGGTGAA 18000
Tall white sooner milo 17941:GGGGTACTACCTGCTGTTCGTCGTGGCCAAGGGCGTGCCGAGCGTCGGTGTGTGGGTGAA 18000
bmr-6                17941:GGGGTACTACCTGCTGTTCGTCGTGGCCAAGGGCGTGCCGAGCGTCGGTGTGTGGGTGAA 18000
                           ************************************************************
```

Figure S4 (page25)

```
BTx623                   18001:GGTTTGGTGAGATCGATCGGCCGATCGTTGG                                   18031
Dwarf white milo         18001:GGTTTGGTGAGATCGATCGGCCGATCGTTGG                                   18031
Tall white sooner milo   18001:GGTTTGGTGAGATCGATCGGCCGATCGTTGG                                   18031
bmr-6                    18001:GGTTTGGTGAGATCGATCGGCCGATCGTTGG                                   18031
                               *******************************
```

Supplementary Figure S4 | Sequence comparison of the candidate region among cultivars. Sequences of the candidate region (~18kb) of the four cultivars, BTx623, Dwarf White Milo, Tall White Sooner Milo, and bmr-6 were compared. Asterisk and period indicate the same and different nucleotides, respectively. The nucleotide position at 2057 indicates dw1 mutation (shown as a red arrowhead). Three genomic sequences (Dwarf White Milo, Tall White Sooner Milo, and bmr-6) were determined by resequencing (Illumina Hiseq 2000).

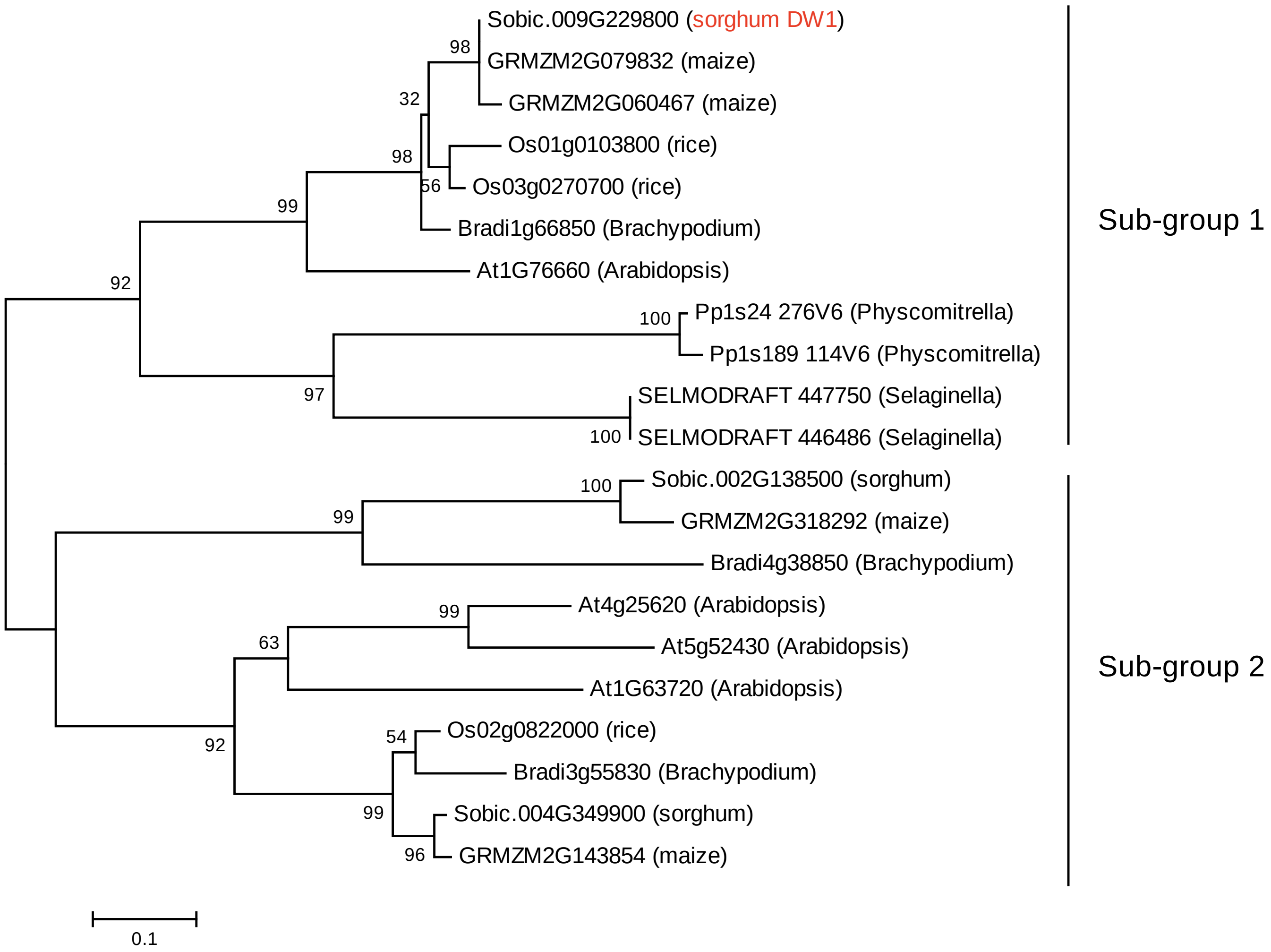

Supplementary Figure S5 | Phylogenetic analysis of DW1. DW1 homologs of *Oryza sativa* (rice), *Arabidopsis thaliana* (Arabidopsis), *Zea mays* (maize), *Brachypodium distachyon* (Brachypodium), *Physcomitrella patens* (Physcomitrella), and *Selaginella moellendorffii* (Selaginella) were used for this analysis. Numbers at the branches are bootstrap values. The horizontal branch lengths are proportional to the estimated number of amino acid substitutions per residue. The DW1 homologs in seed plants are divided into two sub-groups, and the homologs in ferns and mosses are in subgroup I.

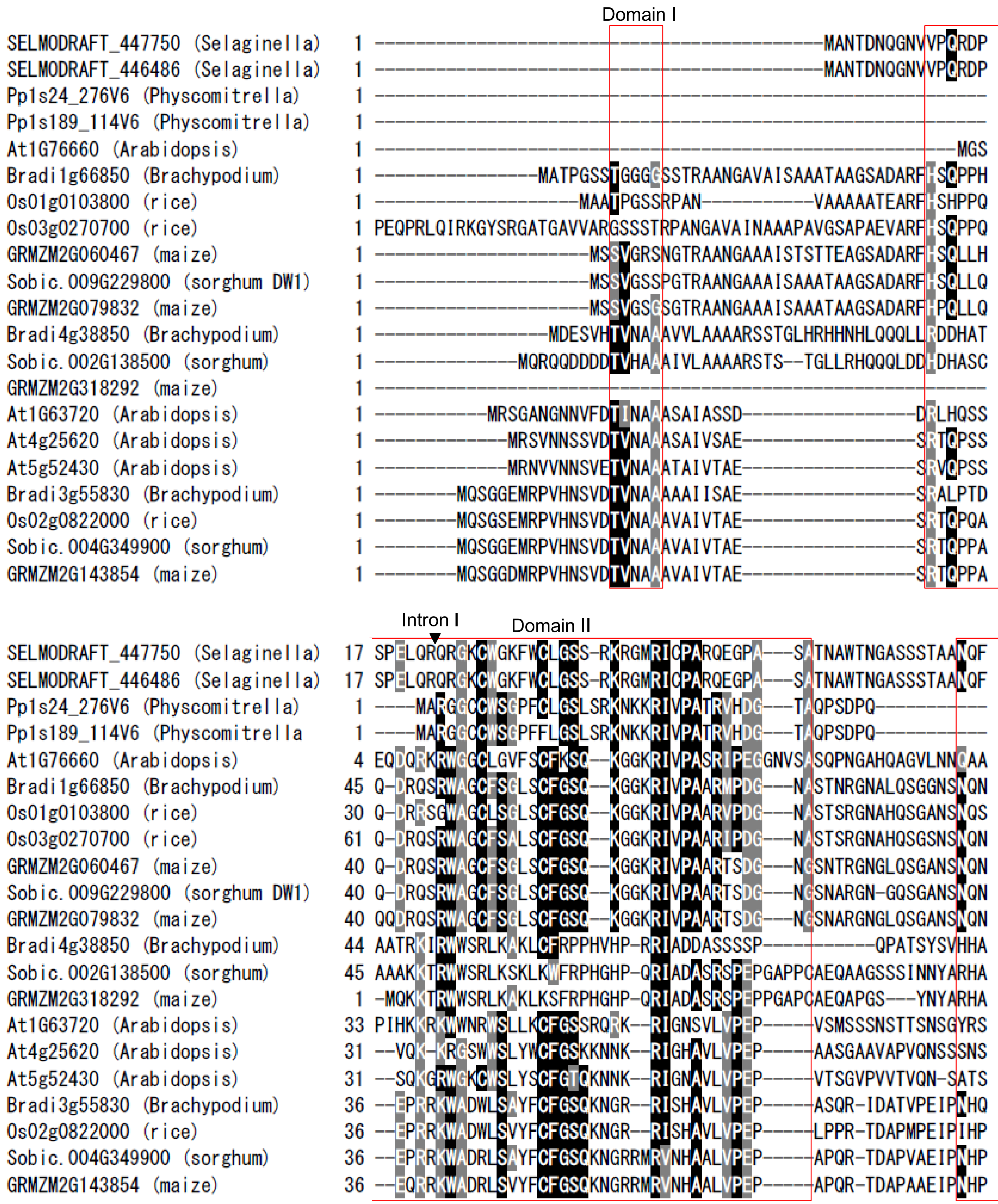
Domain I
SELMODRAFT_447750 (Selaginella) 1 --MANTDNQGNVVPQRDP
SELMODRAFT_446486 (Selaginella) 1 --MANTDNQGNVVPQRDP
Pp1s24_276V6 (Physcomitrella) 1 --
Pp1s189_114V6 (Physcomitrella) 1 --
At1G76660 (Arabidopsis) 1 ---MGS
Bradi1g66850 (Brachypodium) 1 ----------------MATPGSSTGGGGSSTRAANGAVAISAAATAAGSADARFHSQPPH
Os01g0103800 (rice) 1 --------------------MAATPGSSRPAN-----------VAAAAATEARFHSHPPQ
Os03g0270700 (rice) 1 PEQPRLQIRKGYSRGATGAVVARGSSSTRPANGAVAINAAAPAVGSAPAEVARFHSQPPQ
GRMZM2G060467 (maize) 1 ---------------------MSSVGRSNGTRAANGAAAISTSTTEAGSADARFHSQLLH
Sobic.009G229800 (sorghum DW1) 1 ---------------------MSSVGSSPGTRAANGAAAISAAATAAGSADARFHSQLLQ
GRMZM2G079832 (maize) 1 ---------------------MSSVGSGSGTRAANGAAAISAAATAAGSADARFHPQLLQ
Bradi4g38850 (Brachypodium) 1 -----------------MDESVHTVNAAAVVLAAAARSSTGLHRHHNHLQQQLLRDDHAT
Sobic.002G138500 (sorghum) 1 --------------MQRQQDDDDTVHAAAIVLAAAARSTS--TGLLRHQQQLDDHDHASC
GRMZM2G318292 (maize) 1 --
At1G63720 (Arabidopsis) 1 -----------MRSGANGNNVFDTINAAASAIASSD-----------------DRLHQSS
At4g25620 (Arabidopsis) 1 -------------MRSVNNSSVDTVNAAASAIVSAE-----------------SRTQPSS
At5g52430 (Arabidopsis) 1 -------------MRNVVNNSVETVNAAATAIVTAE-----------------SRVQPSS
Bradi3g55830 (Brachypodium) 1 --------MQSGGEMRPVHNSVDTVNAAAAAIISAE-----------------SRALPTD
Os02g0822000 (rice) 1 --------MQSGSEMRPVHNSVDTVNAAAVAIVTAE-----------------SRTQPQA
Sobic.004G349900 (sorghum) 1 --------MQSGGEMRPVHNSVDTVNAAAVAIVTAE-----------------SRTQPPA
GRMZM2G143854 (maize) 1 --------MQSGGDMRPVHNSVDTVNAAAVAIVTAE-----------------SRTQPPA
Intron I
Domain II
SELMODRAFT_447750 (Selaginella) 17 SPELQRQRGKCWGKFWCLGSS-RKRGMRICPARQEGPA---SATNAWTNGASSSTAANQF
SELMODRAFT_446486 (Selaginella) 17 SPELQRQRGKCWGKFWCLGSS-RKRGMRICPARQEGPA---SATNAWTNGASSSTAANQF
Pp1s24_276V6 (Physcomitrella) 1 ----MARGGCCWSGPFCLGSLSRKNKKRIVPATRVHDG---TAQPSDPQ-----------
Pp1s189_114V6 (Physcomitrella) 1 ----MARGGCCWSGPFFLGSLSRKNKKRIVPATRVHDG---TAQPSDPQ-----------
At1G76660 (Arabidopsis) 4 EQDQRKRWGGCLGVFSCFKSQ--KGGKRIVPASRIPEGGNVSASQPNGAHQAGVLNNQAA
Bradi1g66850 (Brachypodium) 45 Q-DRQSRWAGCFSGLSCFGSQ--KGGKRIVPAARMPDG---NASTNRGNALQSGGNSNQN
Os01g0103800 (rice) 30 Q-DRRSGWAGCLSGLSCFGSQ--KGGKRIVPAARVPDG---NASTSRGNAHQSGANSNQS
Os03g0270700 (rice) 61 Q-DRQSRWAGCFSALSCFGSQ--KGGKRIVPAARIPDG---NASTSRGNAHQSGSNSNQN
GRMZM2G060467 (maize) 40 Q-DRQSRWAGCFSGLSCFGSQ--KGGKRIVPAARTSDG---NGSNTRGNGLQSGANSNQN
Sobic.009G229800 (sorghum DW1) 40 Q-DRQSRWAGCFSGLSCFGSQ--KGGKRIVPAARTSDG---NGSNARGN-GQSGANSNQN
GRMZM2G079832 (maize) 40 QQDRQSRWAGCFSGLSCFGSQ--KGGKRIVPAARTSDG---NGSNARGNGLQSGANSNQN
Bradi4g38850 (Brachypodium) 44 AATRKIRWWSRLKAKLCFRPPHVHP-RRIADDASSSP------------QPATSYSVHHA
Sobic.002G138500 (sorghum) 45 AAAKKTRWWSRLKSKLKWFRPHGHP-QRIADASRSPEPGAPPCAEQAAGSSSINNYARHA
GRMZM2G318292 (maize) 1 -MQKKTRWWSRLKAKLKSFRPHGHP-QRIADASRSPEPPGAPCAEQAPGS---YNYARHA
At1G63720 (Arabidopsis) 33 PIHKKRKWWNRWSLLKCFGSSRQRK--RIGNSVLVPEP-----VSMSSSNSTTSNSGYRS
At4g25620 (Arabidopsis) 31 --VQK-KRGSWWSLYWCFGSKKNNK--RIGHAVLVPEP-----AASGAAVAPVQNSSSNS
At5g52430 (Arabidopsis) 31 --SQKGRWGKCWSLYSCFGTQKNNK--RIGNAVLVPEP-----VTSGVPVVTVQN-SATS
Bradi3g55830 (Brachypodium) 36 --EPRRKWADWLSAYFCFGSQKNGR--RISHAVLVPEP-----ASQR-IDATVPEIPNHQ
Os02g0822000 (rice) 36 --EPRRKWADWLSVYFCFGSQKNGR--RISHAVLVPEP-----LPPR-TDAPMPEIPIHP
Sobic.004G349900 (sorghum) 36 --EPRRKWADRLSAYFCFGSQKNGRRMRVNHAALVPEP-----APQR-TDAPVAEIPNHP
GRMZM2G143854 (maize) 36 --EQRRKWADRLSVYFCFGSQKNGRRMRVNHAALVPEP-----APQR-TDAPAAEIPNHP

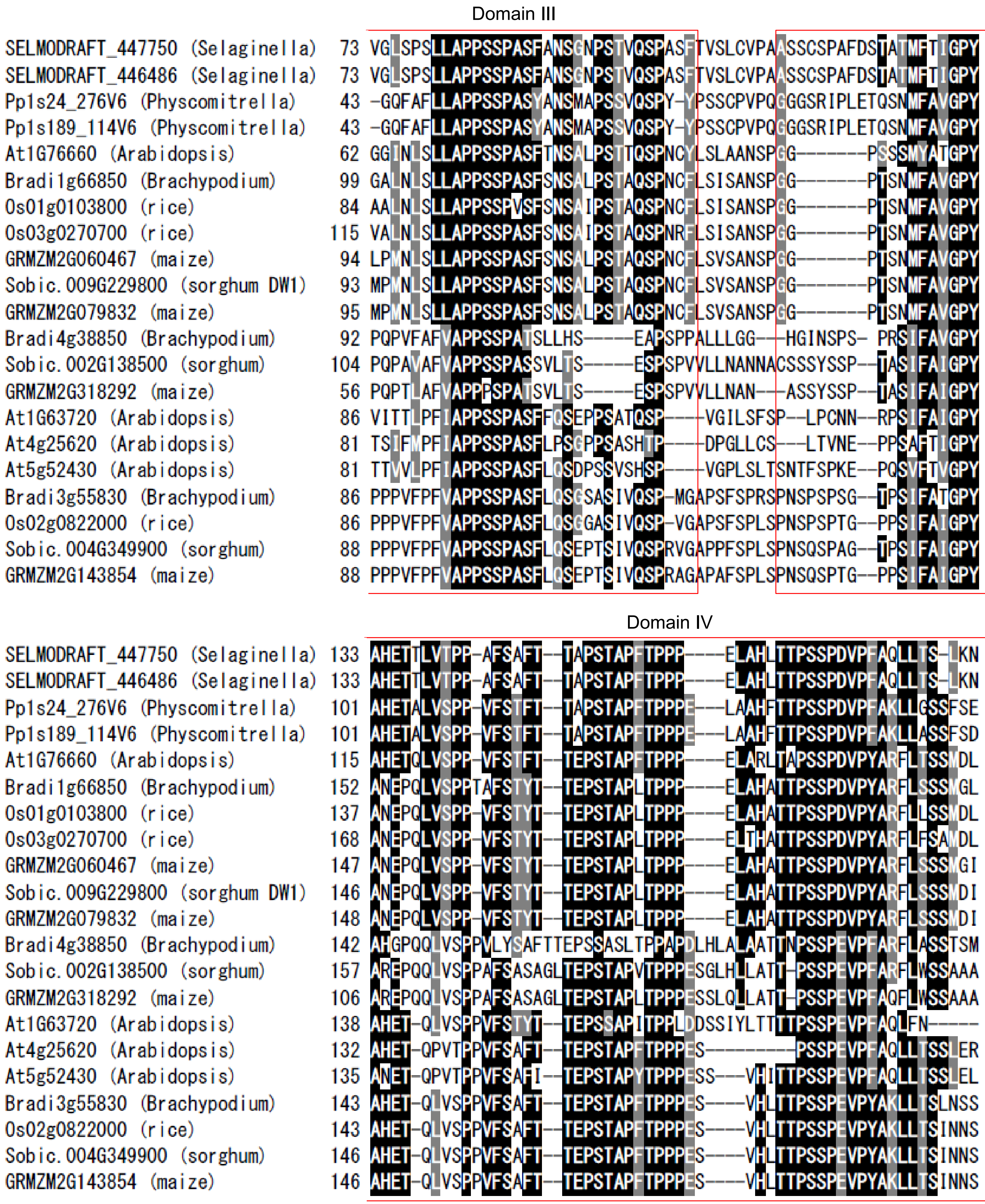
Domain III
SELMODRAFT_447750 (Selaginella) 73 VGLSPSLLAPPSSPASFANSGNPSTVQSPASFTVSLCVPAASSCSPAFDSTATMFTIGPY
SELMODRAFT_446486 (Selaginella) 73 VGLSPSLLAPPSSPASFANSGNPSTVQSPASFTVSLCVPAASSCSPAFDSTATMFTIGPY
Pp1s24_276V6 (Physcomitrella) 43 -GQFAFLLAPPSSPASYANSMAPSSVQSPY-YPSSCPVPQGGGSRIPLETQSNMFAVGPY
Pp1s189_114V6 (Physcomitrella) 43 -GQFAFLLAPPSSPASYANSMAPSSVQSPY-YPSSCPVPQGGGSRIPLETQSNMFAVGPY
At1G76660 (Arabidopsis) 62 GGINLSLLAPPSSPASFTNSALPSTTQSPNCYLSLAANSPGG-------PSSSMYATGPY
Bradi1g66850 (Brachypodium) 99 GALNLSLLAPPSSPASFSNSALPSTAQSPNCFLSISANSPGG-------PTSNMFAVGPY
Os01g0103800 (rice) 84 AALNLSLLAPPSSPVSFSNSAIPSTAQSPNCFLSISANSPGG-------PTSNMFAVGPY
Os03g0270700 (rice) 115 VALNLSLLAPPSSPASFSNSAIPSTAQSPNRFLSISANSPGG-------PTSNMFAVGPY
GRMZM2G060467 (maize) 94 LPMNLSLLAPPSSPASFSNSALPSTAQSPNCFLSVSANSPGG-------PTSNMFAVGPY
Sobic.009G229800 (sorghum DW1) 93 MPMNLSLLAPPSSPASFSNSALPSTAQSPNCFLSVSANSPGG-------PTSNMFAVGPY
GRMZM2G079832 (maize) 95 MPMNLSLLAPPSSPASFSNSALPSTAQSPNCFLSVSANSPGG-------PTSNMFAVGPY
Bradi4g38850 (Brachypodium) 92 PQPVFAFVAPPSSPATSLLHS-----EAPSPPALLLGG---HGINSPS- PRSIFAVGPY
Sobic.002G138500 (sorghum) 104 PQPAVAFVAPPSSPASSVLTS-----ESPSPVVLLNANNACSSSYSSP--TASIFAIGPY
GRMZM2G318292 (maize) 56 PQPTLAFVAPPPSPATSVLTS-----ESPSPVVLLNAN---ASSYSSP--TASIFAIGPY
At1G63720 (Arabidopsis) 86 VITTLPFIAPPSSPASFFQSEPPSATQSP----VGILSFSP--LPCNN--RPSIFAIGPY
At4g25620 (Arabidopsis) 81 TSIFMPFIAPPSSPASFLPSGPPSASHTP----DPGLLCS---LTVNE--PPSAFTIGPY
At5g52430 (Arabidopsis) 81 TTVVLPFIAPPSSPASFLQSDPSSVSHSP----VGPLSLTSNTFSPKE--PQSVFTVGPY
Bradi3g55830 (Brachypodium) 86 PPPVFPFVAPPSSPASFLQSGSASIVQSP-MGAPSFSPRSPNSPSPSG--TPSIFATGPY
Os02g0822000 (rice) 86 PPPVFPFVAPPSSPASFLQSGGASIVQSP-VGAPSFSPLSPNSPSPTG--PPSIFAIGPY
Sobic.004G349900 (sorghum) 88 PPPVFPFVAPPSSPASFLQSEPTSIVQSPRVGAPPFSPLSPNSQSPAG--TPSIFAIGPY
GRMZM2G143854 (maize) 88 PPPVFPFVAPPSSPASFLQSEPTSIVQSPRAGAPAFSPLSPNSQSPTG--PPSIFAIGPY
Domain IV
SELMODRAFT_447750 (Selaginella) 133 AHETTLVTPP-AFSAFT--TAPSTAPFTPPP----ELAHLTTPSSPDVPFAQLLTS-LKN
SELMODRAFT_446486 (Selaginella) 133 AHETTLVTPP-AFSAFT--TAPSTAPFTPPP----ELAHLTTPSSPDVPFAQLLTS-LKN
Pp1s24_276V6 (Physcomitrella) 101 AHETALVSPP-VFSTFT--TAPSTAPFTPPPE---LAAHFTTPSSPDVPFAKLLGSSFSE
Pp1s189_114V6 (Physcomitrella) 101 AHETALVSPP-VFSTFT--TAPSTAPFTPPPE---LAAHFTTPSSPDVPFAKLLASSFSD
At1G76660 (Arabidopsis) 115 AHETQLVSPP-VFSTFT--TEPSTAPFTPPP----ELARLTAPSSPDVPYARFLTSSMDL
Bradi1g66850 (Brachypodium) 152 ANEPQLVSPPTAFSTYT--TEPSTAPLTPPP----ELAHATTPSSPDVPYARFLSSSMGL
Os01g0103800 (rice) 137 ANEPQLVSPP-VFSTYT--TEPSTAPLTPPP----ELAHATTPSSPDVPYARFLLSSMDL
Os03g0270700 (rice) 168 ANEPQLVSPP-VFSTYT--TEPSTAPLTPPP----ELTHATTPSSPDVPYARFLFSAMDL
GRMZM2G060467 (maize) 147 ANEPQLVSPP-VFSTYT--TEPSTAPLTPPP----ELAHATTPSSPDVPYARFLSSSMGI
Sobic.009G229800 (sorghum DW1) 146 ANEPQLVSPP-VFSTYT--TEPSTAPLTPPP----ELAHATTPSSPDVPYARFLSSSMDI
GRMZM2G079832 (maize) 148 ANEPQLVSPP-VFSTYT--TEPSTAPLTPPP----ELAHATTPSSPDVPYARFLSSSMDI
Bradi4g38850 (Brachypodium) 142 AHGPQQLVSPPVLYSAFTTEPSSASLTPPAPDLHLALAATTNPSSPEVPFARFLASSTSM
Sobic.002G138500 (sorghum) 157 AREPQQLVSPPAFSASAGLTEPSTAPVTPPPESGLHLLATT-PSSPEVPFARFLWSSAAA
GRMZM2G318292 (maize) 106 AREPQQLVSPPAFSASAGLTEPSTAPLTPPPESSLQLLATT-PSSPEVPFAQFLWSSAAA
At1G63720 (Arabidopsis) 138 AHET-QLVSPPVFSTYT--TEPSSAPITPPLDDSSIYLTTTTPSSPEVPFAQLFN-----
At4g25620 (Arabidopsis) 132 AHET-QPVTPPVFSAFT--TEPSTAPFTPPPES---------PSSPEVPFAQLLTSSLER
At5g52430 (Arabidopsis) 135 ANET-QPVTPPVFSAFI--TEPSTAPYTPPPESS---VHITTPSSPEVPFAQLLTSSLEL
Bradi3g55830 (Brachypodium) 143 AHET-QLVSPPVFSAFT--TEPSTAPFTPPPES----VHLTTPSSPEVPYAKLLTSLNSS
Os02g0822000 (rice) 143 AHET-QLVSPPVFSAFT--TEPSTAPFTPPPES----VHLTTPSSPEVPYAKLLTSINNS
Sobic.004G349900 (sorghum) 146 AHET-QLVSPPVFSAFT--TEPSTAPFTPPPES----VHLTTPSSPEVPYAKLLTSINNS
GRMZM2G143854 (maize) 146 AHET-QLVSPPVFSAFT--TEPSTAPFTPPPES----VHLTTPSSPEVPYAKLLTSINNS

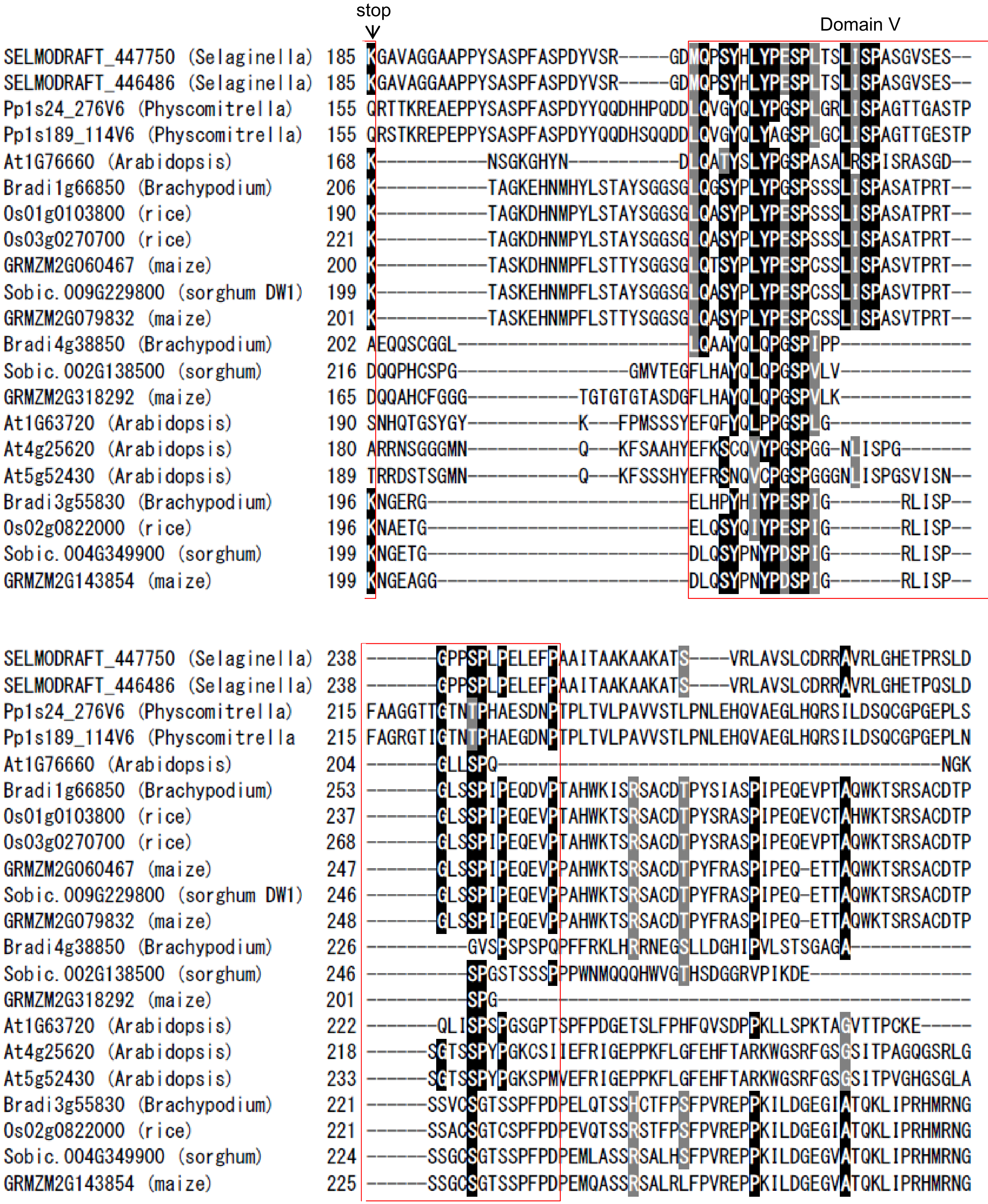
stop
Domain V
SELMODRAFT_447750 (Selaginella) 185 KGAVAGGAAPPYSASPFASPDYVSR-----GDMCPSYHLYPESPLTSLISPASGVSES--
SELMODRAFT_446486 (Selaginella) 185 KGAVAGGAAPPYSASPFASPDYVSR-----GDMCPSYHLYPESPLTSLISPASGVSES--
Pp1s24_276V6 (Physcomitrella) 155 QRTTKREAEPPYSASPFASPDYYQQDHHPQDDLQVGYQLYPGSPLGRLISPAGTTGASTP
Pp1s189_114V6 (Physcomitrella) 155 QRSTKREPEPPYSASPFASPDYYQQDHSQQDDLQVGYQLYAGSPLGCLISPAGTTGESTP
At1G76660 (Arabidopsis) 168 K-----------NSGKGHYN-----------DLCATYSLYPGSPASALRSPISRASGD--
Bradi1g66850 (Brachypodium) 206 K-----------TAGKEHNMHYLSTAYSGGSGLQGSYPLYPGSPSSSLISPASATPRT--
Os01g0103800 (rice) 190 K-----------TAGKDHNMPYLSTAYSGGSGLQASYPLYPESPSSSLISPASATPRT--
Os03g0270700 (rice) 221 K-----------TAGKDHNMPYLSTAYSGGSGLQASYPLYPESPSSSLISPASATPRT--
GRMZM2G060467 (maize) 200 K-----------TASKDHNMPFLSTTYSGGSGLQTSYPLYPESPCSSLISPASVTPRT--
Sobic.009G229800 (sorghum DW1) 199 K-----------TASKEHNMPFLSTAYSGGSGLQASYPLYPESPCSSLISPASVTPRT--
GRMZM2G079832 (maize) 201 K-----------TASKEHNMPFLSTTYSGGSGLQASYPLYPESPCSSLISPASVTPRT--
Bradi4g38850 (Brachypodium) 202 AEQQSCGGL------------------------LQAAYQLQPGSPIPP-----------
Sobic.002G138500 (sorghum) 216 DQQPHCSPG-----------------GMVTEGFLHAYQLQPGSPVLV-----------
GRMZM2G318292 (maize) 165 DQQAHCFGGG-----------TGTGTGTASDGFLHAYQLQPGSPVLK-----------
At1G63720 (Arabidopsis) 190 SNHQTGSYGY-----------K---FPMSSSYEFQFYQLPPGSPLG------------
At4g25620 (Arabidopsis) 180 ARRNSGGGMN-----------Q---KFSAAHYEFKSCQVYPGSPGG-NLISPG-------
At5g52430 (Arabidopsis) 189 TRRDSTSGMN-----------Q---KFSSSHYEFRSNQVCPGSPGGGNLISPGSVISN--
Bradi3g55830 (Brachypodium) 196 KNGERG-------------------------ELHPYHIYPESPIG-------RLISP--
Os02g0822000 (rice) 196 KNAETG-------------------------ELQSYQIYPESPIG-------RLISP--
Sobic.004G349900 (sorghum) 199 KNGETG-------------------------DLQSYPNYPDSPIG-------RLISP--
GRMZM2G143854 (maize) 199 KNGEAGG------------------------DLQSYPNYPDSPIG-------RLISP--
SELMODRAFT_447750 (Selaginella) 238 -------GPPSPLPELEFPAAITAAKAAKATS----VRLAVSLCDRRAVRLGHETPRSLD
SELMODRAFT_446486 (Selaginella) 238 -------GPPSPLPELEFPAAITAAKAAKATS----VRLAVSLCDRRAVRLGHETPQSLD
Pp1s24_276V6 (Physcomitrella) 215 FAAGGTTGTNTPHAESDNPTPLTVLPAVVSTLPNLEHQVAEGLHQRSILDSQCGPGEPLS
Pp1s189_114V6 (Physcomitrella 215 FAGRGTIGTNTPHAEGDNPTPLTVLPAVVSTLPNLEHQVAEGLHQRSILDSQCGPGEPLN
At1G76660 (Arabidopsis) 204 -------GLLSPQ--NGK
Bradi1g66850 (Brachypodium) 253 -------GLSSPIPEQDVPTAHWKISRSACDTPYSIASPIPEQEVPTAQWKTSRSACDTP
Os01g0103800 (rice) 237 -------GLSSPIPEQEVPTAHWKTSRSACDTPYSRASPIPEQEVCTAHWKTSRSACDTP
Os03g0270700 (rice) 268 -------GLSSPIPEQEVPTAHWKTSRSACDTPYSRASPIPEQEVPTAQWKTSRSACDTP
GRMZM2G060467 (maize) 247 -------GLSSPIPEQEVPPAHWKTSRSACDTPYFRASPIPEQ-ETTAQWKTSRSACDTP
Sobic.009G229800 (sorghum DW1) 246 -------GLSSPIPEQEVPPAHWKTSRSACDTPYFRASPIPEQ-ETTAQWKTSRSACDTP
GRMZM2G079832 (maize) 248 -------GLSSPIPEQEVPPAHWKTSRSACDTPYFRASPIPEQ-ETTAQWKTSRSACDTP
Bradi4g38850 (Brachypodium) 226 ----------GVSPSPSPQPFFRKLHRRNEGSLLDGHIPVLSTSGAGA----------
Sobic.002G138500 (sorghum) 246 -----------SPGSTSSSPPPWNMQQQHWVGTHSDGGRVPIKDE-------------
GRMZM2G318292 (maize) 201 -----------SPG---
At1G63720 (Arabidopsis) 222 -------QLISPSPGSGPTSPFPDGETSLFPHFQVSDPPKLLSPKTAGVTTPCKE-----
At4g25620 (Arabidopsis) 218 ------SGTSSPYPGKCSIIEFRIGEPPKFLGFEHFTARKWGSRFGSGSITPAGQGSRLG
At5g52430 (Arabidopsis) 233 ------SGTSSPYPGKSPMVEFRIGEPPKFLGFEHFTARKWGSRFGSGSITPVGHGSGLA
Bradi3g55830 (Brachypodium) 221 ------SSVCSGTSSPFPDPELQTSSHCTFPSFPVREPPKILDGEGIATQKLIPRHMRNG
Os02g0822000 (rice) 221 ------SSACSGTCSPFPDPEVQTSSRSTFPSFPVREPPKILDGEGIATQKLIPRHMRNG
Sobic.004G349900 (sorghum) 224 ------SSGCSGTSSPFPDPEMLASSRSALHSFPVREPPKILDGEGVATQKLIPRHMRNG
GRMZM2G143854 (maize) 225 ------SSGCSGTSSPFPDPEMQASSRSALRLFPVREPPKILDGEGVATQKLIPRHMRNG

```
SELMODRAFT_447750 (Selaginella)     287 SNNHSRELEPIKILSPPRGQYEERWCSRTLDSCSTSSNAVAATKSMEELPRRSHSCG---
SELMODRAFT_446486 (Selaginella)     287 SNNHSRELEPIKILSPPRGQYEERWCSRTLDSCSTSSNAVAATKSMEELPRRSHSCG---
Pp1s24_276V6 (Physcomitrella)       275 DSGRERHGSFDSHSRFMVAMIHERDGSDSNSNSYGHERYSGSLTGLNDVLEGRNRYT---
Pp1s189_114V6 (Physcomitrella)      275 DSGRERHGSFDSHSRFMGAMIHERDPSDSASHSYGHEQHSGSLSGLHEVLEGRNRHR---
At1G76660 (Arabidopsis)            213 CSRSDSGNTFGYDTNGVSTPLQESNFFCPETFAKFYLDHDPSVPQNG--GRLSVSKDSDV
Bradi1g66850 (Brachypodium)        306 YSRTSPSNIFGLDSAAPRNCLLDSNFFRPAASAQFYLDQAQQTFPYN-GGRLSVSRD---
Os01g0103800 (rice)                290 YSRNSPSNIFGLDSAASRNYMLDNNFFRPAASAQFYLDQAQQSFPYNNGGRISVSKD---
Os03g0270700 (rice)                321 YSRNSPSNIFGLDSAASRNYMLDNNFFRPAASAQFYLDQAQQSFPYNNGGRISVSRD---
GRMZM2G060467 (maize)              299 YARTSPTNIFGLDSSTPRNYMLDNNFFRPAASAQFYLDQAQQTFSHN-GGRVSVSRE---
Sobic.009G229800 (sorghum DW1)     298 YARNSPTNIFGLDSSTPRNYMLDSNFFRPAASAQFYLDQAQQTFPHN-GGRVSVSRE---
GRMZM2G079832 (maize)              300 YARTSPTNIFGLDSNTPRNYMLDSNFFRPAASAQFYLDQAQQAFPHN-GGRVSVSRE---
Bradi4g38850 (Brachypodium)        264 ------------------------------------------------------------
Sobic.002G138500 (sorghum)         280 ------------------------------------------------------------
GRMZM2G318292 (maize)                  ------------------------------------------------------------
At1G63720 (Arabidopsis)            270 ------------------------------------------------------------
At4g25620 (Arabidopsis)            272 SGALTPDGSKLTSGVVTPNGAETVIRMSYGNLTPLEGSLLDSQISEVASLANSDHGSSR-
At5g52430 (Arabidopsis)            287 SGALTPNGPEIVSGNLTPN---------------NTTWPLQNQISEVASLANSDHG----
Bradi3g55830 (Brachypodium)        275 GSLLDGHITAAVPVVDFSARLQ--------------------------------------
Os02g0822000 (rice)                275 GSLLDGHISAAVPVVDFSARLQ--------------------------------------
Sobic.004G349900 (sorghum)         278 GSLLDGHITAAVPVVDFSARLQ--------------------------------------
GRMZM2G143854 (maize)              279 GSLLDGQISAAVPVVDFSARLQ--------------------------------------

                                                                 Domain VI
SELMODRAFT_447750 (Selaginella)     344 --------------------GIDEVELKRSLSQVGALPRGGDDDDRADADVDTMVVEDSQ
SELMODRAFT_446486 (Selaginella)     344 --------------------GIDEVELKRSLSQVGALPRGGDDDDRADADVDTMVVEDSQ
Pp1s24_276V6 (Physcomitrella)       332 --------------------KLKQDKGPRSSSLRSHEKE-DDIQEEDLLELPMLLGSESS
Pp1s189_114V6 (Physcomitrella)      332 --------------------KLNQDKSSRFTGLKTQEQEKDDVRNEDLLELPMLLGSESS
At1G76660 (Arabidopsis)            271 YPTNGYGNGNQNRQNRSPKQDMEELEAYRASFGFSADEIITTSQYVEITDVMDGSFNTSA
Bradi1g66850 (Brachypodium)        362 ------------------KQDADEVEAYRASFGFSADEIVTTQHYAEIPDTLDDGFSISP
Os01g0103800 (rice)                347 ------------------KQDVEEVEAYRASFGFSADEIVTTQTYVEIPDALDEGFSISP
Os03g0270700 (rice)                378 ------------------KQDAEEVEAYRASFGFSADEIVTTQAYVEIPDALDEGFSISP
GRMZM2G060467 (maize)              355 ------------------KQGADEIEAYRASFGFSADEIVQSQSYVGIPDAVDESFSISP
Sobic.009G229800 (sorghum DW1)     354 ------------------KQDADEIEAYRASFGFSADEIVQSQSYVGIPDAVDESFSISP
GRMZM2G079832 (maize)              356 ------------------KQDADEIEAYRASFGFSADEIVQSQSYVGIPDAVDESFSISP
Bradi4g38850 (Brachypodium)        264 ------------------------------------------------------------
Sobic.002G138500 (sorghum)         280 ------------------------------------------------------------
GRMZM2G318292 (maize)                  ------------------------------------------------------------
At1G63720 (Arabidopsis)            270 --------------------QKIVRPHKPVSFDLDADHVIRCVDQKLRTTFP--------
At4g25620 (Arabidopsis)            331 ------------------HNDEALVVPHRVSFELTGEDVARCLASKLNRSGS--------
At5g52430 (Arabidopsis)            328 --------------------SEVMVADHRVSFELTGEDVARCLASKLNRSHDRMNNNDRI
Bradi3g55830 (Brachypodium)        297 --------------------SNDHAMDHRVSFELTVEDVARCLEKKTAISGDSAQSSFHL
Os02g0822000 (rice)                297 --------------------NNDHAMDHRVSFELTVEDVARCLEKKTNINGESAAASFRL
Sobic.004G349900 (sorghum)         300 --------------------PNEHAMDHRVSFELTVEDVARCLEKKTAISGDSSTASFHL
GRMZM2G143854 (maize)              301 --------------------PNEHAMDHRVSFELTVEDVARCLEKKTAISGDSGTASFHL
```

```
SELMODRAFT_447750 (Selaginella)   384 QPTSALLRG-------------------------------GDDDISDSLRDEEGRTEKNN
SELMODRAFT_446486 (Selaginella)   384 QPTSALLRG-------------------------------GDDDISDSLRDEEGRTEKNN
Pp1s24_276V6 (Physcomitrella)     371 HGGSRASRSPSSRSKVQSKAGSRVGSRVESKAASRVGSKAGSDALKDWDHNEDNLLEYAL
Pp1s189_114V6 (Physcomitrella)    372 QGGSRASRSPNNRSKVQSKAG------------SRVESKAGSDSLKEWDRNENNLLEYAL
At1G76660 (Arabidopsis)           331 YS---------------------------------------PSDGQKLLRREANLLSQTS
Bradi1g66850 (Brachypodium)       404 FGNSAPAAEVSPFNDLPNEAQ------------KVDKSLLNAKAITSPKK-SADQLSSGT
Os01g0103800 (rice)               389 FGNNAPATEVDKS-------------------------LFNVKVITGPKKSTEQKLSNGS
Os03g0270700 (rice)               420 FGNNAPATEVDKP-------------------------LFNVKVTTSPKK-SADQLSNDS
GRMZM2G060467 (maize)             397 FGNNTPATEICPFSDLP---N------------EVDKSCAYAKDDTSPKK-SANQLSTDS
Sobic.009G229800 (sorghum DW1)    396 FGNNAPATEICPFSDLPNEVQ------------KVDKSCAYAKDGTSPKK-SANQLSIDS
GRMZM2G079832 (maize)             398 FGNNAPATEICPFSDLPSEVQ------------KADKSCTYVKDGTSPKK-SANQLSIDS
Bradi4g38850 (Brachypodium)       264 ------------------------------------------------------------
Sobic.002G138500 (sorghum)        280 ------------------------------------------------------------
GRMZM2G318292 (maize)                 ------------------------------------------------------------
At1G63720 (Arabidopsis)           302 ------------------------------------------------------------
At4g25620 (Arabidopsis)           365 -HEKASG------------------------------------------------EHLRP
At5g52430 (Arabidopsis)           368 ETEESSS------------------------------------------------TDIRR
Bradi3g55830 (Brachypodium)       337 LPTSNGDHSR----------------------------------------------ESNE
Os02g0822000 (rice)               337 VPTGNG---DHIHP-----------------------------------------RESND
Sobic.004G349900 (sorghum)        340 APTSNG---DHHR-------------------------------------------ESNE
GRMZM2G143854 (maize)             341 APTGSG---DHHR-------------------------------------------ESNE

                                                 Intron II
                                                     ▼
SELMODRAFT_447750 (Selaginella)   413 DLAA--TATAETTTHSRPIEEDGVGVCSSSSSSSCLDKSGDVEGEPKRYEEQALESPRSD
SELMODRAFT_446486 (Selaginella)   413 DLG----ATAETTTHSRPIEEDGVGVCSSSSSSSCLDKSGDVEGEAKRFEEQALESPRSD
Pp1s24_276V6 (Physcomitrella)     431 SLLEGTDGKRPGTIGWAPVDTSLPRESEGPIQSSSTTRNETEENEKDTVYKSIKNGKGSD
Pp1s189_114V6 (Physcomitrella)    420 SLLEGSDGKRPGTIGWAPVDTSLPREPEASTQSSSDLSNGTEENETDRVYKGDQNGKSSS
At1G76660 (Arabidopsis)           352 PKSEADLDSQVVDFQSPKSSNSYKDHKQRNRIHADEEALLSRVGSVKGSRSYHISSSDAE
Bradi1g66850 (Brachypodium)       451 PQKVLHLDIFKGTKGGHLSDDDGIAKDCHPFRKSRDEISLKPIEVRKKSPPGQA-CSDAE
Os01g0103800 (rice)               424 PQNVVHLDIFKGTKGGDVCEDEGMVKDCHPFRKGRDEISLKPIEVRKKVGGGQS-CSDAE
Os03g0270700 (rice)               454 PHNVVHLDIFKGTKGGDLSEDEGVVKDCHPFRKAMDEISLKPIEVRKKVQPGQSSSSDAE
GRMZM2G060467 (maize)             441 PNKVLRLDVFKGTKGGHQSEDECIRKDGHLFRKTADEISLKPIEVRKKSLPGHS-CSDAE
Sobic.009G229800 (sorghum DW1)    443 PNKVLRLDVFKGTKGGHQSEDEGIVKDGHPFRRTTDEISLKPIEVRKKSLPGHS-CSDAE
GRMZM2G079832 (maize)             445 PNKVLRLDVFKVTKGGHLSEDEGIARDGHPFRRTTDEISLKPIEVRKKSPPGHS-CSDAE
Bradi4g38850 (Brachypodium)       264 ----GSDQEEEGRGGSGGDDDDEVPKSGEFVFGNADDGAAEDVD----------------
Sobic.002G138500 (sorghum)        280 -----SKDDEVATGGGEFVFGSNADAAAGERFAGGEVGARGDAT----------------
GRMZM2G318292 (maize)                 ------------------------------------------------------------
At1G63720 (Arabidopsis)           302 ---EASSDQESMNHSSLGSNKEFNFGTDEKHLTVDEHRSASPK--NSNDWS---------
At4g25620 (Arabidopsis)           376 NCCKTSGETESEQS--QKLRSFSTGSNKEFKFDSTNEEMIEKI--RSEWWANEKVAGKGD
At5g52430 (Arabidopsis)           380 NIEKRSGDRENEQHRIQKLSSSSIGSSKEFKFDNTKDENIEKV--AGNSWS---------
Bradi3g55830 (Brachypodium)       351 TRAGLYVDETYHDLPEKARRSLSLRLAKEFKFNNIDGAHVEETGALGSDWWANDKVAAIT
Os02g0822000 (rice)               353 TRAGLCVDETYHDLPEKARRSLSLRKAKEFKFNNVDAPSVEPS--VGSDWWANEKVAGIT
Sobic.004G349900 (sorghum)        354 ARAGLYVDETYHDLPEKARRSLSLRLAKDFNFNNVDVANVEPS--VGSDWWANEKVAGMT
GRMZM2G143854 (maize)             355 ARAGLYVDESYHDLPEKARRSLSLRLAKEFNFNNVDVGSVEPS--VGSDWWANEKVAGMT
```

```
SELMODRAFT_447750 (Selaginella)  471 CGIVLEVEITPDDLVKTSWSSQAK------------------------------------
SELMODRAFT_446486 (Selaginella)  469 CGIVLEVEITPDDLVKVNITGAKNK---------KKNGIVTKKNGFHHHHHHHNHRNGFV
Pp1s24_276V6 (Physcomitrella)    491 NVPQQVVGAGAKEVSEGTITPVGHHGSAPDCCSRCESLVSQCEQLSVALKEARRKQQEKD
Pp1s189_114V6 (Physcomitrella)   480 EGFQEAISAGAIEEPEETVVSAENH----DCSSRYESLVSQYEQLSVALKEAERKQLEKD
At1G76660 (Arabidopsis)          412 VEYRRGRSLRESRENRHRKA----------------------------------------
Bradi1g66850 (Brachypodium)      510 IEYRRARSLREANSVLSWRSTLARQLQ---------------------------------
Os01g0103800 (rice)              483 IEYRRARSLREANGVVSWRSTLARQLQ---------------------------------
Os03g0270700 (rice)              514 IEYRRARSLREANGVLSWRSTLARQLQ---------------------------------
GRMZM2G060467 (maize)            500 IEYRRTRSLRDANGVLSRRSALARQLH---------------------------------
Sobic.009G229800 (sorghum DW1)   502 IEYRRTRSLRDANGVLSRRSALARQLH---------------------------------
GRMZM2G079832 (maize)            504 IEYRRTRSLRDANGVLSRRSALARQLH---------------------------------
Bradi4g38850 (Brachypodium)      304 ------GNRWPAFPPRG-------------------------------------------
Sobic.002G138500 (sorghum)       319 ------EQWPFLLAHTHS------------------------------------------
GRMZM2G318292 (maize)                ------------------------------------------------------------
At1G63720 (Arabidopsis)          348 --------FFPVMQSGTLS-----------------------------------------
At4g25620 (Arabidopsis)          432 HSPRNSWTFFPVLRSGHT------------------------------------------
At5g52430 (Arabidopsis)          429 --------FFPGLRSGVS------------------------------------------
Bradi3g55830 (Brachypodium)      411 TEPRKSWSFRPMAQPGVS------------------------------------------
Os02g0822000 (rice)              411 SEPRKSWSFFPVAQPGVS------------------------------------------
Sobic.004G349900 (sorghum)       412 SEPKKNWSFHPVAQPGVS------------------------------------------
GRMZM2G143854 (maize)            413 TEPKKNWSFHPVVQPGVS------------------------------------------


SELMODRAFT_447750 (Selaginella)      ----------------------------------------------
SELMODRAFT_446486 (Selaginella)  521 ASSKSSWEDLDAMSVDCMELNLTDNTIKSPLKSSPIMDSC------
Pp1s24_276V6 (Physcomitrella)    551 RLAEEREKQIRHLTQLLQSGGLQKFVGDFPKAANQVGEGNNVKDIS
Pp1s189_114V6 (Physcomitrella)   536 RLAEEREQQKRHLTHLLQSGGLQKLVGDYLRAGNEVGKNKKTKDAP
At1G76660 (Arabidopsis)              ----------------------------------------------
Bradi1g66850 (Brachypodium)          ----------------------------------------------
Os01g0103800 (rice)                  ----------------------------------------------
Os03g0270700 (rice)                  ----------------------------------------------
GRMZM2G060467 (maize)                ----------------------------------------------
Sobic.009G229800 (sorghum DW1)       ----------------------------------------------
GRMZM2G079832 (maize)                ----------------------------------------------
Bradi4g38850 (Brachypodium)          ----------------------------------------------
Sobic.002G138500 (sorghum)           ----------------------------------------------
GRMZM2G318292 (maize)                ----------------------------------------------
At1G63720 (Arabidopsis)              ----------------------------------------------
At4g25620 (Arabidopsis)              ----------------------------------------------
At5g52430 (Arabidopsis)              ----------------------------------------------
Bradi3g55830 (Brachypodium)          ----------------------------------------------
Os02g0822000 (rice)                  ----------------------------------------------
Sobic.004G349900 (sorghum)           ----------------------------------------------
GRMZM2G143854 (maize)                ----------------------------------------------
```

Supplementary Figure S6 | Amino acid alignment of sorghum DW1 and its homologs. The proteins are same as in Supplementary Fig. S3. The positions of Intron 1 and 2 are indicated by arrowheads. The mutation in bmr-6 is indicated by an arrow. The six conserved domains are indicated by red rectangles.

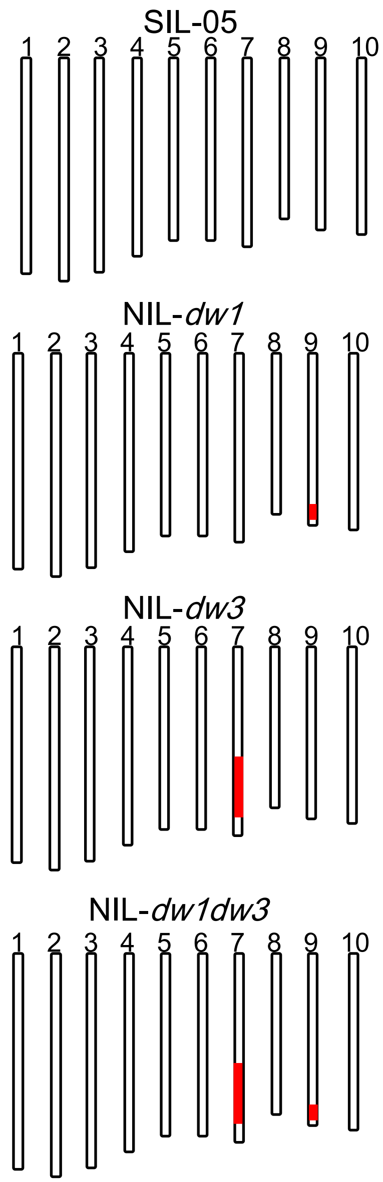

Supplementary Figure S7 | Graphical representations of each NIL. White and red bars indicate chromosomes of SIL-05 and bmr-6, respectively. NIL-*dw1* and NIL-*dw3* contain a ~170 kb and ~2.8 Mb fragments of bmr-6, respectively.

Supplemental Table S1 Primers used in this study

name	sequence	purpose
ssr5-34_R	GTTTGCAGACACCGGGAGTAGATGATGT	QTL
Xcup01_F	ACATGGGCGGGTTGAAGAC	QTL
Xcup01_R	GTTTGCAGGAAGGGAGGATGTAG	QTL
Xtxp79_F	ACTCCACAGCCAGAACATT	QTL
Xtxp79_R	GTTTGAAGCAGTGCAGGATTCAGT	QTL
SB260_F	TCAAGTCCACTCTACTCCTGCCCT	QTL
SB260_R	AAGAAAAGGCTAATCGATTGGGGA	QTL
SB441_F	ACAGAGACTCCGCTGGAAATGAAC	QTL
SB441_R	TGTTTGTTTGGATCGGAGTGAGAA	QTL
SB474_F	TAGGTTGCAATTGATGTTCCAACG	QTL
SB474_R	CACCTACAACTGCGGATACAACCA	QTL
SSR2_491_F	AGACGAAGAAGAGCTTAGCGTGGAG	QTL
SSR2_491_R	GTTTGTAGATGAACCTGGTGTGCTTCCC	QTL
Xtxp88_F	ACGTGAATCAGCGAGTGTTGG	QTL
Xtxp88_R	GTTTGCGTAATGTTCCTGCTC	QTL
Xtxp11_F	ATCGAGAAATTCAACATGCTG	QTL
Xtxp11_R	GTTTGCTAGACCGACGAGATAAG	QTL
Xtxp37_F	ACAACCTAAGAGGCCTATTTAACC	QTL
Xtxp37_R	GTTTACGGCGACTATGTAACTCATAG	QTL
Xtxp335_F	ATATTTCCTCTTGAAAGAATCAGGG	QTL
Xtxp335_R	GTTTATTCATCGAGCAAAAGGCA	QTL
SSR2_630_F	ATAATGGACAAAGCAGAAACGACGA	QTL
SSR2_630_R	GTTTATGAGCAGCGGATAAGTTCGAGAG	QTL
Xtxp279_F	ATTCTGACTTAACCCACCCCTAAA	QTL
Xtxp279_R	GTTTAGCTCATCAATGTCCCAAACC	QTL
SSR2_733_F	ATATTGTGTTGGGGGAAACATAGGG	QTL
SSR2_733_R	GTTTGGCTCAGGAGGCTCTATTGTGTTT	QTL
SSR3_868_F	ACTCATCAGGCTAAAATGATCACCG	QTL
SSR3_868_R	GTTTATCGAGCACCTGAAACATGAAACA	QTL
SSR3_891_F	AGTCCTCTCTCTCCTCTTCTCCGCT	QTL
SSR3_891_R	GTTTGAACACAAGGAATGCGGTGACATA	QTL
SSR5_968_F	ACCTGGAAATCACGAGACGAATCTT	QTL
SSR5_968_R	GTTTAATTAACGCAACACCCTTGAGGAC	QTL
SSR3_987_F	ATACTCCATGTGGCAGATCTCAAGC	QTL
SSR3_987_R	GTTTAATGGAGTGGAGAGTGGAGAGTGG	QTL
SSR5_1004_F	ATGATCCATCATCTTTCCATCCGT	QTL
SSR5_1004_R	GTTTCAAGGGACATATTGTGAGCAGGTG	QTL
Xtxp297_F	AGACCCATATGTGGTTTAGTCGCAAAG	QTL
Xtxp297_R	GTTTGCACAATCTTCGCCTAAATCAACAAT	QTL
Xtxp211_F	ATCAACGGCCAATGATTTCTAAC	QTL
Xtxp211_R	GTTTAGGTTGCGAATAAAAGGTAATGTG	QTL
SB1088_F	GGAAGGGGAGAAATGGTGAAGAAC	QTL
SB1088_R	CACCAACAACATCACGGCCTATTA	QTL
SSR2_1142_F	ATCTACTTGTCCCTCTGCCTCTGCT	QTL
SSR2_1142_R	GTTTGGTTCGCAGCTTTGAATGATACAC	QTL
Xtxp72_F	ATTATGGAAGCAAAATGAC	QTL
Xtxp72_R	GTTTCGAATCCTAATTGAGGTAAGC	QTL
SSR2_1196_F	ACATTGCTAGGTACATGGGCCAAAT	QTL
SSR2_1196_R	GTTTCCCGTTGGTTCACCGTGTATAAAT	QTL
SSR3_1264_F	ACTCAGTTCACGACGATTTGACTGC	QTL
SSR3_1264_R	GTTTACCACGCATCTTTTCGTAGGTCAT	QTL
Xtxp1_F	ATTGGCTTTTGTGGAGCTG	QTL
Xtxp1_R	GTTTACCCAGCAGCACTACACTAC	QTL
Xtxp56_F	ATGTCTTCGTAGTTGCGTGTTG	QTL
Xtxp56_R	GTTTCCGAAGGAGTGCTTTGGAC	QTL
SB1308_F	TTGTATTGCGTGAGACAAAGGGTC	QTL
SB1308_R	CTTCATCAAGTTAGTGTGGCGACG	QTL
SB1311_F	GCCGGAGTAGTGGACTCTGTCTGT	QTL
SB1311_R	AAACCAAAAGCAAGCAAAGGTCG	QTL
SB1343_F	CTAAGGCGTGCCTGTACTGAAACC	QTL
SB1343_R	TGTGACATGTAACGGACTGCTCAA	QTL
SB1362_F	AGCGCATTTGGATGCTGATATGTA	QTL
SB1362_R	TAGAACACACAGCCCTCTTTGCTG	QTL
SB1378_F	AGCTCGGAATTCATTCAAATCAGG	QTL
SB1378_R	ATCTTGCGCGAATCGAAGTCAT	QTL
SB1392_F	TGCTTGCTGCCAATAAAAAGAGTG	QTL
SB1392_R	ATAGCCTCTGTGCGTGTCTTTGTG	QTL
SB1426_F	TGCAATGTGTGTGTTTTCAGGAGA	QTL
SB1426_R	TACAATGGACACTCGTCACACAGC	QTL
Xtxp7_F	ACATCTACTACCCTCTCACC	QTL
Xtxp7_R	GTTTACACATCGAGACCAGTTG	QTL
SB1491_F	GAACTGATCGGTTAACATGTACGGC	QTL
SB1491_R	GGAATGGCTTTGGAGAGAGAGGTT	QTL
SSR4_1518_F	AGATAGGAGGTGCCAGCCAGTATG	QTL
SSR4_1518_R	GTTTCTCTCTCATCCGCTCTCATTCTCC	QTL
SSR2_1526_F	ATGTGTTCCTGTCATTACTTGGCGT	QTL
SSR2_1526_R	GTTTAGCAAAACGGGTCTCCTACGTACC	QTL
SSR3_1562_F	AGAGACGACGCTAATCCATCCAAAC	QTL
SSR3_1562_R	GTTTGCAAGAACACCAGTGCATACATCC	QTL
SSR5_1722_F	AGGCCGGCCGCATTATTTTCTC	QTL
SSR5_1722_R	GTTTAAGAATATTATCTCGTGCGGTGCG	QTL
SSR2_1779_F	ACAAGCACCAAACCATCACAAGAAG	QTL
SSR2_1779_R	GTTTCCTCTGCTCTCGTCTCGTCTCTT	QTL
Xtxp9_F	ACAATAGCACCGCCGCGCG	QTL
Xtxp9_R	GTTTCATTGTGGAGTCCCTGATAC	QTL
SSR2_1814_F	ATTTAATCCAATCCACGGAGAATGC	QTL
SSR2_1814_R	GTTTCGGAAATGATTGCCAGTTTCAGTA	QTL
SSR2_1839_F	ATGAAGAAGCTGTCCGTTTGACAAG	QTL
SSR2_1839_R	GTTTAAAAAGCTGCTCCTTTGAGGCTTT	QTL
SB1862_F	GTGCATAGGGCCAAAAGAGCACTA	QTL
SB1862_R	TGACCAGCATATGGGTCACATCTT	QTL
SB1936_F	CCTCTCTCTCTCTCTCCCAACACG	QTL
SB1936_R	CATGAATTCTTCGCATCATTCCAC	QTL
Xtxp31_F	ATGCGAGGCTGCCCTACTAG	QTL
Xtxp31_R	GTTTGGACGTACCTATTGGTGC	QTL
SSR2_1992_F	ACGCATCAGGAATTCACTTTAGGCT	QTL
SSR2_1992_R	GTTTGTGCAAAGTACGATGGATCAGGT	QTL
SSR2_2078_F	ACTTAATCAATCACACCTCGCTCCC	QTL
SSR2_2078_R	GTTTCTTTTCCACACCAAACCATGAGA	QTL
SSR3_2122_F	AGAAGAGCCCAAGGAAGGGAAGATA	QTL
SSR3_2122_R	GTTTAAAAGTGACAGACAACCCGTAGCC	QTL
SSR4_2129_F	ACGGGAAAATTCTTGATCATGGTG	QTL
SSR4_2129_R	GTTTTGGAGAGGCCTCATATTTGGA	QTL
Xtxp285_F	ATTTGATTCTTCTTGCTTTGCCTTGT	QTL

Xtxp285_R	GTTTGTCATTTCCCCCTTCTTTCTTTT	QTL
Xtxp70_F	AGTGACCTTAGCACCAAGCTC	QTL
Xtxp70_R	GTTTCAGGTAGCACTAGAG	QTL
SSR3_2362_F	ATGCAATCTATAGCAGCTGGAGACG	QTL
SSR3_2362_R	GTTTGTATGCATTTGCAAGCAGTGTGT	QTL
SSR2_2435_F	ATAGCTAGCTGCGTAATGACGAGGA	QTL
SSR2_2435_R	GTTTCGAGCATCGTCGGAAAATATACA	QTL
Xtxp26_F	ACAAGTGTAGTAGCAGTTTAGTCTC	QTL
Xtxp26_R	GTTTAGGTATCAAAGGACCAAGG	QTL
SSR5_2513_F	AGATGAGCCATATTTGGCAAGGAA	QTL
SSR5_2513_R	GTTTCAGTAAAGCTGGCTAGCTGCAACA	QTL
SSR2_2565_F	ACTGCACGCCTAATTATCCCTTGTC	QTL
SSR2_2565_R	GTTTGCTGCTGCTGTCCAGCTACTATCA	QTL
SSR3_2599_F	ATCTGGAGTCTGGAAGAATGCTGG	QTL
SSR3_2599_R	GTTTCCACTAGTGCATCAGCACA	QTL
Xtxp343_F	ACGATTGGACATAAGTGTTC	QTL
Xtxp343_R	GTTTATAAACATCAGCAGAGGTG	QTL
SSR5_2635_F	AGTGTCCGGCCTTTCTTGTGTTAG	QTL
SSR5_2635_R	GTTTGAGCAGTGCTGTTTGGAATTAGGA	QTL
SSR5_2660_F	ACAGCCCCCTTTCATATGTTCAAAA	QTL
SSR5_2660_R	GTTTATGGAGCAGGCTACTAGTGTCGGG	QTL
SSR5_2772_F	AGATGCAACCAAACCAACATAGCTG	QTL
SSR5_2772_R	GTTTAAAGCCAAGAAGGAAGACCCAAAG	QTL
SSR2_2831_F	ATTCAGCTTTGGATCTCGATCTGTG	QTL
SSR2_2831_R	GTTTCGTCAAATTCTAGCTCCTATCATCA	QTL
SSR3_2963_F	AGAGGGAGAGTGTGGATGCTGGTAT	QTL
SSR3_2963_R	GTTTGAAATCTGAGCCTAGGAGGGTGGT	QTL
SSR2_3016_F	AGGAAAAATGAGAAAAGAAACGGGC	QTL
SSR2_3016_R	GTTTGTCTCTGATTGTCAATGCCTCCCT	QTL
SSR2_3045_F	ATTGGCCATTTGACCTGCTAAAAG	QTL
SSR2_3045_R	GTTTAACATACCAAAGCCGCCGTATC	QTL
SSR4_3052_F	ATGGTAGTTTCTTCTTCCCCAACCC	QTL
SSR4_3052_R	GTTTAATTAAGAAACAAAACCCGGAGGG	QTL
SSR4_3056_F	AGACGCCATCTACTCCATCCTCCTA	QTL
SSR4_3056_R	GTTTATCTACGTCTGGATCGTCACCCTC	QTL
SB3067_F	ATGAGCAGCAGAAGCAATCATCAA	QTL
SB3067_R	TGTTTCAGTTATGTGTGTGGTGGA	QTL
SB3077_F	TGTTTCCTTTGTGTACGGTGTTGC	QTL
SB3077_R	GCCATCTGATGCTACGCTGTAGTG	QTL
SB3134_F	GGATGCAGGAAGAAGCTAAAGCAA	QTL
SB3134_R	CTGCAATGCACAGTTTCAGAGTGA	QTL
SB3146_F	CCCTCCTGTTCCTTATTTAGGTTTTCA	QTL
SB3146_R	ACCACCCACCAAAATGAATGTTTC	QTL
SB3160_F	GGAATCTGGAACTTGATTGGCTCA	QTL
SB3160_R	GTACAAGTTGGAACACAAGGCACG	QTL
SSR3_3178_F	ATTCTCTGCTCCGTTTCCTAACAGG	QTL
SSR3_3178_R	GTTTCTCGATTCATCTCATCGTCTTGG	QTL
SSR2_3183_F	ACCTTTGCACTTGATCACTTTCCCT	QTL
SSR2_3183_R	GTTTCTTCCTCGCCAACTTCTCATC	QTL
SSR3_3251_F	AGGACACCACACCGGATCTATAAGG	QTL
SSR3_3251_R	GTTTCCACCCACCACTACATGAAGGAAT	QTL
SSR5_3411_F	AGCTCGCATACTAGCTCATCGTTCA	QTL
SSR5_3411_R	GTTTGACTGTCCATGCGATCAGTAGGAA	QTL
YUC7_F2-R1_F	CTAGCTCATCGTTCACCACC	QTL
YUC7_F2-R1_R	GGGAGAAGAGACTGTCCATG	QTL
SSR2_3417_F	ACACTCTCTCCCACCTTCTCATCAA	QTL
SSR2_3417_R	GTTTGCTGGATTACCACAAGGCTATGA	QTL
SSR5_3423_F	AGTCCTATAAGACCTCGCCTTCGC	QTL
SSR5_3423_R	GTTTACGTTCTAGGAGTTGTCCCGGAG	QTL
Xtxp6_F	ATCGGATCCGTCAGATC	QTL
Xtxp6_R	GTTTCTAGGGAGGTTGCCAC	QTL
SSR3_3479_F	ACTGGCGTGGCAAGTTCAGTCT	QTL
SSR3_3479_R	GTTTGTTGCTGTGTTTTGGAGAGTTGA	QTL
SB24905_F	GGCACCGTATGTCTGCATGCTTAT	QTL
SB24905_R	TGGATCCAATTACTTCCTCTGTCA	QTL
SSR2_3489_F	ACAATAAATGCCGGTTTTCTGGTGAG	QTL
SSR2_3489_R	GTTTGCATGCCACTTTCTCTCTCATGT	QTL
SB3484_F	GCGACAGCGATCGGTGATATAATAA	QTL
SB3484_R	TATGCTTTTCATGGGAATGTGTGG	QTL
SB24977_F	ACAGACAGACATGCACGAAGAAGG	QTL
SB24977_R	CATGGAATCCGCGAGATACTTTTT	QTL
SB24991_F	GTCGCCACATCCAAATCATCATAG	QTL
SB24991_R	GTTGGCCTGGTATGGCAAACTTTA	QTL
SSR4_3503_F	AGTCCCTTGTTCGACTCCGTATCC	QTL
SSR4_3503_R	GTTTAACTACACCACCACCGTCGACAT	QTL
SB25027_F	TGCGATGCTAAAAATGAGTTGTGC	QTL
SB25027_R	TAGACTAGAGGTCTGGGCGGAGAG	QTL
SB25034_F	GCCTCACAACAACACAAAATCAGG	QTL
SB25034_R	CCCATGTTGGGTTTCAACTCTAGC	QTL
SB3507_F	ACTCTTCTCTCTCCCAGTCCCACG	QTL
SB3507_R	GCGCCTCTACAGGCTACAGGTATT	QTL
SB25093_F	GAGAGGGTGCAGGGAATAGGAAGT	QTL
SB25093_R	TAGATCCATACCCCTTCAGGAGCC	QTL
SB3517_F	CGAATCAGTTTTGTGCATCTTCGT	QTL
SB3517_R	AGTGCCGGAATCCTAGAAAGAAGG	QTL
SB25162_F	CGCGTAAGTCACCAAACAAACACT	QTL
SB25162_R	AACCTGTCTTCAGCACCCAGATTC	QTL
SSR5_3535_F	ATGAATTGCGTGGAAGATGAAGGT	QTL
SSR5_3535_R	GTTTGCCATTACGTACTACGCCTATCG	QTL
SB3547_F	AGACATCTCCCTCTCCATGATGCT	QTL
SB3547_R	GCCGCATCAGCAAAATACCATAAA	QTL
SB3552_F	TCCACCTGGCTCTCACTTTCTAGG	QTL
SB3552_R	ATCATGCATGTCGTTTCTGATCGT	QTL
SB3564_F	TGAAAACAGGAGGAAGAGGAATCG	QTL
SB3564_R	CCAGAGAAACCATCTATGTGCGTG	QTL
SSR2_3577_F	AGAAAAGAAGGTTCCTCCTGCCATT	QTL
SSR2_3577_R	GTTTACATCCACCCTTCTCTTGGTCAAA	QTL
Xtxp145_F	AGTTCCTCCTGCCATTACT	QTL
Xtxp145_R	GTTTCTTCCGCACATCCAC	QTL
Xtxp274_F	AGAAATTACAATGCTACCCCTAAAAGT	QTL
Xtxp274_R	GTTTACTCTACTCCTTCCGTCCACAT	QTL
SSR5_3737_F	ACTGACAGAAACATGCATGGGGTTA	QTL
SSR5_3737_R	GTTTAAACGACGCACTTGGTCAGAAA	QTL
SSR4_3805_F	ACAAGTTGGAGTTTCATCAGCATCG	QTL
SSR4_3805_R	GTTTACCAAAGGTATGCAGGGACCAC	QTL
SSR2_3870_F	ATAATAATGCTGCATGCTGAGTGCC	QTL
SSR2_3870_R	GTTTCCATTGTGTTTCTGATAGCTTGACAG	QTL

Xtxp159_F	ACCCAAAGCCCAAATCAG	QTL
Xtxp159_R	GTTTGGGGGAGAAACGGTGAG	QTL
SSR5_3966_F	ATCAGTTCATCTCCTTTTGGGTGC	QTL
SSR5_3966_R	GTTTACTTCACAGGGTCACAGCACAGTC	QTL
SSR5_4016_F	AGACAAGAGAAATCCCGTAAACCCC	QTL
SSR5_4016_R	GTTTGTACCTCAACCAGACGCTCGTCAC	QTL
SSR2_4043_F	AGCAAATTTTGGGGTGTTACATCGT	QTL
SSR2_4043_R	GTTTGTTGTGGTTGTTGCTGTTGTTG	QTL
SSR5_4072_F	ACTACCTGCGGTGGAAAGAGTCGAT	QTL
SSR5_4072_R	GTTTGAGTACGAACGTCTCGGCTTGTC	QTL
SSR3_4143_F	ATCTCCTTTAATTACGACCGACCGA	QTL
SSR3_4143_R	GTTTCTCCATGGATCGACCCTTTTGTT	QTL
SSR3_4197_F	ACGATCGAGTTTTTCTTGTGGTGTTC	QTL
SSR3_4197_R	GTTTCATGCATCCATGTTCGTCTTCTCT	QTL
SSR4_4230_F	ATTCATCAACCCTTTGTCAACCCTT	QTL
SSR4_4230_R	GTTTCGTTAGAAATCACCGCTAACTGGG	QTL
Xtxp273_F	AGTACCCATTTAAATTGTTTGCAGTAG	QTL
Xtxp273_R	GTTTCAGAGGAGGAGGAAGAGAAGG	QTL
SSR4_4339_F	ACAAAAGGGGAAAGAAACCAAGCTC	QTL
SSR4_4339_R	GTTTCACCACAAAGGGGAAAGGAAAATA	QTL
SSR4_4358_F	AGCCTCCTCAGTCACAAAACTCACA	QTL
SSR4_4358_R	GTTTCGTCCTTCCCGTCTAGAATTAGC	QTL
SSR4_4433_F	AGCTTGCTGGAGGTGAAGTACCC	QTL
SSR4_4433_R	GTTTAGGCCACCAAGTAAGCAGTGTACC	QTL
SSR3_4470_F	ATGCAGACATCTGAATTACGGTGGT	QTL
SSR3_4470_R	GTTTCAGGGAAGACTGAACTACGGGAG	QTL
SSR2_4480_F	ACCATAGCTGCGAGCAACCTGAG	QTL
SSR2_4480_R	GTTTGTCAGCCTTTAGATGAGGCCAGAA	QTL
SSR5_4493_F	AGATGCAGACGACATGGCTAGAGAA	QTL
SSR5_4493_R	GTTTAAATTCCGGTACCTCAATCGCC	QTL
Xtxp354_F	ATGGGCAGGGTATCTAACTGA	QTL
Xtxp354_R	GTTTGCCTTTTTCTGAGCCTTGA	QTL
SSR2_4533_F	ACAAGGAGAGAGAGAGAGGGGGCAAAAG	QTL
SSR2_4533_R	GTTTCACGATCGTCTCTCTGGCTGTCTA	QTL
SSR3_4586_F	ATCCTACGTAGCTACTCCAGCAGGC	QTL
SSR3_4586_R	GTTTCTGCAGGGTACGTAACTCCTACGC	QTL
SSR5_4642_F	ACAGAGAGGTGGAGGAGGCAGTTAG	QTL
SSR5_4642_R	GTTTATCATGTCCACCACCCATCTTTTT	QTL
SSR4_4687_F	ATCATTTTCATTCACCCCACGAAC	QTL
SSR4_4687_R	GTTTATGCAGCAAGTACACAACTGGGAC	QTL
SSR3_4688_F	ACTGTAAGCATGATGAAGGTCGTGG	QTL
SSR3_4688_R	GTTTAAGAAGGTGATGACAGGGATGGAG	QTL
SSR3_4706_F	ATTGCTTGCTCCTCCTGGGACT	QTL
SSR3_4706_R	GTTTGTTGCCTTTGCCTAGCTTTCCTTT	QTL
SSR3_4711_F	AGGACCTGCACTTCGATGGTGT	QTL
SSR3_4711_R	GTTTGGGACGACGCTAACTGTCTCCTTA	QTL
SSR3_4715_F	ACAATTCGAGAAGAGCTGGTGAGTGG	QTL
SSR3_4715_R	GTTTAGTGCGCGTGAGTAGTGCAT	QTL
SSR3_4764_F	ATAATTAGTTAGGCCCACACGCACA	QTL
SSR3_4764_R	GTTTGAGGCTGACCGTCTTCATCTCTGT	QTL
Xtxp258_F	ACACCAAGTGTCGCGAACTGAA	QTL
Xtxp258_R	GTTTGCTTAGTGTGAGCGCTGACCAG	QTL
SSR2_4795_F	AGACGACTGCACTTGTTGCTTGACT	QTL
SSR2_4795_R	GTTTCTTGACACCACCAAGAAACCACAG	QTL
Xtxp67_F	ACCTGACGCTCGTGGCTACC	QTL
Xtxp67_R	GTTTCCACACAAGATTCAGGCTCC	QTL
SSR5_4851_F	ACAATCTTAAGGGGTCTACCAGGGCA	QTL
SSR5_4851_R	GTTTATGGAGAGGGCATGTAATGTTGGT	QTL
SSR5_4888_F	ACGTCAAAGCAAGTTTCACTTAGAAC	QTL
SSR5_4888_R	GTTTCTCCACGGTGAATCTAATAACACATCA	QTL
SSR3_4902_F	ATACAAAGGGAATGCCTCTCTCCC	QTL
SSR3_4902_R	GTTTACACACATCGAGGAAAATGTGGG	QTL
SSR5-5007_F	AGGAACTTGTTGCTGGTGTAGCAGA	QTL
SSR5-5007_R	GTTTGCTGCCTCTTCGTACTGTGAGTGA	QTL
SSR5-5031_F	ACTGCACCTTGCTTAGAACCCACTT	QTL
SSR5-5031_R	GTTTGGTATATATGGGGAAGCGTGCGTA	QTL
SB5019_F	AAAGAGAAGGGAGAGGGGAAAAGC	QTL
SB5019_R	GTGGAGCTTGTCCGAGATCTTGTC	QTL
SB5028_F	TTGCATCCCTAAAAGCATTTCTGC	QTL
SB5028_R	GCCATTGATGGCTACGTAAATTCC	QTL
SSR2_5042_F	ACAGTAACCACCACCAGAACGCTT	QTL
SSR2_5042_R	GTTTCGCTTCTCTTCCCTTGTGTCTT	QTL
SSR4_5057_F	ACCGGCACATTTACTCACTTCCTCT	QTL
SSR4_5057_R	GTTTACGATTCCACCGAACCAAGAGTT	QTL
SSR4_5059_F	ACAGTACTGTACGACGTAGGAGACGC	QTL
SSR4_5059_R	GTTTACTCCGGACGACGCTAATACAAG	QTL
SSR5_5061_F	ACAACCTCTCCATATCCGAGGTACG	QTL
SSR5_5061_R	GTTTCGGGGAACTCAATTATTCGTTTTG	QTL
Xtxp107_F	ACAAAGTGAGCGTGGTC	QTL
Xtxp107_R	GTTTGGACAGGGATAACATAACATA	QTL
SSR3_5111_F	ACAAACCAACCCGACCCACTCTTTAT	QTL
SSR3_5111_R	GTTTCGGTTCGTTCCATCAGATTCTTCT	QTL
SSR5_5159_F	ATATGCTTCCAATACAGAGGGACCG	QTL
SSR5_5159_R	GTTTGTACGCCAGGAGATGTGATATGG	QTL
SSR5_5216_F	ACATGTCCTTGTTGATGTCGATGCT	QTL
SSR5_5216_R	GTTTCCACCATCGGTTCCTAGCTG	QTL
SSR3_5242_F	AGCTACAAACATCTGAGACTGCCGA	QTL
SSR3_5242_R	GTTTCAGAGGACCCTGTTTCGTTTGAT	QTL
SSR3_5298_F	ACCTGCTAACGCTGCCGTACAC	QTL
SSR3_5298_R	GTTTGTCACCTCCTTCATCCTTGTCGTT	QTL
SSR2_5303_F	AGAGAGAGGGGAAAGAAAAAGGGC	QTL
SSR2_5303_R	GTTTGTTCCACATCGATTCGTCTCCTCT	QTL
SSR5_5423_F	ATCCGCTATAAATTGGAGAGGACCC	QTL
SSR5_5423_R	GTTTAGCCGCAGTAAGCTCACTAGGAAA	QTL
SSR5_5460_F	AGAGAACGGAGACTGAAGGTTGGAA	QTL
SSR5_5460_R	GTTTGCTGCCCCTTAAACTATTCGCTCT	QTL
Xtxp141_F	ATGTATGGCCTAGCTTATCT	QTL
Xtxp141_R	GTTTCAACAAGCCAACCTAAA	QTL
SSR2_5529_F	ACAGCAAATTTCAGGTGGTTTTTGAC	QTL
SSR2_5529_R	GTTTATTATATCTCTGCGTGCAACGGCT	QTL
SSR2_5543_F	ACAACCCCCTTGAACTCGAAGAAGAC	QTL
SSR2_5543_R	GTTTAGTGCCAATCCTCTCTCACTCCAC	QTL
SSR3_5556_F	AGCTTCAAAGATGGCCAAAACAGA	QTL
SSR3_5556_R	GTTTGCAGAGCCACAAGTCAAGGTGTTA	QTL
SSR2_5560_F	ACCACCAGATCGATTATTCAGAGGC	QTL
SSR2_5560_R	GTTTCCAGCCTCTAACTCCCTACCACCT	QTL
SSR4_5576_F	ACACGTGTTCTTCGTCGTTCCTATG	QTL

SSR4_5576_R	GTTTCTCATCACGGATTCACGATACAGC	QTL
Xcup07_F	ACTAGAGGATTGCTGGAAGCG	QTL
Xcup07_R	GTTTCTGCTCTGCTTGTCGTTGAG	QTL
SSR2_5603_F	ACAATTCTGCTGCACACTTCAGATGC	QTL
SSR2_5603_R	GTTTCTGCGACCGTCTCACAACAATTAG	QTL
SSR_chr.9_57167_25_F	GTTCGTTCGTCTGGAGAGGTGCTGG	fine mapping
SSR_chr.9_57167_25_R	GCCCGGCATCCAAATTCCTAATCCG	fine mapping
SSR_chr.9_57184_F	GGCCTAGATTGTTCATGATA	fine mapping
SSR_chr.9_57184_R	TAAACACGGCCTAAGTCGAT	fine mapping
SSR_chr.9_57191_F	CTAGGTTAAAGCCGTCCGCC	fine mapping
SSR_chr.9_57191_R	AACCCTAACGAGTGGAGGAC	fine mapping
SNP_57209seq_F	GGCACGTACGTACAATCAAG	fine mapping
SNP_57209seq_R	ATAATGCGCGTATGCACCTG	fine mapping
SNP_57211seq_F	TCCTGCAGCACGTACTTCTG	fine mapping
SNP_57211seq_R	GATCTAGAGGTCTTCCATTG	fine mapping
SNP_57211_XbaI_F	TTCGCTGTTGGGCCATATGC	fine mapping
SNP_57211_XbaI_R	AAGGCATGTTATGCTCCTTGCTGGCAGTCT	fine mapping
SNP_57225_MboI_F	AGGCGCCAGAATGACTAGAG	fine mapping
SNP_57225_MboI_R	GGAGGAGGAAGGCCACGGCGATGACCGGAT	fine mapping
SSR_chr.9_57227_F	GTACTACCTGCTGTTCGTCG	fine mapping
SSR_chr.9_57227_R	CTTCTGTACAATACGGGTTG	fine mapping
57241seq_F	CGGAGCGTCTGAGATCTGAC	SNP sequnencing
57241seq_R	CCGCAGCATTGAATTGGGAC	SNP sequnencing
TA57249seq_F	GATGATCGGTGGAGATAGTC	SNP sequnencing
TA57249seq_R	ATGGATGCACATCTGTCAG	SNP sequnencing
TA57249seqb_F	GAGATAGTCTTACGGCTAGG	SNP sequnencing
TA57249seqb_R	CTCTCTTATGAATCTGTCAG	SNP sequnencing
TT57259seq_F	CTTCCCAGCTGCATGCAATG	SNP sequnencing
TT57259seq_R	TTAATTAGGCGTAGCATTGG	SNP sequnencing
SSR_chr.9_57337_F	TGACGCTTCGGTCGAACCAG	fine mapping
SSR_chr.9_57337_R	AACACAGGATGAGGACCATG	fine mapping
SbYUC7_F2	CTAGCTCATCGTTCACCACC	fine mapping
SbYUC7_R1	GGGAGAAGAGACTGTCCATG	fine mapping
Sb09g028270_F1	ATTTGCAGCTGGATCTGATC	sequnecing (*Sobic.009G229800*)
Sb09g028270_R1	GAACATACGAAATGTTAGTG	sequnecing (*Sobic.009G229800*)
Sb09g028270_F2	ACACCTGGGATAATCCTTGC	sequnecing (*Sobic.009G229800*)
Sb09g028270_R2	TGAACCACCAGAATAGGCTG	sequnecing (*Sobic.009G229800*)
Sb09g028270_F3	CCAGATGTTCCATATGCTCG	sequnecing (*Sobic.009G229800*)
Sb09g028270_R3	ACCTTTGAACACGTCCAAGC	sequnecing (*Sobic.009G229800*)
Sb09g028270_F4	GATAAATCATGTGCCTACGC	sequnecing (*Sobic.009G229800*)
Sb09g028270_R4	GATTGATTGTGTTACACAAG	sequnecing (*Sobic.009G229800*)
Sb09g028270_F5	GGCACGTACGTACAATCAAG	sequnecing (*Sobic.009G229800*)
Sb09g028270_R5	ACTGAAGACATCTCTGACTG	sequnecing (*Sobic.009G229800*)
Sb09g028270_F6	AGGAGTTACTACAATCTGAG	sequnecing (*Sobic.009G229800*)
Sb09g028270_R6	CAAATAATCTACGTGTCTTC	sequnecing (*Sobic.009G229800*)
Sb09g028270_F7	CGTGTCTGACTACTCAGATC	sequnecing (*Sobic.009G229800*)
Sb09g028270_R7	GCACATGCTTTCTCATGACG	sequnecing (*Sobic.009G229800*)
09g028310_1F	GGCGACTGGTTCAACTTCAA	sequnecing (*Sobic.009G230200*)
09g028310_1R	GGCCGTACACTTGATAAATC	sequnecing (*Sobic.009G230200*)
09g028310_2F	ATTACGAGAAATTATTCAAG	sequnecing (*Sobic.009G230200*)
09g028310_2R	GCCCGAGATTGGCATCTCAG	sequnecing (*Sobic.009G230200*)
09g028310_3F	AGAGTAGCTAGTTAGATTCG	sequnecing (*Sobic.009G230200*)
09g028310_3R	CCTTGCCATGGCGCATAACG	sequnecing (*Sobic.009G230200*)
09g028310_4F	CTACTTCCAGAACGCCTTCG	sequnecing (*Sobic.009G230200*)
09g028310_4R	AGGTTCACGAACGGGTACAG	sequnecing (*Sobic.009G230200*)
09g028310_5F	GTTCATCATGTTCGGCGGTC	sequnecing (*Sobic.009G230200*)
09g028310_5R	CAAGGTGACCTTCACGTCGG	sequnecing (*Sobic.009G230200*)
09g028310_6F	GCCGTACCTCAGCGATGATG	sequnecing (*Sobic.009G230200*)
09g028310_6R	GGACCAGAATGAAACATCCA	sequnecing (*Sobic.009G230200*)
09g028275_ex1_RT_F	TCAGAAGGTGGATAAATCATGTGCCTACGC	RT-PCR
09g028275_ex2_RT_R	CTCGATTTCTGCATCTGAGCAAGAATGGCC	RT-PCR
028280ex2_RT1_F	CTCTGCTCTTGCCCTGTCCA	RT-PCR
028280ex4_RT2_R	TCATTTGTAGATCCTGACGC	RT-PCR
09g028290_3'UTR_RT_F	ACCGTGAAGCGGGCGGTGGAGATACTGGAG	RT-PCR
09g028290_3'UTR_RT_R	TCGTGCCCCTGTCACGGCCCAGACAGGTAC	RT-PCR
09g028300_3'UTR_RT_F	GACCGCGGAAGGAAGCTAACTTGGCAAGAC	RT-PCR
09g028300_3'UTR_RT_R	GCCAGAACACCAGCACCTTCAGAGTTTCAG	RT-PCR
09g028310_ex1_RT_Fb	CATGGCAAGGCCGTCATGTTCGATACCAGC	RT-PCR
09g028310_ex2_RT_R	CAGCCAGTCGCAGGTGTCGCACGGGCTCAG	RT-PCR
sb_01g030340_F	GGTTCGGGAGGTGGCCTAGGT	RT-PCR (sorghum ubiquitin)
sb_01g030340_R	AGCATGTACATTCCCAGCGGTA	RT-PCR (sorghum ubiquitin)
Sb_PRR37_F1	GCTTATTTGACATGGCAATG	sequnecing (*SbMa1*)
Sb_PRR37_R1	GACAGCATAGAGCTGTGAAC	sequnecing (*SbMa1*)
Sb_PRR37_F2b	GCTAGCTAGGAATAAGTTAC	sequnecing (*SbMa1*)
Sb_PRR37_R2b	ACCGTTAGAGCAGAATAATG	sequnecing (*SbMa1*)
Sb_PRR37_F3	CACGGAACGAGTGTGGTTGG	sequnecing (*SbMa1*)
Sb_PRR37_R3	CACCATCGTCATCATCCTGG	sequnecing (*SbMa1*)
Sb_PRR37_F4	ACTAATCTCGACGAACTACG	sequnecing (*SbMa1*)
Sb_PRR37_R4	GGATGATGCTTCGGAATAAC	sequnecing (*SbMa1*)
Sb_PRR37_F5c	GCCACCTCTGACACGTAG	sequnecing (*SbMa1*)
Sb_PRR37_R5c	CTAACTCAGATAGCTAGGTG	sequnecing (*SbMa1*)
Sb_PRR37_F6	GCCACTACGATAGTATATAC	sequnecing (*SbMa1*)
Sb_PRR37_R6	ATCCTCCTTGGACCATGCAC	sequnecing (*SbMa1*)
Sb_PRR37_F7	CGGCAAGAGGATAGATCTAG	sequnecing (*SbMa1*)
Sb_PRR37_R7	AGAGAAGCCAAGTGCTCTTG	sequnecing (*SbMa1*)
Sb_PRR37_F8	ACTGTTCTCCAACAATAAGC	sequnecing (*SbMa1*)
Sb_PRR37_R8	TCTTGCATATCACAGGACAC	sequnecing (*SbMa1*)
Sb_PRR37_F9	GACTTTGAATCTTTAAGGCC	sequnecing (*SbMa1*)
Sb_PRR37_R9	ATGGTATACTACTCTTTCGC	sequnecing (*SbMa1*)
Sb_PRR37_F10	GGTAAGAATGAAATTACCTC	sequnecing (*SbMa1*)
Sb_PRR37_R10	ATAGTGTGAGTCTCTTCGCC	sequnecing (*SbMa1*)
Sb_PRR37_F11	TCTAATACTCTTCACCGTTG	sequnecing (*SbMa1*)
Sb_PRR37_R11	GTCAAACTTACCACTTGTGG	sequnecing (*SbMa1*)
Sb_PRR37_F12	CATAGGTTTGGCTACCATAG	sequnecing (*SbMa1*)
Sb_PRR37_R12	CCGTCTCCATACATGCTGCC	sequnecing (*SbMa1*)
Sb_PRR37_F13	TCGAATGATGCTAGGAATAC	sequnecing (*SbMa1*)
Sb_PRR37_R13	AGAATTCACTGCAGACCTAG	sequnecing (*SbMa1*)
Sb_PRR37_F14b	TGGTCAATCCACATAGCAGC	sequnecing (*SbMa1*)
Sb_PRR37_R14b	TCTCGTACGGCATTCCATGC	sequnecing (*SbMa1*)
Sb_PRR37_F15	GTATTCCGAGAATAGTGTAC	sequnecing (*SbMa1*)
Sb_PRR37_R15	GAAGTCGTCTGGAATCAACC	sequnecing (*SbMa1*)
Sb_PRR37_F16	TGGTACTGATTCACTAATGC	sequnecing (*SbMa1*)
Sb_PRR37_R16	AGGGCAACATTACCCTGTCC	sequnecing (*SbMa1*)

Sb_PRR37_F17	AATGACATGGGTTCCACTAC	sequnecing (*SbMa1*)
Sb_PRR37_R17	AGTCCTGCCCAATATAATGC	sequnecing (*SbMa1*)
Sb_PRR37_F18	TGCATGTTTGTGGTTCAGGC	sequnecing (*SbMa1*)
Sb_PRR37_R18	AGCTAACACAACTGCTGTTC	sequnecing (*SbMa1*)
Dw3_F1	GCTCTGCGCCAGCCACTCTG	sequnecing (*Dw3)*
Dw3_R1	TGAGCGACTGATGTGATTCG	sequnecing (*Dw3)*
Dw3_F2	CGTCCTCATGGGCAGGTAAC	sequnecing (*Dw3)*
Dw3_R2	CGATCATGACGGAGAACATG	sequnecing (*Dw3)*
Dw3_F3	TCGGTCACCGCAGCGGCTTC	sequnecing (*Dw3)*
Dw3_R3	AATGTACGCCTGCTACAGTG	sequnecing (*Dw3)*
Dw3_F4d	GAGTAGGAGTGGTTCAATTG	sequnecing (*Dw3)*
Dw3_R4d	GGCAGGCAGGCATGGTGGTC	sequnecing (*Dw3)*
Dw3_F5	GCAATGCTCGCATGCCCATG	sequnecing (*Dw3)*
Dw3_R5	ACTCGGGCGAGTTCATCCTG	sequnecing (*Dw3)*
Dw3_F6	TTCTCCACCTCCGACTTCAC	sequnecing (*Dw3)*
Dw3_R6	TCTTGCGCTCCGCGTTGAAC	sequnecing (*Dw3)*
Dw3_F7	TCCGTCATCGTCCAGAACTC	sequnecing (*Dw3)*
Dw3_R7	GCCCGCAGGTTGTACTTGCG	sequnecing (*Dw3)*
Dw3_F8b	AGCACGTGGACTTCTCGTAC	sequnecing (*Dw3)*
Dw3_R8b	TGCGATCATGGAGTACCATC	sequnecing (*Dw3)*
01g0103800RNAi_EcoRV_F	CGATATCGCCAATTCCAACCAAAGTGCG	RNAi construction
01g0103800RNAi_SmaI_F	TCCCCCGGGGCCAATTCCAACCAAAGTGCG	RNAi construction
01g0103800RNAi_XbaI_R	GCTCTAGAGACCTCTTGCTCAGGGATGGGC	RNAi construction
01g0103800RNAi_SpeI_R	GGACTAGTGACCTCTTGCTCAGGGATGGGC	RNAi construction
09gRNAicheck_F1	CCGGATGCCGACGCGAAGCG	RNAi construction
09gRNAicheck_R1	CTGAGGCTCGTTAGCATACG	RNAi construction
5-liker_MCS-Nos 3 UTR_F	TGTGAATTACAGGTGACCAGCTCG	RNAi construction
5-Nos 3 UTR_R	TATCGCGTATTAAATGTATAATTGC	RNAi construction
Os01g_RT_F	TTTCCGGAGAATGTGACCGC	RT-PCR (*Os01g0103800*)
Os01g_RT_R	AATTAGGTGATTGGGCAGTC	RT-PCR (*Os01g0103800*)
Os03g_RT_F	GAATGTGACCGGAGCAACCG	RT-PCR (*Os03g0270700*)
Os03g_RT_R	AGAAGGACGCCGGAGAAGAT	RT-PCR (*Os03g0270700*)
Actin_F	CATCTTGGCATCTCTCAGCAC	RT-PCR (rice actin)
Actin_R	AACTTTGTCCACGCTAATGAA	RT-PCR (rice actin)
SSR_chr.9_57179_F	TAAGATTCTCCGTCACATAG	NIL genotyping (*Dw1*)
SSR_chr.9_57179_R	ATAGACCGTAAAGTTGCACG	NIL genotyping (*Dw1*)
SSR_chr.9_57354_F	TTTCGGATACTGTAACCGAC	NIL genotyping (*Dw1*)
SSR_chr.9_57354_R	TTTAGAGATTGTCGTGTCGC	NIL genotyping (*Dw1*)
SSR3_4143_F	ATCTCCTTTAATTACGACCGACCGA	NIL genotyping (*Dw3*)
SSR3_4143_R	GTTTCTCCATGGATCGACCCTTTTGTT	NIL genotyping (*Dw3*)
SSR3_4197_F	ACGATCGAGTTTTTCTTGTGGTGTTC	NIL genotyping (*Dw3*)
SSR3_4197_R	GTTTCATGCATCCATGTTCGTCTTCTCT	NIL genotyping (*Dw3*)
BAR_RBCter_F	CGACTCTAGAGGATCCATGGCAATTACCTTATCCGC	Sorghum complementetion
BAR_RBCter_R	GCAGGTCGACGGATCCCAAACATATAGTAGATGCGAC	Sorghum complementetion
pUbiin_BAR_RBCter_F	TCGGCGCGCCGGGCCTGCAGCGTGACCCGGTCG	Sorghum complementetion
pUbiin_BAR_RBCter_R	TATGGAGAAACTCGACAAACATATAGTAGATGCGAC	Sorghum complementetion
